# Flexible synthesis of anthracycline aglycone mimics via domino carbopalladation reactions

**DOI:** 10.3762/bjoc.9.258

**Published:** 2013-10-24

**Authors:** Markus Leibeling, Daniel B Werz

**Affiliations:** 1 Institut für Organische Chemie, Technische Universität Braunschweig, Hagenring 30, 38106 Braunschweig, Germany

**Keywords:** anthracyclines, carbohydrates, carbopalladation, catalysis, domino reaction, natural products

## Abstract

A synthesis of anthracycline aglycone derivatives is described. The key step utilizes a powerful domino carbopalladation approach and subsequent ring closure. During this process two of the four rings of the anthracycline scaffold are formed. Differently substituted carbohydrates and dialkyne chains serve as versatile and simple starting materials for the reaction sequence. Diverse building blocks lead to a variety of different products and a broad range of structural diversity.

## Introduction

**Figure 1:**
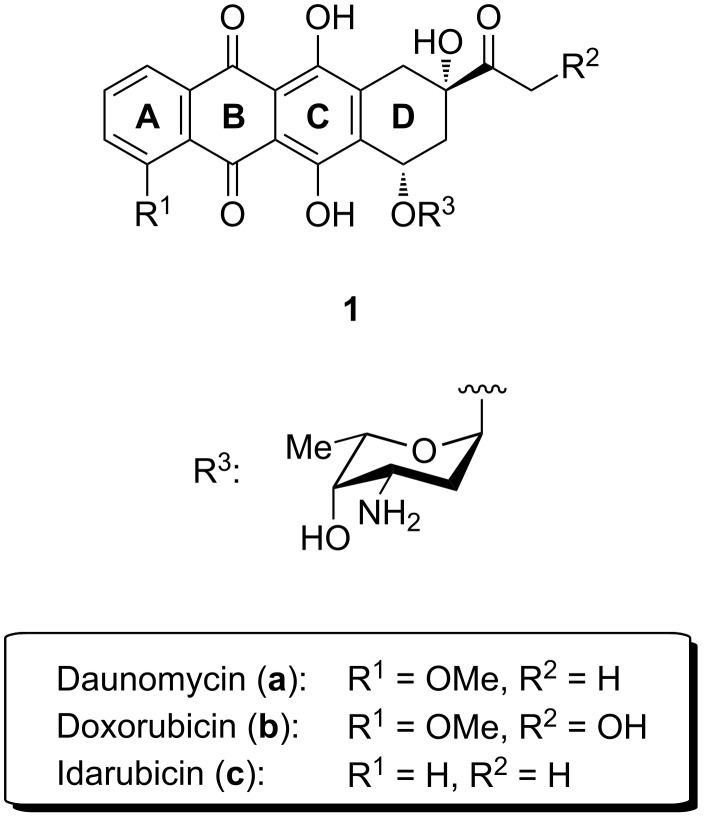
Several natural occurring anthracycline antibiotics.

Anthracyclines are a widespread class of natural products which belong to the group of aromatic polyketides [1]. Most of them have been isolated from bacteria of the order *Streptomycetales*. The group of Brockmann, who first found anthracyclines in 1963, described them as red to orange dyes [2]. Their structure elucidation revealed a linear fourfold annulated ring system including two benzene units (A-ring and C-ring). The substitution pattern of the D-ring bares most of the functionalities, i.e., a secondary and a tertiary alcohol, the former of which is commonly glycosylated with 2,6-dideoxy sugars (Figure 1) [3]. These carbohydrates are of highest importance for the biological activity of anthracyclines and bind to the minor groove of double-stranded DNA [4–5]. While the mode of action of anthracyclines is still not fully understood, it is widely accepted that these chemotherapeutic agents form a ternary complex with double-stranded DNA and topoisomerase II thereby leading to DNA damage and cell death [6–7]. They are used to treat different types of diseases such as leukemias, lymphomas, breast, uterine, ovarian and lung cancers [8].

Thus, many research groups faced the challenge of investigating suitable pathways for the synthesis of diverse anthracycline natural products and mimics thereof. Because of the inherent lack of efficient synthetic approaches to anthracyclines many industrial approaches still rely on the use of recombinant microorganisms with a mutated gene of the anthracycline metabolism [9].

**Scheme 1:**
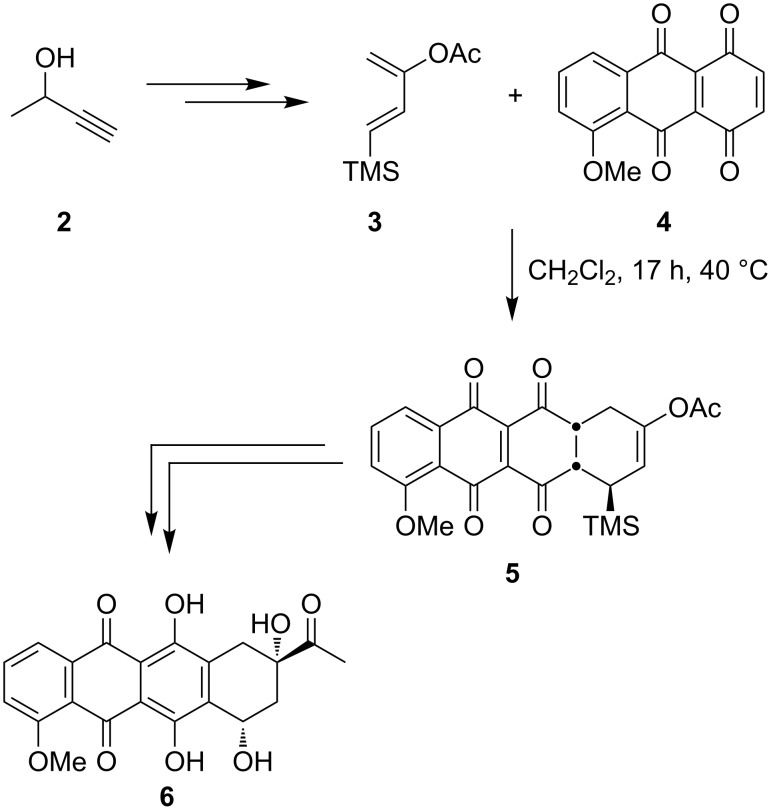
Total synthesis of daunomycinone **6** according to Hansen.

Different convenient synthetic transformations involve the application of a Diels–Alder reaction as key step for the aspired synthesis [10–13]. A classical synthesis was published in 1988 by Hansen where a silyl-substituted diene **3** was used for the [4 + 2]-cycloaddition (Scheme 1) [14]. Starting from bisquinone **4** the annulated ring system **5** is obtained in a 1:1 mixture of *cis-endo* regioisomers. Subsequent aromatization of the C-ring and several additional steps generated the daunomycin aglycon **6** and the corresponding isodaunomycin aglycone (dependent on the regioisomers) in a total of 16 steps.

**Scheme 2:**
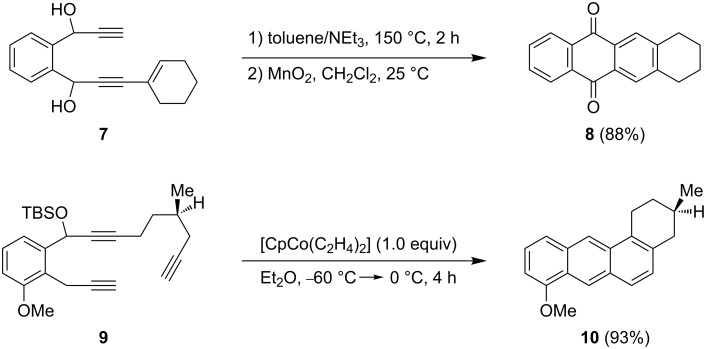
Synthesis of simplified anthracycline derivatives.

In 2003, Saá published a concise route to anthraquinone derivatives by using an intramolecular dehydro-Diels–Alder reaction of an aryldiacetylene system (Scheme 2) [15]. Compound **7** reacts at high temperature in a mixture consisting of toluene and triethylamine to an inseparable mixture of cyclized diol (52%) and quinone **8** (36%). Quantitative oxidation of the diol by MnO_2_ provided the desired tetracycle **8** in 88% overall yield (over two steps). Another approach to non-linear systems utilizes a cobalt-mediated intramolecular [2 + 2 + 2]-cycloaddition of a triyne system **9** leading to the fourfold annulated ring system **10** in only one step [16]. Late stage functionalization led to the anticipated structural motif in a few additional steps.

## Results and Discussion

### Retrosynthetic strategy

After having established several methods of domino carbopalladation reactions which employ dialkynyl-substituted bromoglycals [17–20] or bromoarenes [21], we envisioned to apply a similar procedure for the preparation of anthracycline derivatives. Therefore, the D-ring was exchanged for a pyranose, as described in our previous synthetic approaches for the syntheses of chromans, isochromans and biphenyls, respectively. These 2-bromoglycals **15** are well-known compounds and their synthesis was accomplished according to literature-known procedures [17–18]. The dialkyne unit provides both the A-ring and the information for the formation of the B and C-ring within the palladium-catalyzed domino transformation [22–27]. Such an approach should allow an easy differentiation between all four annulated cycles and their possible modification, whereupon the main focus was the preparation of several D-ring derivatives.

**Scheme 3:**
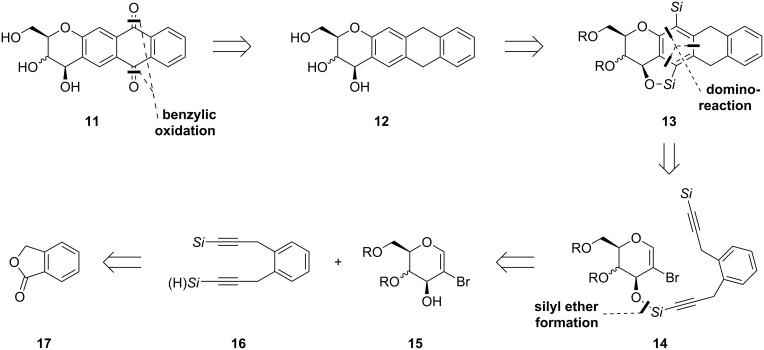
Retrosynthetic analysis of anthracycline aglycone mimics. *Si*: any silyl group.

The anthraquinone moiety **11** should be formed within the last steps of the synthetic approach by benzylic oxidation of compound **12** (Scheme 3). The terminal silyl group and the silyl ether should be removed by using hydrolysis and fluoride-mediated desilylation, respectively. It was assumed that the multiple carbopalladation/cyclization sequence should give access to the fourfold-annulated ring system **13** in a single step. However, we knew that the domino process works much better in an intra- than in an intermolecular fashion [19]. Thus, we decided to employ a silyl ether moiety to connect both subunits **15** and **16**. As the terminus of the other alkyne unit we also chose a silyl group. Depending on the kind of the silyl group a variety of further functionalization might be envisioned. Respective silyl-substituted diynes **16** can be traced back to phthalide (**17**). To differentiate between the insertion of two differently substituted silylacetylenes, the lactone **17** had to be first converted into a monoprotected diol for further transformations.

### Synthesis of dialkyne building blocks

**Scheme 4:**
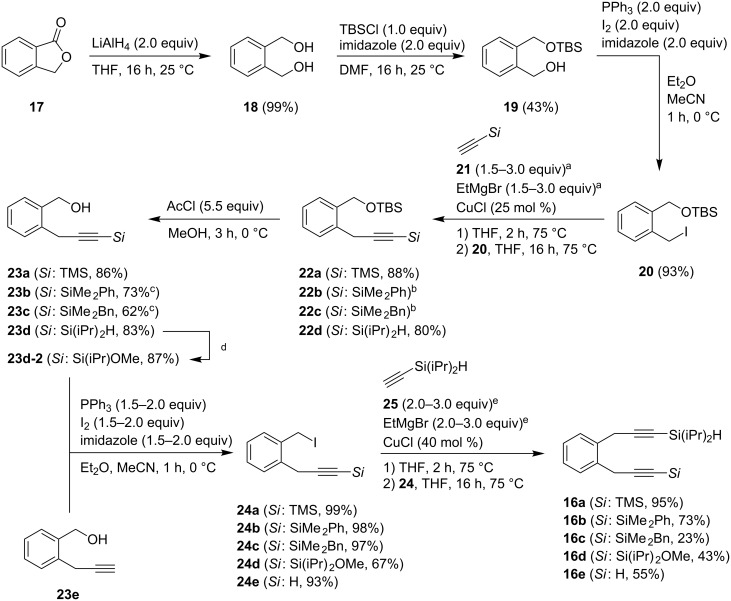
Synthetic route for the synthesis of various dialkynes **16**. ^a^*Si*: TMS, SiMe_2_Bn (2.0 equiv); *Si*: SiMe_2_Ph (1.5 eq equiv); Si(iPr)_2_H (3.0 equiv). ^b^Products for *Si*: SiMe_2_Ph and SiMe_2_Bn could not be isolated. ^c^Yield: over two steps. ^d^Conditions: Br_2_ (1.0 equiv), MeOH, 0.5 h, 0 °C, NEt_3_ (2.0 equiv), CCl_4_, MeOH, 1 h, 0 °C. ^e^*Si*: TMS, Si(iPr)OMe, H (3.0 equiv of **25**); *Si*: SiMe_2_Ph, SiMe_2_Bn (2.0 equiv of **25**).

The choice of the right diyne is crucial for a successful synthesis of the target compound. Preliminary investigations had shown that both dialkynes with benzylic hydroxy functionalities and 1,2-bis(2-propynyl)benzene did not yield viable results in the domino reaction. The selective installation of only one silyl group at a dialkyne with two terminal acetylene moieties presented difficulties. Thus, we sought for a consecutive introduction of the corresponding silylacetylene functionalities. An appropriate starting material was selected to achieve a highly convergent and convenient synthetic strategy. We started our investigation with the reduction of commercially available phthalide (**17**) by LiAlH_4_ into dialcohol **18** in quantitative yield [28] (Scheme 4). The polar compound was easily converted into the mono-TBS-protected substrate by utilization of 1 equivalent of TBSCl [29]. Column chromatography afforded three fractions consisting of the starting material, the monoprotected and the diprotected product. The remaining alcohol moiety of compound **19** was converted into the respective iodide **20** by a Mukaiyama redox-condensation using elemental iodine, triphenylphosphine and imidazole [30]. The installation of a suitable leaving group sets the stage for the introduction of the first silylacetylene. Four different terminal alkynes **21** (**a**: *Si* = TMS; **b**: *Si* = SiMe_2_Ph; **c**: *Si* = SiMe_2_Bn; **d**: *Si* = Si(iPr)_2_H) were employed. Best results with yields of over 80% were obtained by the use of acetylene **21a**, ethylmagnesium bromide, and copper chloride in tetrahydrofuran for one hour at 75 °C, successive addition of iodide **20** in THF at room temperature and additional 16 h under reflux [31]. Products **22b** and **22c** could not be isolated in pure form due to small impurities. The deprotection of the silyl ethers proceeded smoothly with high yields ranging from 62% to 86% over two steps [32]. Silane **23d** was converted into the corresponding silyl bromide and trapped with methanol to install an electron-deficient substituent at the silane moiety **23d-2**. The synthesis of **23e** was accomplished according to a literature-known procedure starting from isochromanone and trimethylsilyl-diazomethane [33]. The initially formed alcohols **23** were again converted into the respective iodides **24** as described before and subsequently substituted with diisopropylsilylacetylene **25** [34] providing five different dialkynes **16**, each of them with a terminal silyl substituent (or terminal hydrogen) at one side and a silane moiety at the other (Scheme 4). It is possible to access differently substituted dialkynes **16** by the silylation of **16e**. This approach was not considered because of the low tolerance of **16e** against base and the expensive starting materials for the synthesis of **23e**.

### Silyl ether formation and domino reaction

The union between both building blocks proved to be more difficult than originally envisioned. During our previous studies of chromans and isochromans the implementation of ether linkages afforded good results. The synthesis of anthracycline derivatives requires a more labile connection to circumvent the formation of an additional cycle at the annulated ring system. Previous investigations revealed that silyl ether formations could be accomplished by the transformation of the silane into the corresponding silyl bromide by using NBS [35]. This highly reactive species should be easily trapped by the hydroxy functionality of the respective 2-bromoglycal. Therefore, we chose **15a** and **16a** as model substrates to explore suitable reaction conditions for the silyl ether formation. Table 1 reveals that of the halogenated succinimides only NCS and NBS are able to convert the silane into a reactive species. However, the yields were low in all cases. Changing the bromination reagent to elemental bromine significantly improved the yields (Table 1, entries 4 and 5) [36]. Diethyl ether proved to be important as a solvent, the change to THF led to a total decomposition – most probably due to ring-opening reactions with bromosilanes [37]. Further, we investigated the influence of the amount of bromine on the reaction and concluded that stoichiometric quantities entirely fulfill the demands of the bromination (Table 1, entries 7 and 8). Finally, we found that the silyl bromide formation proceeded better in tetrachloromethane. However, to assure solubility of the glycals, small amounts of diethyl ether were added for the coupling step (Table 1, entries 8–10). In all cases, a very slow addition of bromine and bromosilane proved to be necessary to ensure optimal yields.

**Table 1:** Optimization study for the silyl ether formation.

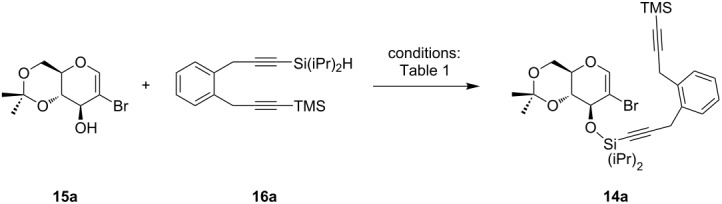		

entry	conditions^a^	yield [%]

1	1) **15a** (1.1 equiv), NBS (1.1 equiv). 2) **16a** (1.0 equiv), NEt_3_ (2.0 equiv), CH_2_Cl_2_	23
2	1) **15a** (1.1 equiv), NCS (1.1 equiv). 2) **16a** (1.0 equiv), NEt_3_ (2.0 equiv), CH_2_Cl_2_	12
3	1) **15a** (1.1 equiv), NIS (1.1 equiv). 2) **16a** (1.0 equiv), NEt_3_ (2.0 equiv), CH_2_Cl_2_	–
4	1) **15a** (1.1 equiv), Br_2_ (1.1 equiv). 2) **16a** (1.0 equiv), NEt_3_ (2.0 equiv), CH_2_Cl_2_	27
5	1) **15a** (1.0 equiv), Br_2_ (1.1 equiv). 2) **16a** (1.0 equiv), NEt_3_ (1.3 equiv), Et_2_O	42^b^
6	1) **15a** (1.1 equiv), Br_2_ (1.1 equiv). 2) **16a** (1.0 equiv), NEt_3_ (1.1 equiv), THF	–
7	1) **15a** (1.3 equiv), Br_2_ (1.4 equiv). 2) **16a** (1.0 equiv), NEt_3_ (1.3 equiv), Et_2_O	36
8	1) **15a** (1.0 equiv), Br_2_ (1.0 equiv). 2) **16a** (1.0 equiv), NEt_3_ (2.0 equiv), Et_2_O	25
9	1) **15a** (1.0 equiv), Br_2_ (1.0 equiv). 2) **16a** (1.0 equiv), NEt_3_ (2.0 equiv), CCl_4_	75
10	1) **15a** (1.2 equiv), Br_2_ (1.2 equiv), CCl_4_. 2) **16a** (1.0 equiv), NEt_3_ (2.0 equiv), CCl_4_/Et_2_O (4:1)	88

^a^First reaction: Br_2_ (1 M in CCl_4_), 1 h, 0 °C. Second reaction: DMAP (0.1 equiv), 2 h, 0 °C → 25 °C. ^b^65% were obtained once for a small scale reaction.

**Scheme 5:**
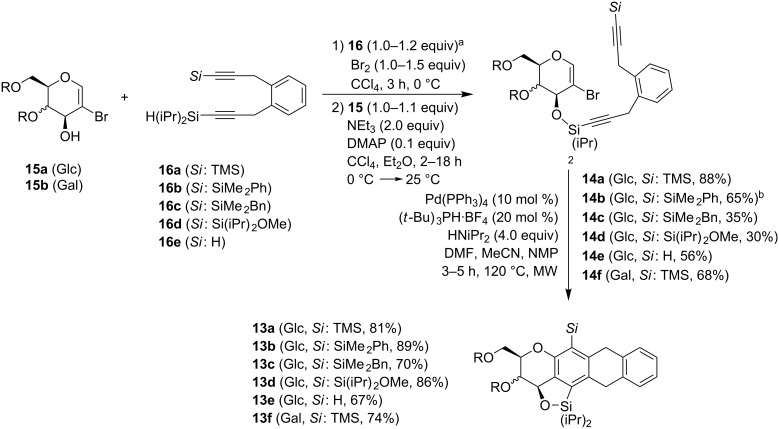
Silyl ether synthesis and domino carbopalladation reaction. *R*,*R* (Glc): isopropylidene. *R*,*R* (Gal): benzylidene. ^a^The respective equivalents of alkynes **16**, glycals **15** and bromine as well as reaction times are given in the Experimental. ^b^The reaction was performed according to entry 5 of Table 1.

With optimized conditions in hand we explored the scope of the silyl ether coupling. Therefore, two different glycals and five different dialkynes were employed. In summary, seven different coupling products **14** were prepared baring alkynes with terminal H, TMS, SiMe_2_Ph, SiMe_2_Bn and Si(iPr)_2_OMe groups (Scheme 5). The best results were obtained with TMS-substituents. Although the SiMe_2_Ph-substituted product was not synthesized under optimal reaction conditions the desired product was formed in high yield. In addition, terminal alkynes were tolerated in the reaction. Contrary, benzyl and methoxide-substituted silanes **14c** and **14d** provided inferior yields.

With several domino precursors in hand we started the investigation of the domino-carbopalladation sequence. To our delight, it was possible to adjust the catalytic system that we developed for the synthesis of chromans and isochromans. Optimal reaction conditions comprise the use of Pd(PPh_3_)_4_ as a palladium source, (*t*-Bu)_3_PH·BF_4_ (Fu’s salt) [38] as an additional electron-rich and sterically encumbered ligand and HN(iPr)_2_ as a base. As solvent a mixture consisting of *N*,*N*-dimethylformamide, acetonitrile and *N*-methylpyrrolidone (8:8:1) was used. The reaction was performed in a sealed vial at 120 °C under microwave irradiation for 3–5 h. The unusual combination of Pd(PPh_3_)_4_ and (*t*-Bu)_3_PH·BF_4_ as an additional ligand proved beneficial for the transformation of long-chained dialkynes. The domino reaction proceeded smoothly and delivered the desired compounds as major products. Scheme 5 illustrates that all attached substituents at the terminal triple bond were tolerated. Even unsubstituted alkyne **14e** and electron-deficient silane **14d** furnished the product in high yields. TMS, SiMe_2_Ph and Si(iPr)_2_OMe-substituted silanes delivered the best results with yields of up to 89%. For the reaction mechanism we assume that the palladium(0) inserts into the C(sp^2^)–Br bond to form a Pd(II) species. A sequence of two carbopalladation reactions form a triene system which is able to cyclize by a Heck-type reaction, a 6π-electrocyclization [39] or a direct CH-activation to the respective anthracycline precursor **13**. The design of the dialkyne provides a simultaneous formation of the linear ring system. All four cycles were annulated in a single step, in which the B and C-ring were formed as a consequence of the reaction cascade.

### Derivatization to anthracycline derivatives

The derivatization of the domino products turned out to be challenging. Utilization of a fluoride source (e.g. tetrabutylammonium fluoride or tetramethylammonium fluoride) and **13a** lead to total decomposition of the starting materials. Application of Tamao–Fleming-like oxidative procedures provided only the mono-oxidized products in trace amounts [40–42]. The oxidation of phenyl-substituted silanes to respective phenols is difficult [43–44]. However, literature precedence revealed that benzyl-substituted silane **13c** or electron-deficient silane **13d** [43,45] should be more promising candidates. But none of these domino products **13b**–**13f** provided better results in a Tamao–Fleming reaction. When oxidizing reaction conditions were applied to silane **13b,** only desilylation of the cyclic silyl ether occurred. Interestingly, benzylsilane **13c** afforded the globally desilylated product in 90% yield under oxidative reaction conditions (KHCO_3_, H_2_O_2_, KF in THF and MeOH), i.e., both silyl ether and terminal silane were cleaved. However, it was not possible to utilize this procedure for compound **13a**. Under these conditions, the silyl ether was cleaved without touching the trimethylsilyl moiety. Another approach of selective silyl ether cleavage was employed by utilization of Cs_2_CO_3_ (5.0 equiv) in methanol at 100 °C.

**Scheme 6:**
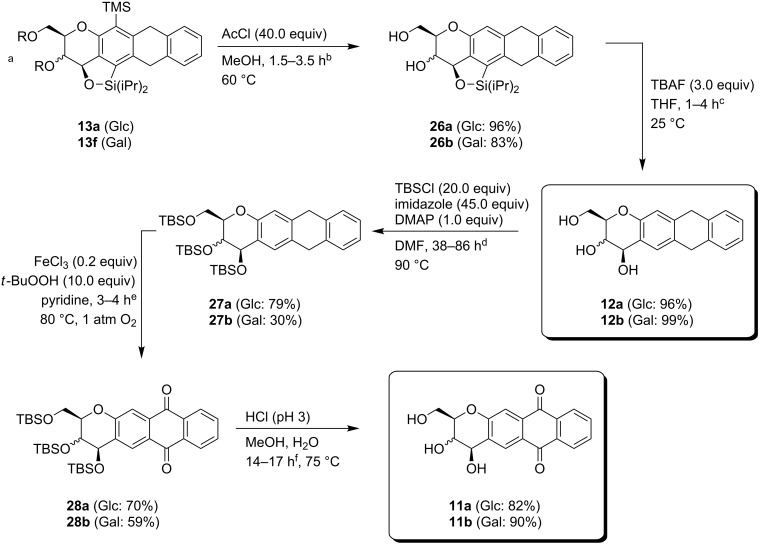
Derivatisation of anthracycline derivatives. ^a^*R*,*R* (Glc): isopropylidene. *R*,*R* (Gal): benzylidene. Reactions times: ^b^1.5 h (Glc), 3.5 h (Gal). ^c^1.0 h (Glc), 4.0 h (Gal). ^d^38 h (Glc), 86 h (Gal). ^e^4.0 h (Glc), 3.0 h (Gal). ^f^14 h (Glc), 17 h (Gal).

Hydrolysis of the respective domino products **13a** and **13f** with in situ formed HCl in methanol furnished the diols **26a** and **26b** under loss of the terminal TMS group [46]. Opening of the silyl ether moiety was accomplished by treatment with TBAF in quantitative yield and gained access to the natural substitution pattern of the carbohydrate backbone. It was not possible to open the silyl ether moiety of **26** by the utilization of Cs_2_CO_3_ in methanol starting from **13** as described before. To install the anthraquinone moiety it was necessary to reprotect the alcohol functionalities. It has proven challenging to install the TBS protecting group at the substrates, particular for the galactose-derived derivatives **12b** which could be obtained in only poor yield [47–49]. For **27a** the FeCl_3_-catalyzed benzylic oxidation proceeded smoothly with yields of up to 70% [50]. A final hydrolysis with hydrochloric acid afforded the desired carbohydrate-based anthracycline derivatives **11** in good yield (Scheme 6).

## Conclusion

In conclusion, we have developed a concise and robust approach to anthracycline aglycone derivatives. Starting materials were bromoglycals and benzene moieties with two propynyl residues. The first key step is the union of these moieties by a silyl ether linkage. In a second key step the tetracyclic anthracycline scaffold is formed by a domino carbopalladation sequence generating both, the B and the C-ring of the system in a single step. Further derivatisation included the cleavage of the silyl ether and two-fold benzylic oxidation to the quinone moiety. We believe that these natural product mimics might be of interest as useful candidates for drug discovery research.

## Supporting Information

File 1 Experimental details and analytical data of all new compounds as well as their ^1^H and ^13^C NMR spectra.
