# Supplementary material for: Flexible synthesis of anthracycline aglycone mimics via domino carbopalladation reactions
Source: Beilstein J Org Chem. 2013 Oct 24;9:2194–201. doi: 10.3762/bjoc.9.258 (PMC3817477; doi:10.3762/bjoc.9.258)

Supporting Information

for

Flexible synthesis of anthracycline aglycone mimics via domino carbopalladation reactions

Markus Leibeling and Daniel B. Werz*

Address: Institut für Organische Chemie, Technische Universität Braunschweig, Hagenring 30, 38106 Braunschweig, Germany.

Email: Prof. Dr. Daniel B. Werz* - d.werz@tu-braunschweig.de

* Corresponding author

Experimental details and analytical data of all new compounds as well as their ^{1}H and ^{13}C NMR spectra

Table of Contents

General experimental S2
Preparative methods S2
Chromatography S2
Instrumental analytics S2
Synthesis and analytical data of the compounds S4
General procedures S4
Analytical data S6
^{1}H and ^{13}C NMR spectra of the compounds S30

General experimental

Preparative methods

All solvents for column chromatography were distilled before use unless otherwise stated. Tetrahydrofuran (THF) and diethyl ether (Et_2O) were distilled from sodium/benzophenone under an argon atmosphere. Dichloromethane (CH_2Cl_2) and toluene were distilled from $CaCl_2$ under an argon atmosphere. All other solvents were used as analytical grade and were stored over suitable molecular sieves (3 Å or 4 Å). Air and moisture sensitive reactions were carried out in oven-dried or flame-dried glassware, septum-capped under atmospheric pressure of argon. Commercially available compounds were used without further purification unless otherwise stated.

Microwave: A *Biotage*® microwave oven with adjustable temperature in the range of 40-250 °C has been used to apply microwave irradiation. Pressures of 20 bar are possible inside the reaction vessel. The power of the magnetron at 2.45 GHz is regulated between 0-400 W. The reaction volumes are available in four different dimensions (0.2–0.5 mL, 0.5–2.0 mL, 2.5–5.0 mL and 10.0–20.0 mL,). The device was run with the software version 2.3 build 6250. Exact reaction conditions are given in the respective procedure.

Chromatography

Thin-layer chromatography (TLC): Analytical TLC was performed on Merck silica gel plates (60 F_{254}, 0.25 mm). The compounds were visualized under UV irradiation (254 nm and 350 nm). As common coloration reagent were used cerium ammonium molybdate in solution [5 g $(NH_4)_4Ce(SO_4)_4$, 30 g $Mo_7(NH_4)_6\cdot 4H_2O$, 30 mL conc. H_2SO_4 and 600 mL H_2O] and elementary iodine for the detection of alkynes.

Colum Chromatography: Preparative purification of the products was achieved by flash column chromatography on Merck silica gel (grade 60, 0.063–0.200 mm, 70–230 mesh ASTM) with elevated pressure. Difficult purifications were accomplished by middle pressure liquid chromatography (MPLC) by *Biotage*® Isolera™ One. As stationary phase *Biotage*® SNAP cadridges KPSIL (10-250 g silica gel, 30 or 50 µm) were used.

Instrumental analytics

NMR spectra: Proton (^1H) and carbon (^{13}C) NMR spectra were recorded on 300, 500 and 600 MHz instruments (Varian Unity-300, Varian Mecury-300, Varian Inova-500 and Varian Inova-600), respectively, using the residual signals from $CHCl_3$: δ = 7.26 ppm and δ = 77.0 ppm; DMSO: δ = 2.54 ppm and δ = 40.5 ppm; acetone: δ = 2.09 ppm and δ = 30.9 ppm and δ = 207.1 ppm and methanol: δ = 4.87 ppm and δ = 49.2 ppm, as internal references for ^1H and ^{13}C chemical shifts, respectively. Assignments of the respective signals were made by the combination of H,H-COSY, HSQC, NOE and HMBC experiments. The

splitting patterns are indicated as s, singlet; d, doublet; t, triplet; q, quartet; s_{br}, broad singlet; m, multiplet and as combination of that kind. Undefined assignments of hydrogen and carbon atoms are marked by * sign.

Mass spectra: ESIMS and HRMS–ESI mass spectrometry was carried out on a FTICR instrument by Finnigan LCQ and on an APEX IV 7T FTICR by Bruker Daltonic. EIMS and HRMS–EI mass spectrometry was performed on an EI-TOF (Accu TOF) by Jeol and a sector instrument (MAT 95) by Finnigan.

IR (ATR) and UV Spectra: IR (ATR) spectra were measured on a conventional ATR spectrometer (FT/IR-4100) by JASCO. UV spectra were measured with a common photometer (V630) by JASCO in acetonitrile or methanol as solvent.

Optical rotation values were measured at 20 °C using a polarimeter by Perkin-Elmer 241 in respective suitable solvents (e.g. chloroform, methanol and dimethyl sulfoxide).

X-ray analysis: For the X-ray crystal structures a single crystal was mounted in an inert oil. The data was collected from cooled crystals at 100 K on a Bruker Smart ApexII with Incoatec micro focus source using monochromatic MoK_{α} radiation, λ = 0.71073 Å. Data reduction was done with SAINT,[1] and an empirical absorption correction with SADABS[2] or TWINABS[3] was applied. The structure was solved by direct methods (SHELXS-97)[4] and refined by full-matrix least-squares methods against F^2 (SHELXL-97).[5] All non-hydrogen atoms were refined with anisotropic displacement parameters. The hydrogen atoms were refined isotropically on calculated positions using a riding model with their U_{iso} values constrained to 1.5 times the U_{eq} of their pivot atoms for terminal sp^3 carbon atoms and 1.2 times for all other carbon atoms.

Literature

1. Bruker, SAINT V7.68A, Bruker AXS Inc., Madison (WI, USA), **2005.**
2. Sheldrick, G. M.; SADABS 2008/2, Göttingen, **2008**.
3. Sheldrick, G. M.; TWINABS 2008/4, Göttingen, **2008**.
4. Sheldrick, G. M. *Acta Crystallogr. Sect. A* **2008**, *64*, 112-122.
5. Flack, H. D. *Acta Crystallogr. Sect. A* **1983**, *39*, 876-881.

Synthesis and analytical data of the compounds

General procedures

Common aqueous workup:

The reaction was stopped by addition of saturated aq. NH_4Cl solution. The aqueous layer was extracted three times with EtOAc. The combined organic layers were washed with saturated aq. NaCl solution, dried over Na_2SO_4 and the solvent was removed by rotary evaporation.

GP1: Appel reaction

The alcohol (1.0 equiv), triphenylphosphine (1.5–2.0 equiv) and imidazole (1.5–2.0 equiv) were dissolved in a mixture of Et_2O and MeCN and cooled to 0 °C. Iodine (1.5–2.0 equiv) was added portionwise over a period of 45 min and the reaction was allowed to stir for further 15 min (monitored by TLC). After completion of the reaction, the mixture was poured into pentane and was immediately purified by silica gel column chromatography (pentane) to afford the desired product.

GP2: Silylacetylene coupling

To a solution of silylacetylene (2.0–3.0 equiv) in THF was added ethyl magnesium bromide (3.0 mol/L in Et_2O) at room temperature. The solution was heated to reflux for 2 h and then cooled to ambient temperature. CuCl (25–40 mol %) and the corresponding iodide (1.0 equiv), dissolved in THF, were successively added to the reaction mixture and heated to 75 °C for 16 h. The reaction was stopped by addition of saturated aq. NH_4Cl solution. The aqueous layer was extracted three times with EtOAc. The combined organic layers were washed with saturated aq. NaCl solution and dried over Na_2SO_4. The solvent was removed by rotary evaporation and the residue was purified by silica gel column chromatography to afford the desired product.

GP3: TBS-cleavage

The protected alcohol (1.0 equiv) was dissolved in MeOH at 0 °C and AcCl was added dropwise. The reaction mixture was stirred for 3 h and then stopped by slow addition of $NaHCO_3$ solution. The aqueous layer was extracted three times with EtOAc. The combined organic layers were washed with saturated aq. NaCl solution and dried over Na_2SO_4. The solvent was removed by rotary evaporation and the residue was purified by silica gel column chromatography to afford the desired product.

GP4: Silyl ether formation

Dialkyne (1.0–1.2 equiv) was dissolved in dry CCl_4 and cooled to 0 °C. A freshly prepared solution of bromine in dry CCl_4 (0.976 mol/L, 1.0–1.2 equiv) was added over a period of 1 h

via syringe pump and stirred for further 2 h at 0 °C. To a solution of glycal (1.0 equiv), NEt_3 (2.0 equiv) and DMAP (10 mol %) in dry CCl_4 and dry Et_2O was added the solution of the alkyne at 0 °C over a period of 30 min via syringe pump. The reaction was stirred for at least 2 h and stopped by the addition of saturated aq. NH_4Cl solution. The aqueous layer was extracted three times with EtOAc. The combined organic layers were washed with saturated aq. NaCl solution and dried over Na_2SO_4. The solvent was removed by rotary evaporation and the residue was purified by silica gel column chromatography to afford the desired product.

GP5: Domino reaction

The alkynylated bromoglycal (1.0 equiv) was dissolved in a mixture of DMF/MeCN/NMP (8:8:1). $Pd(PPh_3)_4$ (10 mol %), *t*-$Bu_3PH{\cdot}BF_4$ (20 mol %) and diisopropylamine (5.0 equiv) were added. The reaction was stirred in a microwave reactor for 3–5 h at 120 °C. The absorption level was set as very high and the prestirring time at 10 s. The reaction was stopped by the addition of saturated aq. NH_4Cl solution. The aqueous layer was extracted three times with EtOAc. The combined organic layers were washed with saturated aq. NaCl solution, dried over Na_2SO_4. The solvent was removed by rotary evaporation and the residue was purified by silica gel column chromatography to afford the desired product.

Analytical data

Compound 20

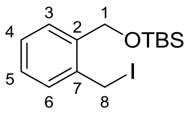

Alcohol **19** (3.34 g, 13.3 mmol, 1.0 equiv), triphenylphosphine (6.95 g, 26.5 mmol, 2.0 equiv), imidazole (1.80 g, 26.5 mmol, 2.0 equiv) and iodine (6.73 g, 26.5 mmol, 2.0 equiv), dissolved in Et_2O (100 mL) and MeCN (25 mL) were brought to reaction according to GP1. Purification by silica gel column chromatography (pentane) afforded 4.44 g (93%) of compound **20** as an orange oil: **R_f**: 0.53 (hexane/EtOAc = 20:1). **^{1}H-NMR** (300 MHz, $CDCl_3$): δ = 0.14 (s, 6 H, Me_{TBS}), 0.96 (s, 9 H, tBu$_{TBS}$), 4.51 (s, 2 H, 1-H), 4.79 (s, 2 H, 8-H), 7.17–7.34 (m, 3 H, 3-H*, 4-H*, 5-H*), 7.38–7.42 (m, 1 H, 6-H*). **^{13}C-NMR** (125 MHz, $CDCl_3$): δ = –5.1 (C-8), 3.3 (Me_{TBS}), 18.4 ($C_{q,TBS}$), 26.0 (tBu$_{TBS}$), 62.5 (C-1), 127.5, 127.6, 128.2, 129.7 (C-3, C-4, C-5, C-6), 135.9, 139.1 (C-2, C-7). **IR** (ATR): $\tilde{v}$ (cm^{-1}) = 3271, 2852, 1918, 1455, 1368, 1185. **UV** (CH_3CN): λ_{max} [nm] (lg ε) = 241 (4.03), 360 (2.77). **MS** (ESI): *m*/*z* (%) = 385.1 (32) $[M+Na]^+$. **$C_{14}H_{23}IOSi$** (362.32), calc.: 385.0455, found.: 385.0453, $[M+Na]^+$ (ESI-HRMS).

Compound 22a

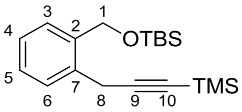

Trimethylsilylacetylene (542 mg, 5.52 mmol, 2.0 equiv) in THF (30 mL), ethyl magnesium bromide (3.0 mol/L in diethyl ether, 1.84 mL, 5.52 mmol, 2.0 equiv), CuCl (69 mg, 0.69 mmol, 25 mol %) and **20** (1.00 g, 2.76 mmol, 1.0 equiv) in THF (10 mL) were brought to reaction according to GP5.2. Purification by silica gel column chromatography (pentane/EtOAc = 100:1) afforded 807 mg (88%) of compound **22a** as a pale yellow oil: **R_f**: 0.53 (hexane/EtOAc = 20:1). **^{1}H-NMR** (300 MHz, $CDCl_3$): δ = 0.10–0.11 (m, 6 H, Me_{TBS}), 0.18–0.20 (m, 9 H, Me_{TMS}), 0.94–0.96 (s, 9 H, tBu$_{TBS}$), 3.64 (s, 2 H, 8-H), 4.78 (s, 2 H, 1-H), 7.24–7.29 (m, 2 H, H_{Ar}), 7.41–7.46 (m, 2 H, H_{Ar}). **^{13}C-NMR** (125 MHz, $CDCl_3$): δ = –5.2 (Me_{TBS}), 0.2 (Me_{TMS}), 18.5 ($C_{q,TBS}$), 23.5 (C-8), 26.0 (tBu$_{TBS}$), 63.0 (C-1), 87.0, 103.7 (C-9, C-10), 126.7, 126.8, 127.2, 128.4 (C-3, C-4, C-5, C-6), 133.5, 138.6 (C-2, C-7). **IR** (ATR): $\tilde{v}$ (cm^{-1}) = 2955, 2856, 2175, 1471, 1249. **UV** (CH_3CN): λ_{max} [nm] (lg ε) = 194 (4.82). **MS** (ESI): *m*/*z* (%) = 355.2 (100) $[M+Na]^+$. **$C_{19}H_{32}OSi_2$** (332.63), calc.: 355.1884, found: 355.1885, $[M+Na]^+$ (ESI-HRMS).

Compound 23a

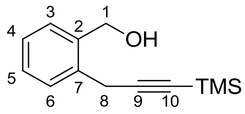

The protected alcohol **22a** (4.80 g, 14.4 mmol, 1.0 equiv), dissolved in MeOH (45 mL), and AcCl (6.0 mL) were brought to reaction according to GP5.3. Purification by silica gel column chromatography (pentane/EtOAc = 10:1) afforded 2.71 g (86%) of compound **23a** as a slightly yellow oil: **R_f**: 0.21 (hexane/EtOAc = 5:1). **^{1}H-NMR** (300 MHz, $CDCl_3$): δ = 0.17 (s, 9 H, Me_{TMS}), 3.71 (s, 2 H, 8-H), 4.74 (s, 2 H, 1-H), 7.25–7.31 (m, 2 H, H_{Ar}), 7.36–7.45 (m, 2 H, H_{Ar}). **^{13}C-NMR** (125 MHz, $CDCl_3$): δ = –0.03 (C-Me_{TMS}), 23.7 (C-8), 63.3 (C-1), 87.3, 104.5 (C-9, C-10), 127.3, 128.3, 128.7, 129.1 (C-3, C-4, C-5, C-6), 134.8, 138.3 (C-2, C-7). **IR** (ATR): $\tilde{\nu}$ (cm^{-1}) = 3321, 2958, 2174, 1455, 1412, 1248, 1016. **UV** (CH_3CN): λ_{max} [nm] (lg ε) = 194 (4.74). **MS** (EI, 70 eV): *m*/*z* (%) = 217.1 (5) $[M-H]^+$. **$C_{13}H_{18}OSi$** (218.37), calc.: 217.1049, found: 217.1058, $[M-H]^+$ (EI-HRMS).

Compound 24a

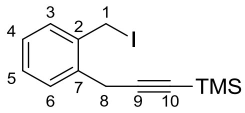

Alcohol **23a** (2.67 g, 12.2 mmol, 1.0 equiv), triphenylphosphine (4.81 g, 18.3 mmol, 1.5 equiv), imidazole (1.25 g, 18.3 mmol, 1.5 equiv) and iodine (4.66 g, 18.3 mmol, 1.5 equiv), dissolved in Et_2O (100 mL) and MeCN (25 mL) were brought to reaction according to GP1. Purification by silica gel column chromatography (pentane) afforded 3.96 g (99%) of compound **24a** as an orange oil: **R_f**: 0.68 (hexane/EtOAc = 10:1). **^{1}H-NMR** (300 MHz, $CDCl_3$): δ = 0.17 (s, 9 H, Me_{TMS}), 3.68 (s, 2 H, 8-H), 4.48 (s, 2 H, 1-H), 7.16–7.33 (m, 3 H, H_{Ar}), 7.42–7.47 (m, 1 H, H_{Ar}). **^{13}C-NMR** (125 MHz, $CDCl_3$): δ = –0.1 (Me_{TMS}), 3.5 (C-1), 23.5 (C-8), 87.9, 102.9 (C-9, C-10), 127.5, 128.7, 129.4, 129.8 (C-3, C-4, C-5, C-6), 134.8, 136.5 (C-2, C-7). **IR** (ATR): $\tilde{\nu}$ (cm^{-1}) = 2957, 2174, 1488, 1454, 1247, 1152, 837. **UV** (CH_3CN): λ_{max} [nm] (lg ε) = 240 (3.91). **MS** (ESI): *m*/*z* (%) = 279.1 (6) $[M-TMS+Na]^+$. **$C_{13}H_{17}ISi$** (328.26).

Compound 16a

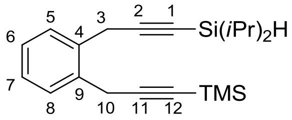

Silylacetylene **25** (1.50 g, 10.7 mmol, 3.0 equiv) in THF (50 mL), ethyl magnesium bromide (3.0 mol/L in diethyl ether, 3.55 mL, 10.7 mmol, 3.0 equiv), CuCl (141 mg, 1.42 mmol,

40 mol %) and **24a** (1.17 g, 3.55 mmol, 1.0 equiv) in THF (15 mL) were brought to reaction according to GP5.2. Purification by silica gel column chromatography (pentane/CH_2Cl_2 = 150:1) afforded 1.15 g (95%) of compound **16a** as a yellow oil: **R_f**: 0.32 (hexane/CH_2Cl_2 = 10:1). **^{1}H-NMR** (300 MHz, $CDCl_3$): δ = 0.17–0.18 (m, 9 H, Me_{TMS}), 1.04–1.10 (m, 14 H, *i*Pr-H), 3.64 (s, 2 H, 3-H*), 3.69 (s, 2 H, 10-H*), 3.72 (s, 1 H, Si*H*), 7.23–7.29 (m, 2 H, H_{Ar}), 7.42–7.53 (m, 2 H, H_{Ar}). **^{13}C-NMR** (125 MHz, $CDCl_3$): δ = 0.0 (Me_{TMS}), 10.9, 18.3, 18.5 (C_{iPr}), 23.9, 24.0 (C-3, C-10), 81.1, 87.4 (C-2, C-11), 103.3, 106.1 (C-1, C-12), 127.2, 127.2, 128.6, 128.7 (C-5, C-6, C-7, C-8), 134.1, 134.1 (C-4, C-9). **IR** (ATR): $\tilde{\nu}$ (cm^{-1}) = 2942, 2863, 2174, 2116, 1455, 1249. **UV** (CH_3CN): λ_{max} [nm] (lg ε) = 260 (2.45), 193 (4.72). **MS** (ESI): *m*/*z* (%) = 363.3 (33) $[M+Na]^+$. **$C_{21}H_{32}Si_2$** (340.65), calc.: 363.1935, found: 363.1936, $[M+Na]^+$ (ESI-HRMS).

Compound 14a

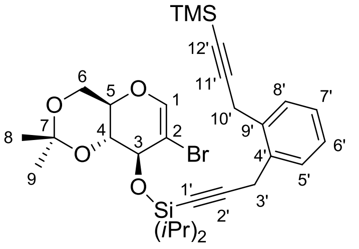

Dialkyne **16a** (582 mg, 1.71 mmol, 1.2 equiv) was dissolved in dry CCl_4 (20.0 mL) and cooled to 0 °C. A freshly prepared solution of bromine in dry CCl_4 (0.976 mol/L, 1.75 mL, 1.71 mmol, 1.2 equiv) was added over a period of 1 h via syringe pump and stirred for further 2 h at 0 °C. The resulting solution was added to a mixture of glucal **15a** (378 mg, 1.42 mmol, 1.0 equiv), NEt_3 (395 µL, 2.85 mmol, 2.0 equiv) and DMAP (18 mg, 0.142 mmol, 10 mol %) in dry CCl_4 (20.0 mL) and dry Et_2O (2.5 mL) at 0 °C over a period of 30 min via syringe pump. The reaction was stirred over night and stopped by the addition of saturated aq. NH_4Cl solution. The aqueous layer was extracted three times with CH_2Cl_2. The combined organic layers were washed with saturated aq. NaCl solution and dried over Na_2SO_4. The solvent was removed by rotary evaporation and the residue was purified by silica gel column chromatography (pentane/EtOAc = 100:1) to afford 754 mg (88%) of compound **14a** as a colorless oil: **R_f**: 0.62 (hexane/EtOAc = 6:1). $[\boldsymbol{\alpha}]_{\mathbf{D}}^{\mathbf{20}}$ = +52.7° (c = 0.33, $CHCl_3$). **^{1}H-NMR** (300 MHz, $CDCl_3$): δ = 0.18 (s, 9 H, Me_{TMS}), 1.07–1.14 (m, 14 H, *i*Pr), 1.38 (s, 3 H, 8-H), 1.48 (s, 3 H, 9-H), 3.65 (s, 2 H, 3'-H), 3.71 (s, 2 H, 10'-H), 3.75–3.83 (m, 2 H, 4-H*, 6-H_a), 3.89–3.97 (m, 2 H, 5-H*, 6-H_b), 4.57 (dd, *J* = 1.4, 7.1 Hz, 1 H, 3-H), 6.56 (d, *J* = 1.4 Hz, 1 H, 1-H), 7.24–7.31 (m, 2 H, H_{Ar}), 7.41–7.47 (m, 1 H, H_{Ar}), 7.55–7.59 (m, 1 H, H_{Ar}). **^{13}C-NMR** (125 MHz, $CDCl_3$): δ = 0.1 (Me_{TMS}), 13.3, 14.0, 17.2, 17.3, 17.6, 17.7 (C_{iPr}), 18.7, 28.9 (C-8, C-9), 23.9, 24.0 (C-3', C-10'), 61.3 (C-6), 70.3, 71.6, 73.2 (C-3, C-4, C-5), 82.8, 87.5, 103.3, 106.1 (C-1', C-2', C-11', C-12'), 99.6 (C-7), 103.7 (C-2), 127.2, 127.2, 128.6, 128.7 (C-5', C-6', C-7', C-8'), 134.0,

134.1 (C-4', C-9'), 143.6 (C-1). **IR** (ATR): ṽ (cm^{-1}) = 2944, 2865, 2173, 1631, 1462, 1249, 1171. **UV** (CH_3CN): λ_{max} [nm] (lg ε) = 193 (4.69). **MS** (ESI): *m/z* (%) = 627.2 (100) $[M+Na]^+$. **$C_{30}H_{43}O_4Si_2Br$** (603.74), calc.: 627.1757, found: 627.1765, $[M+Na]^+$ (ESI-HRMS).

Compound 13a

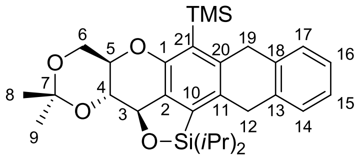

The alkynylated bromoglycal **14a** (300 mg, 0.497 mmol, 1.0 equiv), $Pd(PPh_3)_4$ (58 mg, 0.050 mmol, 10 mol %), *t*-$Bu_3PH·BF_4$ (29 mg, 0.099 mmol, 20 mol %) and diisopropylamine (352 µL, 2.48 mmol, 5.0 equiv), dissolved in DMF/MeCN/NMP (9.0 mL, 9.0 mL, 1.1 mL) were brought to reaction according to GP5. Purification by silica gel column chromatography (pentane/EtOAc = 30:1) afforded 210 mg (81%) of compound **13a** as a white solid: **R_f**: 0.41 (hexane/EtOAc = 10:1). $[\alpha]_D^{20}$ = +27.2° (c = 0.16, $CHCl_3$). **^{1}H-NMR** (300 MHz, $CDCl_3$): δ = 0.41 (s, 9 H, Me_{TMS}), 0.93 (d, *J* = 7.5 Hz, 3 H, $CH_{3,iPr}$), 0.99 (d, *J* = 7.4 Hz, 3 H, $CH_{3,iPr}$), 1.13 (d, *J* = 7.3 Hz, 3 H, $CH_{3,iPr}$), 1.20 (d, *J* = 7.5 Hz, 3 H, $CH_{3,iPr}$), 1.23–1.47 (m, 2 H, CH_{iPr}), 1.53 (s, 3 H, 8-H), 1.55 (s, 3 H, 9-H), 3.71–4.16 (m, 8 H, 4-H, 5-H, 6-H, 12-H, 19-H), 5.09 (d, *J* = 9.1 Hz, 1 H, 3-H), 7.15–7.31 (m, 4 H, 14-H, 15-H, 16-H, 17-H). **^{13}C-NMR** (125 MHz, $CDCl_3$): δ = 2.7 (Me_{TMS}), 12.9, 13.8, 17.2, 17.4, 17.4, 17.7 (C_{iPr}), 19.1, 29.1 (C-8, C-9), 37.3, 39.2 (C-12, C-19), 62.3 (C-6), 70.2, 72.5, 78.1 (C-3, C-4, C-5), 100.0 (C-7), 123.8 (C-21), 126.0, 126.1, 126.3, 126.8 (C-14, C-15, C-16, C-17), 130.1, 133.5, 135.2, 137.6, 137.6, 144.6 (C-2, C-10, C-11, C-13, C-18, C-20), 154.1 (C-1). **IR** (ATR): ṽ (cm^{-1}) = 2941, 2862, 1538, 1459, 1380, 1246, 1091. **UV** (CH_3CN): λ_{max} [nm] (lg ε) = 306 (3.70), 300 (3.68), 263 (3.41), 212 (4.62). **MS** (ESI): *m/z* (%) = 545.3 (100) $[M+Na]^+$. **$C_{30}H_{42}O_4Si_2$** (522.82), calc.: 545.2514, found: 545.2522, $[M+Na]^+$ (ESI-HRMS).

Compound 26a

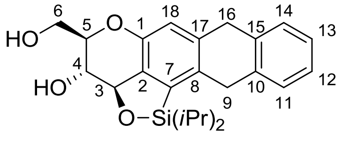

To a solution of **13a** (130 mg, 0.249 mmol, 1.0 equiv) in MeOH (10 mL) was added dropwise AcCl (1.0 mL) at 0 °C. The reaction mixture was stirred at 60 °C for 2 h and then stopped by slow addition of saturated aq. $NaHCO_3$ solution. The aqueous layer was extracted three times with EtOAc. The combined organic layers were washed with saturated aq. NaCl solution and dried over Na_2SO_4. The solvent was removed by rotary evaporation and the residue was purified by silica gel column chromatography to afford 98 mg (96%) of

compound **26a** as a white solid: **R_f**: 0.30 (CH_2Cl_2/MeOH = 10:1). $[\alpha]_D^{20}$ = +38.7° (c = 0.15, $CHCl_3$). **^{1}H-NMR** (300 MHz, $CDCl_3$): δ = 0.86 (d, *J* = 7.4 Hz, 3 H, Me_{iPr}), 1.01 (d, *J* = 7.4 Hz, 3 H, Me_{iPr}), 1.14 (d, *J* = 7.3 Hz, 3 H, Me_{iPr}), 1.19 (d, *J* = 7.5 Hz, 3 H, Me_{iPr}), 1.20–1.34 (m, 1 H, CH_{iPr}), 1.38–1.50 (m, 1 H, CH_{iPr}), 3.72–3.82 (m, 2 H, 9-H_a, 4-H*), 3.83–3.96 (m, 3 H, 9-H_b, 16-H), 3.95–4.08 (m, 2 H, 6-H), 4.14 (dt, *J* = 9.6, 3.7 Hz, 1 H, 5-H*), 5.05 (d, *J* = 9.1 Hz, 1 H, 3-H), 6.79 (s, 1 H, 18-H), 7.16–7.31 (m, 4 H, 11-H, 12-H, 13-H, 14-H). **^{13}C-NMR** (125 MHz, $CDCl_3$): δ = 12.7, 13.8, 17.1, 17.3, 17.3, 17.6 (C_{iPr}), 36.6, 38.0 (C-9, C-16), 62.6 (C-6), 70.5, 78.9 (C-4, C-5), 80.2 (C-3) 115.0 (C-18), 126.2, 126.2, 126.9, 127.3 (C-11, C-12, C-13, C-14), 130.8, 132.8, 132.9, 136.6, 136.8, 138.6 (C-2, C-7, C-8, C-10, C-15, C-17), 149.4 (C-1). **IR** (ATR): $\tilde{v}$ (cm^{-1}) = 3377, 2941, 2863, 2360, 1586, 1447, 1261. **UV** (CH_3CN): λ_{max} [nm] (lg ε) = 260 (4.23). **MS** (ESI): *m*/*z* (%) = 433.2 (62) $[M+Na]^+$. **$C_{24}H_{30}O_4Si$** (410.58), calc.: 433.1806, found: 433.1802, $[M+Na]^+$ (ESI-HRMS).

Compound 12a

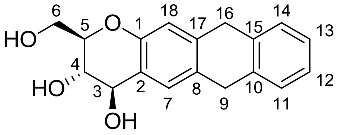

To a solution of **26a** (20 mg, 0.049 mmol, 1.0 equiv) in THF (0.1 mL) was added TBAF (1.0 mol/L in THF, 146 µL, 0.146 mmol, 3.0 equiv). The mixture was stirred for 70 min and directly poured on a packed silica gel column (CH_2Cl_2/MeOH = 10:1). 14 mg (96%) of compound **12a** were obtained as a yellow solid after chromatographic purification: **R_f**: 0.23 (CH_2Cl_2/MeOH = 10:1). $[\alpha]_D^{20}$ = +62.7° (c = 0.11, DMSO). **^{1}H-NMR** (300 MHz, DMSO-d_6): δ = 3.44–3.55 (m, 1 H, 4-H), 3.59–3.71 (m, 1 H, 6-H_a), 3.73–3.83 (m, 2 H, 5-H, 6-H_b), 3.80 (s, 4 H, 9-H, 16-H), 4.40 (t, *J* = 7.1 Hz, 1 H, 3-H), 4.69 (t, *J* = 5.7 Hz, 1 H, OH_{C-6}), 5.28 (d, *J* = 5.2 Hz, 1 H, OH_{C-4}), 5.50 (d, *J* = 6.3 Hz, 1 H, OH_{C-3}), 6.69 (s, 1 H, 18-H), 7.11–7.18 (m, 2 H, 12-H, 13-H), 7.22–7.32 (m, 2 H, 11-H, 14-H), 7.28 (s, 1 H, 7-H). **^{13}C-NMR** (125 MHz, $CDCl_3$): δ = 34.5, 36.0 (C-9, C-16), 60.7 (C-6), 68.5 (C-4), 70.1 (C-3), 79.7 (C-5), 113.8 (C-18), 123.3 (C-2), 125.7, 125.8, 126.5, 127.0 (C-11, C-12, C-13, C-14), 127.0 (C-7), 128.1 (C-17), 136.4, 136.6 (C-10, C-15), 137.0 (C-8), 151.5 (C-1). **IR** (ATR): $\tilde{v}$ (cm^{-1}) = 3319, 2879, 1494, 1429, 1255, 1049. **UV** (CH_3CN): λ_{max} [nm] (lg ε) = 261 (3.67), 283 (3.55). **MS** (ESI): *m*/*z* (%) = 321.1 (100) $[M+Na]^+$. **$C_{18}H_{18}O_4$** (298.33), calc.: 321.1097, found: 321.1097, $[M+Na]^+$ (ESI-HRMS).

Compound 27a

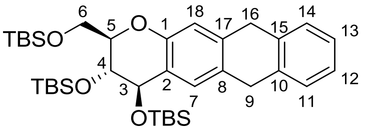

Compound **12a** (40 mg, 0.136 mmol, 1.0 equiv), TBSCl (309 mg, 2.05 mmol, 15.0 equiv), imidazole (418 mg, 6.13 mmol, 45.0 equiv) and DMAP (17 mg, 0.136 mmol, 1.0 equiv) were dissolved in DMF (8.0 mL). The reaction mixture was heated in a sealed vial at 90 °C for 24 h. Then additional TBSCl (100 mg, 0.681 mmol, 20 mol %) was added. The reaction was heated at 90 °C for further 14 h and stopped by the addition of saturated aq. NH_4Cl solution. The aqueous layer was extracted three times with EtOAc. The combined organic layers were washed three times with saturated aq. NaCl solution and dried over Na_2SO_4. The solvent was removed by rotary evaporation and the residue was purified by silica gel column chromatography (pentane/EtOAc = 100:1) to afford 69 mg (79%) of compound **27a** as a colorless oil: **R$_f$**: 0.49 (hexane/EtOAc = 10:1). $[\alpha]_D^{20}$ = +45.0° (c = 0.08, $CHCl_3$). **^{1}H-NMR** (300 MHz, $CDCl_3$): δ = 0.00 (s, 3 H, Me_{TBS}), 0.05 (s, 3 H, Me_{TBS}), 0.08 (s, 3 H, Me_{TBS}), 0.09 (s, 3 H, Me_{TBS}), 0.16 (s, 3 H, Me_{TBS}), 0.19 (s, 3 H, Me_{TBS}), 0.81 (s, 9 H, tBu$_{TBS}$), 0.88 (s, 9 H, tBu$_{TBS}$), 0.89 (s, 9 H, tBu$_{TBS}$), 3.81–3.93 (m, 6 H, 4-H*, 6-H$_a$, 9-H, 16-H), 4.04 (dd, J = 2.4, 0.7 Hz, 1 H, 6-H$_b$*), 4.23–4.29 (m, 1 H, 5-H*), 4.40–4.42 (m, 1 H, 3-H*), 6.80 (s, 1 H, 18-H), 7.02 (s, 1 H, 7-H), 7.17–7.23 (m, 2 H, H$_{Ar}$), 7.25–7.31 (m, 2 H, H$_{Ar}$).**^{13}C-NMR** (125 MHz, $CDCl_3$): δ = –5.3, –5.2, –4.6, –4.6, –4.2, –4.2 (Me_{TBS}), 17.9, 18.0, 18.3 ($C_{q,TBS}$), 25.7, 25.7, 25.9 (tBu$_{TBS}$), 35.2, 36.0 (C-9, C-16), 62.6 (C-6), 68.7, 69.5, 80.7 (C-3, C-4, C-5, C-), 115.5 (C-18), 119.9, 127.9, 136.6, 137.1, 137.5 (C-2, C-8, C-10, C-15, C-17), 125.9, 125.9, 127.3, 1274, 129.3 (C-7, C-11, C-12, C-13, C-14), 150.3 (C-1). **IR** (ATR): $\tilde{\nu}$ (cm^{-1}) = 2928, 2856, 2360, 1471, 1251. **UV** (CH_3CN): λ_{max} [nm] (lg ε) = 261 (4.35). **MS** (ESI): m/z (%) = 663.4 (77) $[M+Na]^+$. **$C_{36}H_{60}O_4Si_3$** (641.12), calc.: 664.3722, found: 664.3694, $[M+Na]^+$ (ESI-HRMS).

Compound 28a

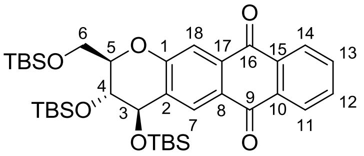

Compound **27a** (64 mg, 0.100 mmol, 1.0 equiv), $FeCl_3 \cdot 6H_2O$ (6 mg, 0.020 mmol, 20 mol %) and t-BuOOH (5.5 mol/L in decane, 182 µL, 0.998 mmol, 10.0 equiv) were dissolved in dry pyridine (4.0 mL). The reaction mixture was aerated with molecular oxygen and heated in a sealed vial at 80 °C for 4 h. After common aqueous workup (general procedures) the residue was purified by silica gel column chromatography (pentane/EtOAc = 25:1) to afford 47 mg (70%) of compound **28a** as a yellow solid: **R$_f$**: 0.43 (hexane/EtOAc = 10:1). $[\alpha]_D^{20}$ = -11.4° (c = 0.14, $CHCl_3$). **^{1}H-NMR** (300 MHz, $CDCl_3$): δ = 0.01 (s, 3 H, Me_{TBS}), 0.06 (s, 3 H, Me_{TBS}), 0.08 (s, 3 H, Me_{TBS}), 0.11 (s, 3 H, Me_{TBS}), 0.19 (s, 3 H, Me_{TBS}), 0.23 (s, 3 H, Me_{TBS}), 0.78 (s, 9 H, tBu$_{TBS}$), 0.87 (s, 9 H, tBu$_{TBS}$), 0.89 (s, 9 H, tBu$_{TBS}$), 3.89 (dd, J = 5.3, 11.3 Hz, 1 H, 6-H$_a$), 3.97 (dd, J = 7.4, 11.3 Hz, 1 H, 6-H$_b$), 4.14 (dd, J = 1.8, 3.2 Hz, 1 H, 4-H*), 4.40–4.46 (m,

1 H, 5-H), 4.55 (dd, *J* = 1.8, 3.2 Hz, 1 H, 3-H*), 7.72 (s, 1 H, 18-H), 7.74–7.79 (m, 2 H, 11-H, 14-H), 8.15 (s, 1 H, 7-H), 8.26–8.31 (m, 2 H, 12-H, 13-H). **^{13}C-NMR** (125 MHz, $CDCl_3$): δ = –5.3, –5.2, –4.7, –4.6, –4.3 (Me_{TBS}), 17.9, 18.0, 18.3 ($C_{q,TBS}$), 25.6, 25.7, 25.9 (tBu_{TBS}), 62.6 (C-6), 68.4, 68.4, 82.4 (C-3, C-4, C-5), 115.6 (C-18), 126.4, 128.5, 133.8, 133.9, 135.1 (C-2, C-8, C-10, C-15, C-17), 127.0, 127.1, 131.4, 133.6, 134.0 (C-7, C-11, C-12, C-13, C-14), 157.9 (C-1), 182.1, 183.0 (C-9, C-16). **IR** (ATR): $\tilde{\nu}$ (cm^{-1}) = 2928, 2856, 1674, 1462, 1289. **UV** (CH_3CN): λ_{max} [nm] (lg ε) = 247 (4.32), 275 (4.53). MS (ESI): *m*/*z* (%) = 691.6 (82) $[M+Na]^+$. **$C_{36}H_{56}O_6Si_3$** (669.08), calc.: 692.3308, found: 692.3288, $[M+Na]^+$ (ESI-HRMS).

Compound 11a

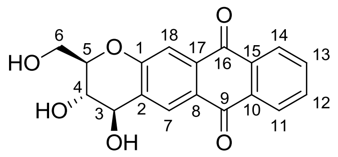

To a solution of **28a** (43 mg, 0.098 mmol, 1.0 equiv) in MeOH (8.0 mL) and water (0.8 mL) was added HCl (0.3 mol/L, 0.8 mL). The mixture was stirred for 14 h at 75 °C and stopped by the addition of saturated aq. $NaHCO_3$ solution. The aqueous layer was extracted three times with EtOAc. The combined organic layers were washed with saturated aq. NaCl solution and dried over Na_2SO_4. The solvent was removed by rotary evaporation and the residue was filtered over a small plug of celite (CH_2Cl_2: 50 mL and CH_2Cl_2/MeOH = 5:1: 250 mL). The product-containing fractions were collected, the solvent was removed by rotary evaporation and the residue was washed with water (250 mL). 26 mg (82%) of compound **11a** were obtained as a shiny yellow solid: **yield**: 82% (99% for small scale reactions). **R_f**: 0.34 (CH_2Cl_2/MeOH = 7:1). $[\boldsymbol{\alpha}]_{\mathbf{D}}^{\mathbf{20}}$ = +74.6° (c = 0.13, DMSO). **^{1}H-NMR** (600 MHz, DMSO-*d*$_6$): δ = 3.65 (t, *J* = 9.1 Hz, 1 H, 4-H), 3.77 (dt, *J* = 12.0, 4.9 Hz, 1 H, 6-H_a), 3.86 (dd, *J* = 12.0, 2.4 Hz, 1 H, 6-H_b), 4.10 (ddd, *J* = 9.1, 4.9, 2.4 Hz, 5 H), 4.60 (d, *J* = 8.3. 3.6 Hz, 1 H, 3-H), 4.86 (t, *J* = 5.9 Hz, 1 H, $OH_{C\text{-}6}$), 5.66 (s_{br}, 1 H, $OH_{C\text{-}4}$), 6.10 (d, *J* = 5.7 Hz, 1 H, $OH_{C\text{-}3}$), 7.41 (s, 1 H, 18-H), 7.86–7.94 (m, 2 H, 12-H, 13-H), 8.16–8.21 (m, 2 H, 11-H, 14-H), 8.30 (s, 1 H, 7-H). **^{13}C-NMR** (125 MHz, DMSO-*d*$_6$): δ = 60.2 (C-6), 66.8 (C-4), 69.4 (C-3), 80.9 (C-5), 112.9 (C-18), 125.9, 126.6, 126.6, 134,5 (C-11, C-12, C-13, C-14) 128.1 (C-7), 132.7, 133.1, 133,2, 133.8, 134,0, (C-2, C-8, C-10, C-15, C-17), 158,5(C-1), 181,1, 182,0 (C-9, C-16). **IR** (ATR): $\tilde{\nu}$ (cm^{-1}) = 3498, 3398, 3323, 2953, 1672, 1584, 1364. **UV** (MeOH): λ_{max} [nm] (lg ε) = 275 (3.92). **MS** (ESI): *m*/*z* (%) = 325.2 (100) $[M-H]^-$. **$C_{18}H_{14}O_6$** (326.3), calc.: 325.0718, found: 325.0715, $[M-H]^-$ (ESI-HRMS).

Compound 23b

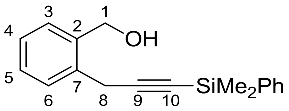

To a solution of dimethylphenylsilylacetylene (3.32 g, 20.7 mmol, 1.5 equiv) in THF (120 mL) was added ethyl magnesium bromide (3.0 mol/L in diethyl ether, 6.90 mL, 20.7 mmol, 1.5 equiv) at room temperature. The solution was heated to reflux for 2 h and then cooled to ambient temperature. CuCl (342 mg, 3.45 mmol, 25 mol %) and **20** (5.00 g, 13.8 mmol, 1.0 equiv) were added to the reaction mixture and heated to 75 °C for 16 h. The reaction was stopped by addition of saturated aq. NH_4Cl solution. The aqueous layer was extracted three times with EtOAc. The combined organic layers were washed with saturated aq. NaCl solution and dried over Na_2SO_4. The solvent was removed by rotary evaporation.

The crude product was dissolved in MeOH (60 mL) at 0 °C and AcCl (6.0 mL) was added dropwise. The reaction mixture was stirred for 3 h and then stopped by slow addition of $NaHCO_3$ solution. The aqueous layer was extracted three times with EtOAc. The combined organic layers were washed with saturated aq. NaCl solution and dried over Na_2SO_4. The solvent was removed by rotary evaporation and the residue was purified by silica gel column chromatography (pentane/EtOAc = 7:1) to afford 2.83 g (73% over two steps) of compound **23b** as a yellow oil: **R_f**: 0.14 (hexane/EtOAc = 5:1). **^{1}H-NMR** (300 MHz, $CDCl_3$): δ = 0.44 (s, 6 H, Me_{DMPS}), 1.88 (s_{br}, 1 H, O*H*), 3.78 (s, 2 H, 8-H), 4.74 (s, 2 H, 1-H), 7.25–7.32 (m, 2 H, H_{Ar}), 7.36–7.41 (m, 4 H, H_{Ar}), 7.47–7.52 (m, 1 H, H_{Ar}), 7.62–7.66 (m, 2 H, H_{Ar}). **^{13}C-NMR** (125 MHz, $CDCl_3$): δ = –0.7 (Me_{DMPS}), 23.8 (C-8), 63.2 (C-1), 85.4, 106.1 (C-9, C-10), 127.2, 127.7, 128.2, 128.4, 129.0, 129.3, 133.5 (C-3, C-4, C-5, C-6, $C_{Ph,DMPS}$), 134.4, 137.0, 138.1 (C-2, C-7, $C_{q,DMPS}$). **IR** (ATR): $\tilde{\nu}$ (cm^{-1}) = 3363, 2957, 2893, 1604, 1488, 1427, 1249, 1113. **UV** (CH_3CN): λ_{max} [nm] (lg ε) = 193 (4.89), 253 (3.03). **MS** (ESI): *m*/*z* (%) = 303.1 (100) $[M+Na]^+$. **$C_{18}H_{20}OSi$** (280.44), calc.: 303.1176, found: 303.1163, $[M+Na]^+$ (ESI-HRMS).

Compound 24b

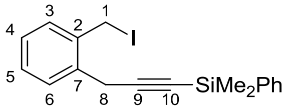

Alcohol **23b** (1.26 g, 4.48 mmol, 1.0 equiv), triphenylphosphine (1.76 g, 6.72 mmol, 2.0 equiv), imidazole (0.46 g, 6.72 mmol, 2.0 equiv) and iodine (1.71 g, 6.72 mmol, 2.0 equiv), dissolved in Et_2O (40 mL) and MeCN (10 mL) were brought to reaction according to GP1. Purification by silica gel column chromatography (pentane) afforded 1.71 g (98%) of compound **24b** as an orange oil: **R_f**: 0.53 (hexane/EtOAc = 10:1). **^{1}H-NMR** (300 MHz, $CDCl_3$): δ = 0.44 (s, 6 H, Me_{DMPS}), 3.77 (s, 2 H, 8-H), 4.50 (s, 2 H, 1-H), 7.18–7.35 (m, 3 H,

H_{Ar}), 7.35–7.41 (m, 3 H, H_{Ar}), 7.47–7.51 (m, 1 H, H_{Ar}), 7.61–7.67 (m, 2 H, H_{Ar}). **^{13}C-NMR** (125 MHz, $CDCl_3$): δ = 0.6 (C-1), 3.6 (Me_{DMPS}), 23.7 (C-8), 85.9, 104.8 (C-9, C-10), 127.5, 127.8, 128.7, 129.3, 129.4, 129.8, 133.6 (C-3, C-4, C-5, C-6, $C_{Ph,DMPS}$), 134.6, 136.4, 137.1 (C-2, C-7, $C_{q,DMPS}$). **IR** (ATR): ṽ (cm^{-1}) = 3551, 2956, 1715, 1427, 1247, 1112. **UV** (CH_3CN): λ_{max} [nm] (lg ε) = 241 (3.90). **MS** (ESI): *m*/*z* (%) = 413 (57) $[M+Na]^+$. **$C_{18}H_{19}ISi$** (390.33).

Compound 16b

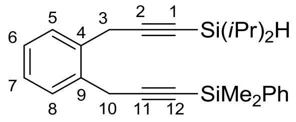

Silylacetylene **25** (70% in THF, 1.47 g, 7.33 mmol, 2.0 equiv) in THF (40 mL), ethyl magnesium bromide (3.0 mol/L in diethyl ether, 2.50 mL, 7.33 mmol, 2.0 equiv), CuCl (91 mg, 0.916 mmol, 25 mol %) and **24b** (1.43 g, 3.66 mmol, 1.0 equiv) in THF (20 mL) were brought to reaction according to GP5.2. Purification by silica gel column chromatography (pentane/CH_2Cl_2 = 50:1 → 20:1) afforded 1.08 g (73%) of compound **16b** as a yellow oil: **R_f**: 0.26 (hexane/CH_2Cl_2 = 10:1). **^{1}H-NMR** (300 MHz, $CDCl_3$): δ = 0.42–0.44 (m, 6 H, Me_{DMPS}), 1.05–1.12 (m, 14 H, *i*Pr), 3.71 (s, 2 H, 3-H), 3.72 (s, 2 H, 10-H), 3.73 (s_{br}, 1 H, Si*H*), 7.25–7.30 (m, 2 H, H_{Ar}), 7.36–7.40 (m, 3 H, H_{Ar}), 7.46–7.53 (m, 2, H_{Ar}), 7,62-7,67 (m, 2 H, H_{Ar}). **^{13}C-NMR** (125 MHz, $CDCl_3$): δ = –0.6 (Me_{DMPS}), 11.0 (CH_{iPr}), 18.4, 18.6 ($CH_{3,iPr}$), 24.1, 24.1 (C-3, C-10), 81.2, 85.4, 105.2, 106.0 (C-1, C-2, C-11, C-12), 127.1, 127.2, 127.8, 128.6, 128.6, 129.2, 133.6 (C-5, C-6, C-7, C-8, $C_{Ph,DMPS}$), 133.8, 134.0, 137.2 (C-4, C-9, $C_{q,DMPS}$). **IR** (ATR): ṽ (cm^{-1}) = 2941, 2863, 2173, 2115, 1489, 1428, 1248, 1113. **UV** (CH_3CN): λ_{max} [nm] (lg ε) = 244 (2.98), 259 (2.95). **MS** (ESI): *m*/*z* (%) = 425.2 (100) $[M+Na]^+$. **$C_{26}H_{34}Si_2$** (402.72), calc.: 425.2091, found: 425.2083, $[M+Na]^+$ (ESI-HRMS).

Compound 14b

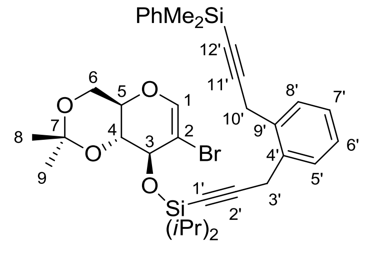

Dialkyne **16b** (100 mg, 0.245 mmol, 1.0 equiv) was dissolved in dry Et_2O (10 mL) and cooled to 0 °C. A freshly prepared solution of bromine in dry Et_2O (0.976 mol/L, 267 µL, 0.261 mmol, 1.05 equiv) was added slowly and stirred for 2 h at 0 °C. The resulting solution was added to a mixture of glucal **15a** (66 mg, 0.738 mmol, 1.0 equiv), NEt_3 (45 µL, 0.319 mmol, 1.3 equiv) and DMAP (3 mg, 0.025 mmol, 10 mol %) in dry Et_2O (10 mL) at 0 °C over a period of 20 min. The reaction was stirred over night and stopped by the addition of saturated aq.

NH_4Cl solution. The aqueous layer was extracted three times with EtOAc. The combined organic layers were washed with saturated aq. NaCl solution and dried over Na_2SO_4. The solvent was removed by rotary evaporation and the residue was purified by silica gel column chromatography (pentane/EtOAc = 30:1) to afford 106 mg (65%) of compound **14b** as a colorless oil: **R$_f$**: 0.26 (hexane/EtOAc = 20:1). $[\alpha]_D^{20}$ = +23.2° (c = 0.10, $CHCl_3$). **^{1}H-NMR** (300 MHz, $CDCl_3$): δ = 0.42 (s, 6 H, Me_{DMPS}), 1.06–1.16 (m, 14 H, *i*Pr), 1.38 (s, 3 H, 8-H), 1.48 (s, 3 H, 9-H), 3.72 (s, 2 H, 3'-H), 3.72 (s, 2 H, 10'-H), 3.76–3.84 (m, 2 H, 5-H, 6-H_a*), 3.88–3.96 (m, 2 H, 4-H*, 3-H*), 4.57 (dd, *J* = 1.4, 7.0 Hz, 1 H, 6-H_b), 6.56 (s, 1 H, 1-H) 7.24–7.29 (m, 2 H, H_{Ar}), 7.35–7.39 (m, 3 H, H_{Ar}), 7.46–7.50 (m, 1 H, H_{Ar}), 7.55–7.59 (m, 1 H, H_{Ar}), 7.62–7.66 (m, 2 H, H_{Ar}).**^{13}C-NMR** (125 MHz, $CDCl_3$): δ = –0.6 (Me_{DMPS}), 13.4, 14.1, 17.3, 17.4, 17.7, 17.8 (C_{iPr}), 18.8 (C-8), 24.0, 24.2 (C-3', C-10'), 28.9 (C-9), 61.3, 70.3, 71.6, 73.2 (C-3, C-4, C-5, C-6), 82.8, 85.5, 105.1, 105.9 (C-1', C-2', C-11', C-12'), 99.5 (C-7), 103.6 (C-2), 127.2, 127.8, 128.6, 128.7, 129.2, 133.6 (C-5', C-6', C-7', C-8', $C_{Ph,DMPS}$), 133.8, 133.9, 137.1 (C-4', C-9', $C_{q,DMPS}$), 143.5 (C-1).**MS** (ESI): *m*/*z* (%) = 689.2 (29) $[M+Na]^+$. **$C_{35}H_{45}BrO_4Si_2$** (665.80), calc.: 689.1914, found: 689.1901, $[M+Na]^+$ (ESI-HRMS).

Compound 13b

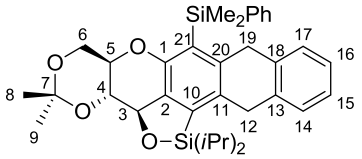

The alkynylated bromoglycal **14b** (200 mg, 0.300 mmol, 1.0 equiv), $Pd(PPh_3)_4$ (35 mg, 0.030 mmol, 10 mol %), *t*-$Bu_3PH·BF_4$ (18 mg, 0.060 mmol, 20 mol %) and diisopropylamine (213 µL, 1.50 mmol, 5.0 equiv), dissolved in DMF/MeCN/NMP (8.0 mL, 8.0 mL, 1.0 mL), were brought to reaction according to GP5. Purification by silica gel column chromatography (pentane/EtOAc = 35:1) afforded 157 mg (89%) of compound **13b** as a white solid: **R$_f$**: 0.43 (hexane/EtOAc = 6:1). $[\alpha]_D^{20}$ = 17.2° (c = 0.11, $CHCl_3$). **^{1}H-NMR** (300 MHz, $CDCl_3$): δ = 0.64 (s, 3 H, Me_{DMPS}), 0.64 (s, 3 H, Me_{DMPS}), 0.93 (d, *J* = 7.5 Hz, 3 H, $CH_{3,iPr}$), 1.00 (d, *J* = 7.4 Hz, 3 H, $CH_{3,iPr}$), 1.15 (d, *J* = 7.4 Hz, 3 H, $CH_{3,iPr}$), 1.21 (d, *J* = 7.4 Hz, 3 H, $CH_{3,iPr}$), 1.24–1.33 (m, 1 H, CH_{iPr}), 1.42–1.49 (m, 1 H, CH_{iPr}), 1.52 (s, 3 H, 8-H), 1.54 (s, 3 H, 9-H), 3.57–3.87 (m, 6 H, 4-H, 6-H_a, 12-H, 19-H), 3.87–4.01 (m, 2 H, 5-H, 6-H_b), 5.10 (d, *J* = 9.0 Hz, 1 H, 3-H), 6.78 (d, *J* = 7.2 Hz, 1 H, 14*-H), 7.03–7.20 (m, 3 H, H_{Ar}), 7.30–7.38 (m, 2 H, H_{Ar}), 7.49–7.54 (m, 2 H, H_{Ar}). **^{13}C-NMR** (125 MHz, $CDCl_3$): δ = 1.5, 1.9 (Me_{DMPS}), 13.0, 13.9, 17.3, 17.5, 17.5, 17.8 (C_{iPr}), 19.1, 29.2 (C-8, C-9), 37.6, 39.2 (C-12, C-19), 62.1 (C-6), 70.2 (C-5), 72.4 (C-4), 78.1 (C-3), 99.9 (C-7), 121.4 (C-21), 125.7, 125.9, 126.0, 126.7, 127.8, 128.6, 133.5, 137.4 (C-14, C-15, C-16, C-17, C-20, $C_{Ph,DMPS}$, $C_{q,Ph,DMPS}$), 130.1, 133.7, 135.7 (C-10, C-13, C-18), 140.8 (C-11), 145.1 (C-2), 154.4 (C-1). **IR** (ATR): ṽ (cm^{-1}) = 2941, 2862, 1538, 1381, 1247,

1091. **UV** (CH_3CN): λ_{max} [nm] (lg ε) = 307 (3.72), 211 (4.68). **MS** (ESI): *m*/*z* (%) = 607.3 (100) $[M+Na]^+$. **$C_{35}H_{44}O_4Si_2$** (584.89), calc.: 607.2651, found: 607.2670, $[M+Na]^+$ (ESI-HRMS).

Compound 22c

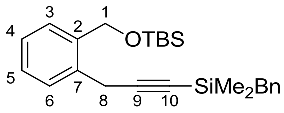

To a solution of dimethylbenzylsilylacetylene (433 mg, 2.48 mmol, 2.0 equiv) in THF (16 mL) was added ethyl magnesium bromide (3.0 mol/L in diethyl ether, 0.83 mL, 2.48 mmol, 2.0 equiv) at room temperature. The solution was heated to reflux for 2 h and then cooled to ambient temperature. CuCl (62 mg, 0.621 mmol, 50 mol %) and **20** (450 mg, 1.24 mmol, 1.0 equiv) were added to the reaction mixture and heated to 75 °C for 16 h. The reaction was stopped by addition of saturated aq. NH_4Cl solution. The aqueous layer was extracted three times with EtOAc. The combined organic layers were washed with saturated aq. NaCl solution and dried over Na_2SO_4. The solvent was removed by rotary evaporation to afford **22c** as crude product.

Yield: not determined. **R_f**: 0.54 (hexane/EtOAc = 10:1). **^{1}H-NMR** (300 MHz, $CDCl_3$): δ = 0.10 (s, 3 H, Me), 0.10 (s, 3 H, Me), 0.13 (s, 3 H, Me_{TBS}), 0.14 (s, 3 H, Me_{TBS}), 0.94–0.95 (m, 9 H, *t*Bu_{TBS}), 2.20 (s, 2 H, $CH_{2,Bn}$), 3.64 (s, 2 H, 8-H), 4.76 (s, 2 H, 1-H), 7.02–7.12 (m, 3 H, H_{Ar}), 7.16–7.23 (m, 2 H, H_{Ar}), 7.25–7.29 (m, 2 H, H_{Ar}), 7.39–7.45 (m, 2 H, H_{Ar}). **^{13}C-NMR** (125 MHz, $CDCl_3$): δ = –5.3 (Me_{TBS}), 2.0 (Me), 18.4 ($C_{q,TBS}$), 23.3 (C-8), 26.0 (*t*Bu_{TBS}), 26.4 (*C*$H_{2,Bn}$), 63.1 (C-1), 85.5 (C-9), 105.2 (C-10), 124.3, 126.8, 127.0, 127.4, 128.1, 128.3, 128.5 (C-3, C-4, C-5, C-6, CH_{Bn}), 133.5, 138.6, 139.1 (C-2, C-7, $C_{q,Bn}$). **IR** (ATR): ṽ (cm^{-1}) = 2942, 2863, 2174, 2066, 1601, 1492, 1453, 1249. **UV** (CH_3CN): λ_{max} [nm] (lg ε) = 194 (5.06). **MS** (ESI): *m*/*z* (%) = 431.2 (100) $[M+Na]^+$. **$C_{25}H_{36}OSi_2$** (408.72), calc.: 431.2197, found: 431.2191, $[M+Na]^+$ (ESI-HRMS).

Compound 23c

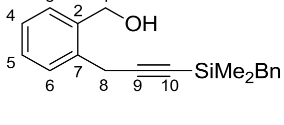

The crude product **22c** was dissolved in MeOH (8 mL) at 0 °C and AcCl (0.8 mL) was added dropwise. The reaction mixture was stirred for 3 h and then stopped by slow addition of saturated aq. $NaHCO_3$ solution. The aqueous layer was extracted three times with EtOAc. The combined organic layers were washed with saturated aq. NaCl solution and dried over Na_2SO_4. The solvent was removed by rotary evaporation and the residue was purified by silica gel column chromatography (pentane/EtOAc = 7:1) to afford 224 mg (62% over two steps) of compound **23c** as a colorless oil: **R_f**: 0.08 (hexane/EtOAc = 8:1). **^{1}H-NMR**

(300 MHz, $CDCl_3$): δ = 0.13 (s, 6 H, Me), 2.19 (s, 2 H, C$H_{2,Bn}$), 3.71 (s, 2 H, 8-H), 4.72 (d, *J* = 5.8 Hz, 2 H, 1-H), 7.00–7.12 (m, 3 H, H_{Ar}), 7.13–7.25 (m, 2 H, H_{Ar}), 7.25–7.35 (m, 2 H, H_{Ar}), 7.35–7.44 (m, 2 H, H_{Ar}). **^{13}C-NMR** (125 MHz, $CDCl_3$): δ = –2.0 (Me), 23.6 ($CH_{2,Bn}$), 26.3 (C-8), 63.2 (C-1), 85.7 (C-9), 105.7 (C-10), 124.3, 137.2, 128.2, 128.5, 129.1 (C-3, C-4, C-5, C-6, CH_{Bn}), 128.1, 128.3 (CH_{Bn}), 134.5, 138.2, 139.0 (C-2, C-7, $C_{q,Bn}$). **IR** (ATR): ṽ (cm^{-1}) = 3317, 3059, 2889, 2173, 1559, 1492, 1248. **UV** (CH_3CN): λ_{max} [nm] (lg ε) = 197 (4.78), 260 (3.20). **MS** (ESI): *m*/*z* (%) = 317.1 (100) $[M+Na]^+$. **$C_{19}H_{22}OSi$** (294.46), calc.: 295.1513, found: 295.1511, $[M+Na]^+$ (ESI-HRMS).

Compound 24c

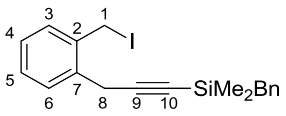

Alcohol **23c** (540 mg, 1.83 mmol, 1.0 equiv), triphenylphosphine (962 mg, 3.67 mmol, 2.0 equiv), imidazole (250 mg, 3.67 mmol, 2.0 equiv) and iodine (932 mg, 3.67 mmol, 2.0 equiv), dissolved in Et_2O (16 mL) and MeCN (4.0 mL), were brought to reaction according to GP1. Purification by silica gel column chromatography (pentane) afforded 722 mg (97%) of compound **24c** as a yellow oil: **R_f**: 0.72 (hexane/EtOAc = 10:1). **^{1}H-NMR** (300 MHz, $CDCl_3$): δ = 0.17 (s, 6 H, Me), 2.23 (s, 2 H, C$H_{2,Bn}$), 3.71 (s, 2 H, 8-H), 4.48 (s, 2 H, 1-H), 7.05–7.14 (m, 3 H, H_{Ar}), 7.19–7.27 (m, 3 H, H_{Ar}), 7.29–7.36 (m, 2 H, H_{Ar}), 7.40–7.44 (m, 1 H, H_{Ar}). **^{13}C-NMR** (125 MHz, $CDCl_3$): δ = –2.0 (Me), 3.6 (C-1), 23.6 ($CH_{2,Bn}$), 26.3 (C-8), 86.2 (C-9), 104.4 (C-10), 124.3, 127.5, 128.7, 129.5, 129.8 (C-3, C-4, C-5, C-6, CH_{Bn}), 128.1, 128.3 (CH_{Bn}), 134.7, 136.5, 139.0 (C-2, C-7, $C_{q,Bn}$). **IR** (ATR): ṽ (cm^{-1}) = 3022, 2956, 2172, 1599, 1491, 1451, 1248. **UV** (CH_3CN): λ_{max} [nm] (lg ε) = 197 (4.96). **MS** (ESI): *m*/*z* (%) = 427.0 (42) $[M+Na]^+$. **$C_{19}H_{21}ISi$** (404.36), calc.: 427.0349, found: 427.0344, $[M+Na]^+$ (ESI-HRMS).

Compound 16c

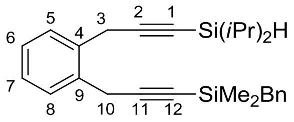

Silylacetylene **25** (490 mg, 3.49 mmol, 2.0 equiv) in THF (25 mL), ethyl magnesium bromide (3.0 mol/L in diethyl ether, 1.2 mL, 3.49 mmol, 2.0 equiv), CuCl (87 mg, 0.872 mmol, 50 mol %) and **24c** (705 g, 1.73 mmol, 1.0 equiv) in THF (7 mL) were brought to reaction according to GP5.2. Purification by silica gel column chromatography (pentane/CH_2Cl_2 = 100:1) afforded 170 mg (23%) of compound **16c** as clear oil: **R_f**: 0.54 (hexane/EtOAc = 20:1). **^{1}H-NMR** (300 MHz, $CDCl_3$): δ = 0.14 (s, 6 H, Me), 1.01–1.14 (m, 14, H_{iPr}), 2.19 (s, 2 H, C$H_{2,Bn}$), 3.64 (s, 2 H, 3-H*), 3.67 (s, 2 H, 10-H*), 3.72 (s_{br}, 1 H, Si*H*), 7.02–

7.12 (m, 3 H, H_{Ar}), 7.16–7.30 (m, 4 H, H_{Ar}), 7.38–7.42 (m, 1 H, H_{Ar}), 7.49–7.53 (m, 1 H, H_{Ar}). **^{13}C-NMR** (125 MHz, $CDCl_3$): δ = –2.0 (Me), 10.9, 18.3, 18.5 (C_{iPr}), 24.0, 24.0 (C-3, C-10), 26.4 ($CH_{2,Bn}$), 81.2, 85.8 (C-2, C-11), 104.7, 106.1 (C-1, C-12), 124.3, 127.2, 127.2, 128.1, 128.3, 128.6, 128.8 (C-5, C-6, C-7, C-8, CH_{Bn}), 133.9, 134.1, 139.0 (C-4, C-9, $C_{q,Bn}$). **IR** (ATR): ṽ (cm^{-1}) = 2941, 2862, 1600, 1492, 1453, 1249. **UV** (CH_3CN): λ_{max} [nm] (lg ε) = 194 (5.06), 266 (3.33). **MS** (ESI): *m*/*z* (%) = 434.3 (100) $[M+Na]^+$. **$C_{27}H_{36}Si_2$** (416.75), calc.: 434.2694, found: 434.2695, $[M+Na]^+$ (ESI-HRMS).

Compound 14c

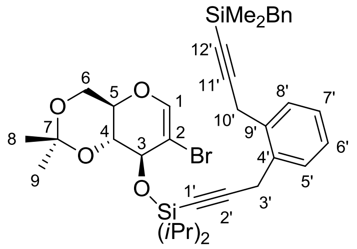

Dialkyne **16c** (100 mg, 0.240 mmol, 1.0 equiv) was dissolved in dry CCl_4 (4.0 mL) and cooled to 0 °C. A freshly prepared solution of bromine in dry CCl_4 (0.976 mol/L, 369 µL, 0.360 mmol, 1.5 equiv) was added over a period of 1 h via a syringe pump and stirred for further 2 h at 0 °C. The resulting solution was added to a mixture of glucal **15a** (77 mg, 0.288 mmol, 1.0 equiv), NEt_3 (67 µL, 0.480 mmol, 2.0 equiv) and DMAP (3 mg, 0.024 mmol, 10 mol %) in dry CCl_4 (4.0 mL) and dry Et_2O (1.0 mL) at 0 °C over a period of 30 min via syringe pump. The reaction was stirred for 2 h and stopped by the addition of saturated aq. NH_4Cl solution. The aqueous layer was extracted three times with CH_2Cl_2. The combined organic layers were washed with saturated aq. NaCl solution and dried over Na_2SO_4. The solvent was removed by rotary evaporation and the residue was purified by silica gel column chromatography (pentane/EtOAc = 100:1) to afford 56 mg (35%) of compound **14c** as a colorless oil: **R_f**: 0.46 (hexane/EtOAc = 6:1). $[\alpha]_D^{20}$ = +33.6° (c = 0.25, $CHCl_3$). **^{1}H-NMR** (300 MHz, $CDCl_3$): δ = 0.13 (s, 6 H, Me), 1.03–1.18 (m, 14 H, *i*Pr), 1.38 (s, 3 H, 8-H), 1.48 (s, 3 H, 9-H), 2.19 (s, 2 H, $CH_{2,Bn}$), 3.65 (s, 2 H, 3'-H*), 3.69 (s, 2 H, 10'-H*), 3.74–3.84 (m, 2 H, 4-H*, 6-H_a), 3.86–3.99 (m, 2 H, 5-H*, 6-H_b), 4.58 (dd, *J* = 1.3, 7.2 Hz, 1 H, 3-H), 6.56 (s, 1 H, 1-H), 7.20–7.42 (m, 8 H, H_{Ar}), 7.55–7.62 (m, 1 H, H_{Ar}). **^{13}C-NMR** (125 MHz, $CDCl_3$): δ = –2.0 (Me), 13.3, 14.0, 17.2, 17.3, 17.6, 17.7 (C_{iPr}), 18.7, 28.9 (C-8, C-9), 23.9, 24.0 (C-3', C-10'), 26.3 ($CH_{2,Bn}$), 61.3 (C-6), 70.3, 71.6, 73.2 (C-3, C-4, C-5), 82.8, 85.9 (C-2', C-11'), 99.6 (C-7), 103.7 (C-2), 104.7, 106.1 (C-1', C-12'), 124.3, 127.2, 127.2, 128.1, 128.3, 128.3, 128.7 (C-5', C-6', C-7', C-8', CH_{Bn}), 133.9, 134.0, 139.0 (C-4', C-9', $C_{q,Bn}$), 143.6 (C-1). **IR** (ATR): ṽ (cm^{-1}) = 2942, 2864, 2173, 1631, 1600, 1492. **UV** (CH_3CN): λ_{max} [nm] (lg ε) = no absorption between 190–350 nm. **MS** (ESI): *m*/*z* (%) = 703.2 (46) $[M+Na]^+$. **$C_{36}H_{47}BrO_4Si_2$** (679.83), calc.: 679.2269, found: 679.2254, $[M+H]^+$ (ESI-HRMS).

Compound 13c

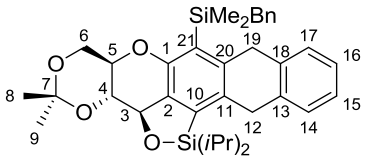

The alkynylated bromoglycal **14c** (54 mg, 0.079 mmol, 1.0 equiv), $Pd(PPh_3)_4$ (10 mg, 0.008 mmol, 10 mol %), *t*-$Bu_3PH·BF_4$ (5 mg, 0.016 mmol, 20 mol %) and diisopropylamine (57 µL, 0.397 mmol, 5.0 equiv), dissolved in DMF/MeCN/NMP (1.5 mL, 1.5 mL, 0.2 mL) were brought to reaction according to GP5. Purification by silica gel column chromatography (pentane/EtOAc = 50:1) afforded 33 mg (70%) of compound **13c** as a white solid: **R_f**: 0.49 (hexane/EtOAc = 4:1). $[\alpha]_D^{20}$ = +19.6° (c = 0.26, $CHCl_3$). **^{1}H-NMR** (300 MHz, $CDCl_3$): δ = 0.39 (s, 3 H, Me), 0.44 (s, 3 H, Me), 0.92 (d, *J* = 7.4 Hz, 3 H, $CH_{3,iPr}$), 1.00 (d, *J* = 7.3 Hz, 3 H, $CH_{3,iPr}$), 1.15 (d, *J* = 7.3 Hz, 3 H, $CH_{3,iPr}$), 1.21 (d, *J* = 7.4 Hz, 3 H, $CH_{3,iPr}$), 1.25–1.50 (m, 2 H, CH_{iPr}), 1.52 (s, 3 H, 8-H), 1.53 (s, 3 H, 9-H), 2.40 (s, 2 H, $CH_{2,Bn}$), 3.65–3.86 (m, 4 H, 4-H*, 6-H_a, 12-H), 3.86–4.01 (m, 4 H, 5-H*, 6-H_b, 19-H), 5.07 (d, *J* = 9.1 Hz, 1 H, 3-H), 6.82 (d, *J* = 7.3 Hz, 2 H, H_{Ar}), 6.97–7.13 (m, 3 H, H_{Ar}), 7.16–7.27 (m, 4 H, H_{Ar}). **^{13}C-NMR** (125 MHz, $CDCl_3$): δ = 1.2, 1.6 (Me), 12.8, 12.8, 17.1, 17.3, 17.4, 17.7 (C_{iPr}), 19.0, 29.1 (C-8, C-9), 27.1 ($CH_{2,Bn}$), 37.4, 39.2 (C-12, C-19), 62.2 (C-6), 70.1, 72.4, 78.1 (C-3, C-4, C-5), 100.0 (C-7), 122.3, 130.2, 133.6, 135.7, 137.3, 137.4, 140.0, 144.7 (C-2, C-10, C-11, C-13, C-18, C-20, C-21, $C_{q,Bn}$), 123.9, 126.0, 126.2, 126.3, 127.0 (C-14, C-15, C-16, C-17, CH_{Bn}), 127.9, 128.3 (CH_{Bn}), 154.3 (C-1). **IR** (ATR): $\tilde{\nu}$ (cm^{-1}) = 2942, 2863, 1382, 1248. **UV** (CH_3CN): λ_{max} [nm] (lg ε) = 211 (4.69), 306 (3.70). **MS** (ESI): *m*/*z* (%) = 621.3 (74) $[M+Na]^+$. **$C_{36}H_{46}O_4Si_2$** (598.92), calc.: 621.2827, found: 621.2820, $[M+Na]^+$ (ESI-HRMS).

Compound 22d

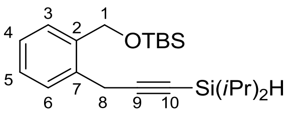

Diisopropylsilylacetylene (1.13 g, 8.05 mmol, 2.9 equiv) in THF (40 mL), ethyl magnesium bromide (3.0 mol/L in diethyl ether, 2.68 mL, 8.05 mmol, 2.9 equiv), CuCl (110 mg, 1.10 mmol, 40 mol %) and **20** (1.00 g, 2.76 mmol, 1.0 equiv) in THF (10 mL) were brought to reaction according to GP5.2. Purification by silica gel column chromatography (pentane/EtOAc = 100:1) afforded 821 mg (80%) of compound **22d** as a pale yellow oil: **R_f**: 0.60 (hexane/EtOAc = 8:1). **^{1}H-NMR** (300 MHz, $CDCl_3$): δ = 0.10 (s, 6 H, Me_{TBS}), 0.94 (s, 9 H, tBu_{TBS}), 1.03–1.12 (m, 14 H, *i*Pr), 3.68 (s, 2 H, 8-H), 3.72 (s, 1 H, Si*H*), 4.77 (s, 2 H, 1-H), 7.24–7.28 (m, 2 H, H_{Ar}), 7.39–7.43 (m, 1 H, H_{Ar}), 7.48–7.52 (m, 1 H, H_{Ar}). **^{13}C-NMR** (125 MHz, $CDCl_3$): δ = –5.3 (Me_{TBS}), 10.9, 18.3, 18.4 (C_{iPr}), 18.4 ($C_{q,TBS}$), 23.4 (C-8), 25.9 (tBu_{TBS}),

80.7, 106.5 (C-9, C-10), 126.8, 126.9, 127.4, 128.4 (C-3, C-4, C-5, C-6), 133.5, 138.5 (C-2, C-7). **IR** (ATR): $\tilde{\nu}$ (cm^{-1}) = 2928, 2861, 2174, 2116, 1460, 1254, 1074. **UV** (CH_3CN): λ_{max} [nm] (lg ε) = 193 (4.73), 252 (2.74). **MS** (ESI): *m*/*z* (%) = 397.2 (100) $[M+Na]^+$. **$C_{22}H_{38}OSi_2$** (374.71), calc.: 397.2353, found: 397.2354, $[M+Na]^+$ (ESI-HRMS).

Compound 23d

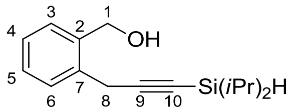

The protected alcohol **22d** (810 mg, 2.162 mmol, 1.0 equiv), dissolved in MeOH (8.0 mL), and AcCl (0.8 mL) were brought to reaction according to GP5.3. Purification by silica gel column chromatography (pentane/EtOAc = 10:1) afforded 464 mg (83%) of compound **23d** as a colorless oil: **R_f**: 0.20 (hexane/EtOAc = 8:1). **^{1}H-NMR** (300 MHz, $CDCl_3$): δ = 0.97–1.16 (m, 14 H, *i*Pr), 3.71 (s, 1 H, Si*H*), 3.76 (s, 2 H, 8-H), 4.74 (d, *J* = 5.8 Hz, 2 H, 1-H), 7.24–7.32 (m, 2 H, H_{Ar}), 7.31–7.42 (m, 1 H, H_{Ar}), 7.46–7.55 (m, 1 H, H_{Ar}). **^{13}C-NMR** (150 MHz, $CDCl_3$): δ = 10.9, 18.3, 18.5 (C_{iPr}), 23.7 (C-8), 63.3 (C-1), 81.1, 106.9 (C-9, C-10), 127.2, 128.2, 128.4, 128.9 (C-3, C-4, C-5, C-6), 134.6, 138.1 (C-2, C-7). **IR** (ATR): $\tilde{\nu}$ (cm^{-1}) = 3307, 2940, 2862, 2175, 2115, 1457. **UV** (CH_3CN): λ_{max} [nm] (lg ε) = 259 (2.86). **MS** (ESI): *m*/*z* (%) = 283.2 (100) $[M+Na]^+$. **$C_{16}H_{24}OSi$** (260.45), calc.: 283.1489, found: 283.1491, $[M+Na]^+$ (ESI-HRMS)

Compound 23d-2

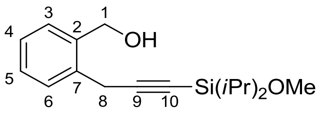

Alkyne **23d** (248 mg, 1.708 mmol, 1.2 equiv) and MeOH (1.2 mL) were dissolved in dry CCl_4 (15 mL) and cooled to 0 °C. A freshly prepared solution of bromine in dry CCl_4 (0.976 mol/L, 975 µL, 0.952 mmol, 1.0 equiv) was added over a period of 30 min via syringe pump and stirred for further 15 min at 0 °C. To this solution NEt_3 (221 µL, 1.589 mmol, 2.0 equiv) was added and stirred for 1 h. The reaction was stopped by the addition of saturated aq. NH_4Cl solution. The aqueous layer was extracted three times with EtOAc. The combined organic layers were washed with saturated aq. NaCl solution and dried over Na_2SO_4. The solvent was removed by rotary evaporation and the residue was purified by silica gel column chromatography (pentane/EtOAc = 10:1) to afford 201 mg (87%) of compound **23d-2** as a colorless oil: **R_f**: 0.14 (hexane/EtOAc = 8:1). **^{1}H-NMR** (300 MHz, $CDCl_3$): δ = 1.00–1.40 (m, 14 H, *i*Pr), 3.55 (s, 3 H, OC*H*$_3$), 3.80 (s, 2 H, 8-H), 7.78 (d, *J* = 6.0 Hz, 2 H, 1-H), 7.23–7.39 (m, 3 H, H_{Ar}), 7.50–7.57 (m, 1 H, H_{Ar}). **^{13}C-NMR** (125 MHz, $CDCl_3$): δ = 12.9, 17.0, 17.2 (C_{iPr}), 23.4 (C-8), 52.3 (OMe), 63.3 (C-1), 81.9, 106.0 (C-9, C-10), 127.2, 128.3, 128.4, 128.9 (C-3,

C-4, C-5, C-6), 134.6, 138.1 (C-2, C-7). **IR** (ATR): ṽ (cm^{-1}) = 3350, 2941, 2864, 2172, 1461, 1094. **UV** (CH_3CN): λ_{max} [nm] (lg ε) = 193 (4.70), 261 (2.24). **MS** (ESI): *m*/*z* (%) = 313.2 (100) $[M+Na]^+$. $\mathbf{C_{17}H_{26}O_2Si}$ (290.47), calc.: 313.1594, found: 313.1596, $[M+Na]^+$ (ESI-HRMS).

Compound 24d

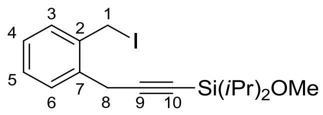

Alcohol **23d-2** (440 mg, 1.51 mmol, 1.0 equiv), triphenylphosphine (596 mg, 2.27 mmol, 1.5 equiv), imidazole (155 mg, 2.27 mmol, 1.5 equiv) and iodine (577 mg, 2.27 mmol, 1.5 equiv), dissolved in Et_2O (12 mL) and MeCN (3.0 mL) were brought to reaction according to GP1. Purification by silica gel column chromatography (pentane) afforded 403 mg (67%) of compound **24d** as a pale yellow oil: $\mathbf{R_f}$: 0.67 (hexane/EtOAc = 20:1). ^{1}**H-NMR** (300 MHz, $CDCl_3$): δ = 1.00–1.10 (m, 14 H, iPr), 3.56 (s, 3 H, OMe), 3.75 (s, 2 H, 8-H), 4.49 (s, 2 H, 1-H), 7.16–7.33 (m, 3 H, H_{Ar}), 7.48–7.53 (m, 1 H, H_{Ar}). ^{13}**C-NMR** (125 MHz, $CDCl_3$): δ = 3.4 (C-1), 12.9, 17.1, 17.3 (C_{iPr}), 23.4 (C-8), 52.3 (OMe), 82.4, 104.8 (C-9, C-10), 127.5, 128.8, 129.3, 129.8 (C-3, C-4, C-5, C-6), 134.6, 136.3 (C-2, C-7). **IR** (ATR): ṽ (cm^{-1}) = 2940, 2863, 2172, 1461, 1151. **UV** (CH_3CN): λ_{max} [nm] (lg ε) = 241 (3.90). **MS** (ESI): *m*/*z* (%) = 423.1 (100) $[M+Na]^+$. $\mathbf{C_{17}H_{25}IOSi}$ (400.37), calc.: 423.0612, found: 423.0604, $[M+Na]^+$ (ESI-HRMS).

Compound 16d

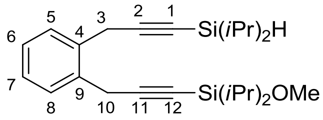

Silylacetylene **25** (400 mg, 2.884 mmol, 3.0 equiv) in THF (18 mL), ethyl magnesium bromide (3.0 mol/L in diethyl ether, 0.96 mL, 2.884 mmol, 3.0 equiv), CuCl (39 mg, 0.385 mmol, 40 mol %) and **24d** (385 g, 0.962 mmol, 1.0 equiv) in THF (6.0 mL) were brought to reaction according to GP5.2. Purification by silica gel column chromatography (pentane/CH_2Cl_2 = 150:1) afforded 173 mg (43%) of compound **16d** as a pale yellow oil: $\mathbf{R_f}$: 0.39 (hexane/EtOAc = 20:1). ^{1}**H-NMR** (300 MHz, $CDCl_3$): δ = 0.99–1.12 (m, 28 H, *i*Pr), 3.57 (s, 3 H, OMe), 3.71 (s, 2 H, 3-H*), 3.71 (s, 1 H, Si*H*), 3.73 (s, 2 H, 10-H*), 7.22–7.31 (m, 2 H, H_{Ar}), 7.44–7.56 (m, 2 H, H_{Ar}). ^{13}**C-NMR** (125 MHz, $CDCl_3$): δ = 10.9, 12.9, 17.0, 17.2, 18.2, 18.5 (C_{iPr}), 23.8, 24.0 (C-3, C-10), 52.3 (OMe), 81.2, 82.0, 105.2, 106.0 (C-1, C-2, C-11, C-12), 127.2, 127.2, 128.6, 128.7 (C-5, C-6, C-7, C-8), 133.9, 134.0 (C-4, C-9). **IR** (ATR): ṽ (cm^{-1}) = 2941, 2863, 2172, 2117, 1461, 1095. **UV** (CH_3CN): λ_{max} [nm] (lg ε) = 193 (4.75), 253 (2.55). **MS** (ESI): *m*/*z* (%) = 435.3 (100) $[M+Na]^+$. $\mathbf{C_{25}H_{40}OSi_2}$ (412.76), calc.: 435.2510, found: 435.2503, $[M+Na]^+$ (ESI-HRMS).

Compound 14d

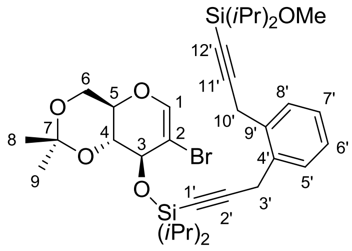

Dialkyne **16d** (165 mg, 0.396 mmol, 1.2 equiv) was dissolved in dry CCl_4 (6.0 mL) and cooled to 0 °C. A freshly prepared solution of bromine in dry CCl_4 (0.976 mol/L, 406 µL, 0.396 mmol, 1.2 equiv) was added over a period of 1 h via syringe pump and stirred for further 2 h at 0 °C. The resulting solution was added to a mixture of glucal **15a** (88 mg, 0.330 mmol, 1.0 equiv), NEt_3 (92 µL, 0.660 mmol, 2.0 equiv) and DMAP (5 mg, 0.040 mmol, 10 mol %) in dry CCl_4 (4.0 mL) and dry Et_2O (0.5 mL) at 0 °C over a period of 30 min via syringe pump. The reaction was stirred over night and stopped by the addition of saturated aq. NH_4Cl solution. The aqueous layer was extracted three times with CH_2Cl_2. The combined organic layers were washed with saturated aq. NaCl solution and dried over Na_2SO_4. The solvent was removed by rotary evaporation and the residue was purified by silica gel column chromatography (pentane/EtOAc = 100:1) to afford 78 mg (30%) of compound **14d** as a colorless oil: **R_f**: 0.35 (hexane/EtOAc = 10:1). $[\alpha]_D^{20}$ = +60.3° (c = 0.32, $CHCl_3$). **^{1}H-NMR** (300 MHz, $CDCl_3$): δ = 1.00–1.15 (m, 28 H, *i*Pr), 1.38 (s, 3 H, 8-H), 1.47 (s, 3 H, 9-H), 3.57 (s, 3 H, OMe), 3.73 (s, 2 H, 3'-H*), 3.74 (s, 2 H, 10'-H*), 3.75–3.86 (m, 2 H, 4-H*, 6-H_a), 3.88–3.97 (m, 2 H, 5-H*, 6-H_b), 4.56 (dd, *J* = 1.3, 7.0 Hz, 1 H, 3-H), 6.56 (d, *J* = 1.3 Hz, 1 H, 1-H), 7.22–7.33 (m, 2 H, H_{Ar}), 7.47–7.60 (m, 2 H, H_{Ar}). **^{13}C-NMR** (150 MHz, $CDCl_3$): δ = 13.0, 13.3, 14.1, 17.1, 17.3, 17.3, 17.3, 17.6, 17.7 (C_{iPr}), 18.8, 28.9 (C-8, C-9), 24.0, 24.0 (C-3', C-10'), 52.3 (OMe), 61.3 (C-6), 70.3, 71.6, 73.2 (C-3, C-4, C-5), 82.1, 82.8, 105.2, 105.8 (C-1', C-2', C-11', C-12'), 99.6 (C-7), 103.6 (C-2), 127.2, 127.2, 128.5, 128.7 (C-5', C-6', C-7', C-8'), 133.8, 133.8 (C-4', C-9'), 143.6 (C-1). **IR** (ATR): $\tilde{\nu}$ (cm^{-1}) = 3419, 2942, 2864, 2172, 1632, 1462, 1171. **UV** (CH_3CN): λ_{max} [nm] (lg ε) = 194 (5.13). **MS** (ESI): *m*/*z* (%) = 694.3 (100) $[M+NH_4]^+$. **$C_{34}H_{51}BrO_5Si_2$** (675.84), calc.: 694.2782, found: 694.2772, $[M+NH_4]^+$ (ESI-HRMS).

Compound 13d

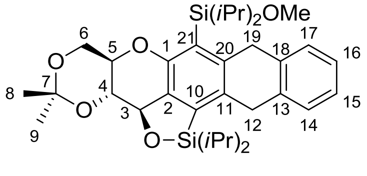

The alkynylated bromoglycal **14d** (70 mg, 0.104 mmol, 1.0 equiv), $Pd(PPh_3)_4$ (12 mg, 0.010 mmol, 10 mol %), *t*-$Bu_3PH{\cdot}BF_4$ (6 mg, 0.021 mmol, 20 mol %) and diisopropylamine (74 µL, 0.518 mmol, 5.0 equiv), dissolved in DMF/MeCN/NMP (2.3 mL, 2.3 mL, 0.3 mL) were

brought to reaction according to GP5. Purification by silica gel column chromatography (pentane/EtOAc = 50:1) afforded 53 mg (86%) of compound **13d** as a pale yellow solid: **R_f**: 0.29 (hexane/EtOAc = 10:1). **^{1}H-NMR** (300 MHz, $CDCl_3$): δ = 0.88 (d, *J* = 7.5 Hz, 3 H, Me_{iPr}), 0.92 (d, *J* = 7.5 Hz, 3 H, Me_{iPr}), 0.96 (d, *J* = 7.4 Hz, 3 H, Me_{iPr}), 1.04 (d, *J* = 7.4 Hz, 3 H, Me_{iPr}), 1.05 (d, *J* = 7.5 Hz, 3 H, Me_{iPr}), 1.10 (d, *J* = 7.3 Hz, 3 H, Me_{iPr}), 1.14 (d, *J* = 7.3 Hz, 3 H, Me_{iPr}), 1.21 (d, *J* = 7.4 Hz, 3 H, Me_{iPr}), 1.24–1.48 (m, 4 H, CH_{iPr}), 1.50 (s, 3 H, 8-H), 1.53 (s, 3 H, 9-H), 3.66 (s, 3 H, OMe), 3.70–3.91 (m, 4 H, 4-H*, 6-H_a, 12-H), 3.92–4.05 (m, 2 H, 5-H*, 6-H_b), 4.11 (d, *J* = 16.4 Hz, 1 H, 19-H_a), 4.28 (d, *J* = 16.6 Hz, 1 H, 19-H_b), 5.08 (d, *J* = 9.0 Hz, 1 H, 3-H), 7.14–7.30 (m, 4 H, H_{Ar}).**^{13}C-NMR** (125 MHz, $CDCl_3$): δ = 12.9, 13.8, 14.4, 14.4, 17.1, 17.3, 17.4, 17.6, 17.6, 17.7, 18.1, 18.2 (C_{iPr}), 19.0, 29.1 (C-8, C-9), 35.6, 39.6 (C-12, C-19), 51.9 (OMe), 62.1 (C-6), 70.4, 72.5, 78.0 (C-3, C-4, C-5), 100.0 (C-7), 119.2, 129.5, 134.6, 135.8, 137.9, 138.6, 146.7 (C-2, C-10, C-11, C-13, C-18, C-20, C-21), 153.8 (C-1). **MS** (ESI): *m*/*z* (%) = 617.3 (100) $[M+Na]^+$. **$C_{34}H_{50}O_5Si_2$** (594.93), calc.: 617.3089, found: 617.3085, $[M+Na]^+$ (ESI-HRMS).

Compound 24e

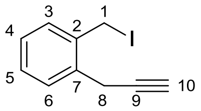

Alcohol **23e** (328 mg, 2.24 mmol, 1.0 equiv), triphenylphosphine (1832 mg, 4.49 mmol, 2.0 equiv), imidazole (305 mg, 4.49 mmol, 2.0 equiv) and iodine (1140 mg, 4.49 mmol, 2.0 equiv), dissolved in Et_2O (30 mL) and MeCN (7.5 mL) were brought to reaction according to GP1. Purification by silica gel column chromatography (pentane) afforded 533 mg (93%) of compound **24e** as a pale yellow oil.

yield: 93 %. **R_f**: 0.57 (hexane/EtOAc = 4:1). **^{1}H-NMR** (300 MHz, $CDCl_3$): δ = 2.21 (t, *J* = 2.7 Hz, 1 H, 10-H), 3.65 (d, *J* = 2.7 Hz, 2 H, 8-H), 4.49 (s, 2 H, 1-H), 7.16–7.33 (m, 3 H, H_{Ar}) 7.43–7.47(m, 1 H, H_{Ar}). **^{13}C-NMR** (125 MHz, $CDCl_3$): δ = 3.43 (C-1), 22.1 (C-8), 71.3 (C-10), 80.8 (C-9), 127.6, 128.8, 129.5, 129.9 (C-3, C-4, C-5, C-6), 134.6, 136.5 (C-2, C-7). **IR** (ATR): $\tilde{\nu}$ (cm^{-1}) = 3275, 3258, 1635, 1484, 1145. **UV** (CH_3CN): λ_{max} [nm] (lg ε) = 193 (4.45), 238 (3.90). **MS** (ESI): *m*/*z* (%) = 279.1 (16) $[M+Na]^+$. **$C_{10}H_9I$** (256.08).

Compound 16e

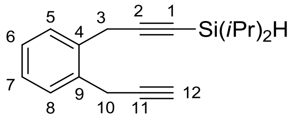

Silylacetylene **25** (838 mg, 5.97 mmol, 3.0 equiv) in THF (35 mL), ethyl magnesium bromide (3.0 mol/L in diethyl ether, 2.0 mL, 5.97 mmol, 3.0 equiv), CuCl (79 mg, 0.796 mmol,

40 mol %) and **24e** (510 g, 1.99 mmol, 1.0 equiv) in THF (12 mL) were brought to reaction according to GP5.2. Purification by silica gel column chromatography (pentane/CH_2Cl_2 = 50:1) afforded 292 mg (55%) of compound **16e** as a yellow oil: **R_f**: 0.61 (hexane/EtOAc = 10:1). **^{1}H-NMR** (300 MHz, $CDCl_3$): δ = 1.00–1.14 (m, 14 H, iPr), 2.19 (t, *J* = 2.7 Hz, 1 H, 12-H), 3.62 (d, *J* = 2.7 Hz, 2 H, 10-H), 3.71 (s, 2 H, 3-H), 3.72 (s, 1 H, Si*H*), 7.24–7.29 (m, 2 H, H_{Ar}), 7.44–7.53 (m, 2 H, H_{Ar}). **^{13}C-NMR** (125 MHz, $CDCl_3$): δ = 10.9, 18.3, 18.5 (C_{iPr}), 22.5, 24.0 (C-3, C-10), 70.9 (C-12), 81.1, 81.2 (C-2, C-11), 106.0 (C-1), 127.3, 127.4, 128.7, 128.7 (C-, C-5, C-6, C-7, C-8), 133.8, 134.0 (C-4, C-9). **IR** (ATR): ṽ (cm^{-1}) = 3307, 2941, 2862, 2174, 2116, 1455. **UV** (CH_3CN): λ_{max} [nm] (lg ε) = 193 (4.71), 260 (2.25). **MS** (ESI): *m*/*z* (%) = 291.2 (22) $[M+Na]^+$. **$C_{18}H_{24}Si$** (268.47), calc.: 291.1539, found: 291.1538, $[M+Na]^+$ (ESI-HRMS).

Compound 14e

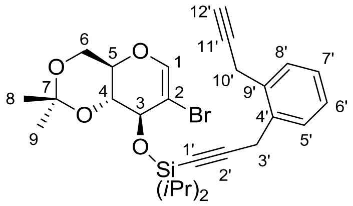

Dialkyne **16e** (284 mg, 1.06 mmol, 1.1 equiv) was dissolved in dry CCl_4 (12 mL) and cooled to 0 °C. A freshly prepared solution of bromine in dry CCl_4 (0.976 mol/L, 1.1 mL, 1.06 mmol, 1.1 equiv) was added over a period of 1 h via syringe pump and stirred for further 2 h at 0 °C. To a solution of glucal **15a** (255 mg, 0.961 mmol, 1.0 equiv), NEt_3 (267 µL, 1.922 mmol, 2.0 equiv) and DMAP (12 mg, 0.096 mmol, 10 mol %) in dry CCl_4 (6.0 mL) and dry Et_2O (1.5 mL) was added the solution of **16e** at 0 °C over a period of 30 min via syringe pump. The reaction was stirred for 2 h and stopped by the addition of saturated aq. NH_4Cl solution. The aqueous layer was extracted three times with CH_2Cl_2. The combined organic layers were washed with saturated aq. NaCl solution and dried over Na_2SO_4. The solvent was removed by rotary evaporation and the residue was purified by silica gel column chromatography (pentane/EtOAc = 60:1) to afford 285 mg (56%) of compound **14e** as a yellow oil: **R_f**: 0.42 (hexane/EtOAc = 6:1). $[\boldsymbol{\alpha}]_{\mathbf{D}}^{\mathbf{20}}$ = +57.1° (c = 0.52, $CHCl_3$). **^{1}H-NMR** (300 MHz, $CDCl_3$): δ = 1.06–1.14 (m, 14 H, *i*Pr), 1.38 (s, 3 H, 8-H), 1.48 (s, 3 H, 9-H), 2.19 (t, *J* = 2.7 Hz, 1 H, 12'-H), 3.62 (d, *J* = 2.7 Hz, 2 H, 10'-H), 3.73 (s, 2 H, 3'-H), 3.76–3.83 (m, 2 H, 4-H*, 6-H_a), 3.88–3.96 (m, 2 H, 5-H*, 6-H_b), 4.57 (dd, *J* = 1.4, 7.1 Hz, 1 H, 3-H), 6.56 (d, *J* = 1.4 Hz, 1 H, 1-H), 7.24–7.29 (m, 2 H, H_{Ar}), 7.42–7.48 (m, 1 H, H_{Ar}), 7.54–7.59 (m, 1 H, H_{Ar}). **^{13}C-NMR** (125 MHz, $CDCl_3$): δ = 13.3, 14.0, 17.2, 17.3, 17.6, 17.7 (C_{iPr}), 18.7, 28.8 (C-8, C-9), 22.6, 23.9 (C-3', C-10'), 61.3 (C-6), 70.3, 71.6, 73.2 (C-3, C-4, C-5), 71.0 (C-12'), 81.1, 82.8 (C-2', C-11'), 99.6 (C-7), 103.7 (C-2), 106.0 (C-1'), 127.3, 127.4, 128.6, 128.8 (C-5', C-6', C-7', C-8'), 133.8, 133.9 (C-4', C-9'), 143.6 (C-1). **IR** (ATR): ṽ (cm^{-1}) = 3297, 2942, 2864, 2173,

1632, 1461, 1171. **UV** (CH_3CN): λ_{max} [nm] (lg ε) = 193 (5.04). **MS** (ESI): *m*/*z* (%) = 555.1 (83) $[M+Na]^+$. **$C_{27}H_{35}BrO_4Si$** (531.55), calc.: 555.1361, found: 555.1353, $[M+Na]^+$ (ESI-HRMS).

Compound 13e

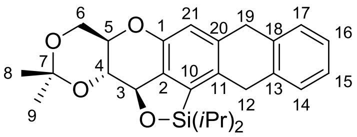

The alkynylated bromoglycal **14e** (265 mg, 0.498 mmol, 1.0 equiv), $Pd(PPh_3)_4$ (58 mg, 0.050 mmol, 10 mol %), *t*-$Bu_3PH{\cdot}BF_4$ (29 mg, 0.100 mmol, 20 mol %) and diisopropylamine (353 µL, 2.49 mmol, 5.0 equiv), dissolved in DMF/MeCN/NMP (9.5 mL, 9.5 mL, 1.2 mL) were brought to reaction according to GP5. Purification by silica gel column chromatography (pentane/EtOAc = 50:1) afforded 150 mg (67%) of compound **13e** as a white solid: **R_f**: 0.31 (hexane/EtOAc = 10:1). $[\alpha]_D^{20}$ = +58.0° (c = 0.25, $CHCl_3$). **^{1}H-NMR** (600 MHz, $CDCl_3$): δ = 0.87 (d, *J* = 7.5 Hz, 3 H, $CH_{3,iPr}$), 0.96 (d, *J* = 7.4 Hz, 3 H, $CH_{3,iPr}$), 1.13 (d, *J* = 7.4 Hz, 3 H, $CH_{3,iPr}$), 1.19 (d, *J* = 7.5 Hz, 3 H, $CH_{3,iPr}$), 1.22–1.29 (m, 1 H, CH_{iPr}), 1.40–1.48 (m, 1 H, CH_{iPr}), 1.51 (s, 3 H, 8-H), 1.53 (s, 3 H, 9-H), 3.85 (m, 6 H, 4-H, 6-H_a, 12-H, 19-H), 4.01–4.13 (m, 2 H, 5-H, 6-H_b), 5.10 (d, *J* = 9.1 Hz, 1 H, 3-H), 6.71 (s, 1 H, 21-H), 7.15–7.19 (m, 2 H, H_{Ar}), 7.21–7.23 (m, 1 H, H_{Ar}), 7.25–7.28 (m, 1 H, H_{Ar}). **^{13}C-NMR** (125 MHz, $CDCl_3$): δ = 12.8, 13.8, 17.1, 17.3, 17.4, 17.6 (C_{iPr}), 19.0, 29.1 (C-8, C-9), 36.6 (C-19), 38.0 (C-12), 62.3 (C-6), 70.5 (C-5), 72.7 (C-4), 77.7 (C-3), 100.0 (C-7), 114.8 (C-21), 126.1, 126,2 (C-15, C-16), 127.0, 127.2 (C-14, C-17), 131.3 (C-2), 133.2 (C-18), 133.8 (C-20*), 136,5 (13), 136.8 (C-11*), 138,7 (C-10), 149.2 (C-1). **IR** (ATR): ṽ (cm^{-1}) = 2941, 2863, 2360, 2341, 1585, 1575, 1445, 1253. **UV** (CH_3CN): λ_{max} [nm] (lg ε) = 263 (3.54), 294 (3.65), 309 (4.61). **MS** (ESI): *m*/*z* (%) = 473.2 (100) $[M+Na]^+$. **$C_{27}H_{34}O_4Si$** (450.64), calc.: 473.2119, found: 473.2117, $[M+Na]^+$ (ESI-HRMS).

Compound 14f

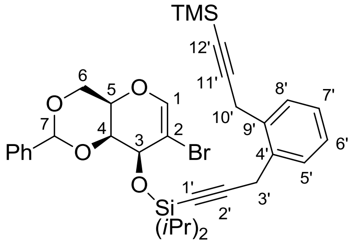

Dialkyne **16a** (177 mg, 0.520 mmol, 1.0 equiv) was dissolved in dry CCl_4 (5.0 mL) and cooled to 0 °C. A freshly prepared solution of bromine in dry CCl_4 (0.976 mol/L, 559 µL, 0.546 mmol, 1.05 equiv) was added over a period of 1 h via syringe pump and stirred for further 2 h at 0 °C. The resulting solution was added to a mixture of glucal **15c** (179 mg, 0.572 mmol, 1.1 equiv), NEt_3 (159 µL, 1.14 mmol, 2.0 equiv) and DMAP (7 mg, 0.052 mmol, 10 mol %) in

dry CCl_4 (5.0 mL) and dry Et_2O (1.0 mL) at 0 °C over a period of 30 min via syringe pump. The reaction was stirred for 2 h and stopped by the addition of saturated aq. NH_4Cl solution. The aqueous layer was extracted three times with CH_2Cl_2. The combined organic layers were washed with saturated aq. NaCl solution and dried over Na_2SO_4. The solvent was removed by rotary evaporation and the residue was purified by silica gel column chromatography (pentane/EtOAc = 80:1 → 30:1) to afford 229 mg (68%) of compound **14f** as a slightly yellow oil: **R$_f$**: 0.43 (hexane/EtOAc = 6:1). $[\alpha]_D^{20}$ = +88.4° (c = 0.25, $CHCl_3$). **^{1}H-NMR** (300 MHz, $CDCl_3$): δ = 0.18 (s, 9 H, Me_{TMS}), 0.96–1.06 (m, 7 H, C$H_{3,iPr}$, CH_{iPr}), 1.11–1.18 (m, 7 H, C$H_{3,iPr}$, CH_{iPr}), 3.63–3.77 (m, 6 H, 4-H*, 6-H$_a$, 3'-H, 10'-H), 4.15–4.27 (m, 2 H, 5-H*, 6-H$_b$), 4.73 (dd, J = 2.0, 5.0 Hz, 1 H, 3-H), 5.31 (s, 1 H, 7-H), 6.65 (d, J = 2.0 Hz, 1 H, 1-H), 7.24–7.36 (m, 5 H, H$_{Ar}$), 7.40–7.54 (m, 4 H, H$_{Ar}$). **^{13}C-NMR** (125 MHz, $CDCl_3$): δ = 0.0 (Me_{TMS}), 13.0, 13.8, 16.9, 17.0, 17.2, 17.3 (C$_{iPr}$), 23.8, 24.0 (C-3', C-10'), 67.6, 68.8, 69.2, 73.5 (C-3, C-4, C-5, C-6), 82.1, 88.1 (C-2', C-11'), 101.3 (C-7), 101.4 (C-2), 102.9, 106.1 (C-1', C-12'), 126.4, 127.4, 127.6, 128.0, 128.0, 128.8, 128.9 (C-5', C-6', C-7', C-8', CH$_{Ph}$), 134.1, 134.2, 137.4 (C-4', C-9', C$_{q,Ph}$), 143.4 (C-1). **IR** (ATR): $\tilde{\nu}$ (cm^{-1}) = 2944, 2864, 2171, 1645, 1454, 1247, 1192. **UV** (CH_3CN): λ_{max} [nm] (lg ε) = no absorption between 190–350 nm. **MS** (ESI): m/z (%) = 674.2 (45) $[M+Na]^+$. **$C_{34}H_{43}B_4O_4Si_2$** (651.78), calc.: 674.1808, found: 674.1840, $[M+Na]^+$ (ESI-HRMS).

Compound 13f

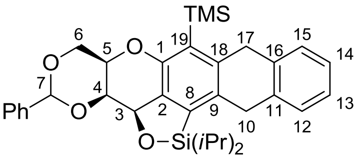

The alkynylated bromoglycal **14f** (218 mg, 0.334 mmol, 1.0 equiv), $Pd(PPh_3)_4$ (39 mg, 0.034 mmol, 10 mol %), t-$Bu_3PH\cdot BF_4$ (20 mg, 0.067 mmol, 20 mol %) and diisopropylamine (237 µL, 1.67 mmol, 5.0 equiv), dissolved in DMF/MeCN/NMP (8.8 mL, 8.8 mL, 1.1 mL) were brought to reaction according to GP5. Purification by silica gel column chromatography (pentane/EtOAc = 20:1) afforded 141 mg (74%) of compound **13f** as a white solid: **R$_f$**: 0.22 (hexane/EtOAc = 10:1). $[\alpha]_D^{20}$ = +14.6° (c = 0.13, $CHCl_3$). **^{1}H-NMR** (300 MHz, $CDCl_3$): δ = 0.47 (s, 9 H, Me_{TMS}), 0.84 (d, J = 7.4 Hz, 3 H, C$H_{3,iPr}$), 0.84 (d, J = 7.4 Hz, 3 H, C$H_{3,iPr}$), 1.13 (d, J = 7.3 Hz, 3 H, C$H_{3,iPr}$), 1.13 (d, J = 7.4 Hz, 3 H, C$H_{3,iPr}$), 1.16–1.43 (m, 2 H, CH_{iPr}), 3.80 (s, 2 H, 10-H*), 4.02–4.06 (m, 2 H, 17-H*), 4.10–4.16 (m, 2 H, 5-H, 6-H$_a$), 4.48 (dd, J = 1.9, 12.5 Hz, 1 H, 6-H$_b$), 4.56 (dd, J = 1.6, 3.2 Hz, 1 H, 4-H), 5.19 (d, J = 3.2 Hz, 1 H, 3-H), 5.60 (s, 1 H, 7-H), 7.13–7.23 (m, 8 H, H$_{Ar}$), 7.27–7.31 (m, 1 H, H$_{Ar}$). **^{13}C-NMR** (125 MHz, $CDCl_3$): δ = 2.7 (Me_{TMS}), 13.2, 13.9, 17.1, 17.3, 17.4, 17.7 (C$_{iPr}$), 37.3, 39.2 (C-10, C-17), 68.8, 70.0, 73.1, 76.1 (C-3, C-4, C-5, C-6), 100.7 (C-7), 123.5, 128.3, 132.5, 133.7, 137.7,

137.8, 137.9, 143.7 (C-2, C-8, C-9, C-11, C-16, C-18, C-19, $C_{q,Ph}$), 125.9, 126.0, 126.4, 126.4, 126.9, 127.7, 128.6 (C-12, C-13, C-14, C-15, CH_{Ph}), 154.9 (C-1). **IR** (ATR): $\tilde{\nu}$ (cm^{-1}) = 2942, 2862, 1542, 1458, 1384, 1247. **UV** (CH_3CN): λ_{max} [nm] (lg ε) = 209 (4.70), 305 (3.70). **MS** (ESI): *m*/*z* (%) = 593.3 (100) $[M+Na]^+$. $\mathbf{C_{34}H_{42}O_4Si_2}$ (570.87), calc.: 593.2514, found: 593.2510, $[M+Na]^+$ (ESI-HRMS).

Compound 26b

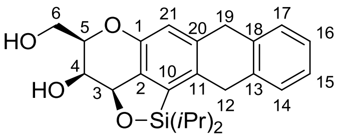

To a solution of **13f** (130 mg, 0.228 mmol, 1.0 equiv) in MeOH (10 mL) was added dropwise AcCl (1.0 mL) at 0 °C. The reaction mixture was stirred at 60 °C for 3.5 h and then stopped by slow addition of saturated aq. $NaHCO_3$ solution. The aqueous layer was extracted three times with EtOAc. The combined organic layers were washed with saturated aq. NaCl solution and dried over Na_2SO_4. The solvent was removed by rotary evaporation and the residue was purified by silica gel column chromatography to afford 78 mg (83%) of compound **26b** as a white solid: $\mathbf{R_f}$: 0.47 (CH_2Cl_2/MeOH = 8:1). $[\boldsymbol{\alpha}]_{\mathbf{D}}^{\mathbf{20}}$ = +34.7° (c = 0.15, $CHCl_3$). **^{1}H-NMR** (300 MHz, $CDCl_3$): δ = 0.92 (d, *J* = 7.5 Hz, 3 H, $CH_{3,iPr}$), 1.09 (d, *J* = 7.4 Hz, 3 H, $CH_{3,iPr}$), 1.14 (d, *J* = 7.4 Hz, 3 H, $CH_{3,iPr}$), 1.20 (d, *J* = 7.4 Hz, 3 H, $CH_{3,iPr}$), 1.23–1.51 (m, 2 H, CH_{iPr}), 2.06 (s_{br}, 1 H, O*H*), 2.38 (s_{br}, 1 H, O*H*), 3.80–3.95 (m, 4 H, 12-H, 19-H), 4.02 (dd, *J* = 4.7, 11.7 Hz, 1 H, 6-H_a), 4.11 (dd, *J* = 5.9, 11.7 Hz, 1 H, 6-H_b), 4.24–4.30 (m, 1 H, 5-H), 4.40 (d, *J* = 3.2 Hz, 1 H, 3-H*), 5.18 (d, *J* = 3.2 Hz, 1 H, 4-H*), 6.83 (s, 1 H, 21-H), 7.16–7.32 (m, 4 H, 14-H, 15-H, 16-H, 17-H). **^{13}C-NMR** (125 MHz, $CDCl_3$): δ = 13.0, 13.9, 17.2, 17.3, 17.6, 17.6 (C_{iPr}), 36.7, 38.0 (C-12, C-19), 63.6, 67.1, 76.2, 76.8 (C-3, C-4, C-5, C-6), 115.1 (C-21), 126.1, 126.1, 126.9, 127.3 (C-14, C-15, C-16, C-17), 129.2, 132.9, 133.5, 136.7, 136.8, 138.7 (C-2, C-10, C-11, C-13, C-18, C-20), 149.7 (C-1). **IR** (ATR): $\tilde{\nu}$ (cm^{-1}) = 3385, 2940, 2862, 1587, 1447, 1261, 1070. **UV** (CH_3CN): λ_{max} [nm] (lg ε) = 262 (3.62), 289 (3.65). **MS** (ESI): *m*/*z* (%) = 433.2 (23) $[M+Na]^+$. $\mathbf{C_{24}H_{30}O_4Si}$ (410.58), calc.: 433.1806, found: 433.1800, $[M+Na]^+$ (ESI-HRMS).

Compound 12b

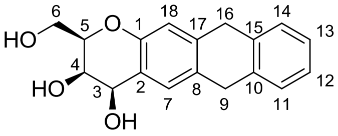

To a solution of **26b** (73 mg, 0.178 mmol, 1.0 equiv) in THF (0.1 mL) was added TBAF (1.0 mol/L in THF, 711 µL, 0.711 mmol, 3.0 equiv). The mixture was stirred for 4 h and directly poured on a packed silica gel column (CH_2Cl_2/MeOH = 10:1). 55 mg (99%) of

compound **12b** was obtained as a yellow solid after chromatographic purification: **R$_f$**: 0.26 (CH_2Cl_2/MeOH = 10:1). $[\alpha]_D^{20}$ = +67.0° (c = 0.10, DMSO). **^{1}H-NMR** (300 MHz, DMSO-d_6): δ = 3.61–3.76 (m, 2 H, 6-H_a, 6-H_b), 3.81 (s, 4 H, 9-H, 16-H), 3.93 (dt, *J* = 1.1, 3.8 Hz, 1 H, 3-H), 4.01 (t, *J* = 6.3 Hz, 1 H, 5-H), 4.57 (d, *J* = 3.9 Hz, 1 H, O$H_{C\text{-}3}$), 4.70 (dd, *J* = 3.8, 7.5 Hz, 1 H, 4-H), 4.78 (t, *J* = 5.6 Hz, 1 H, O$H_{C\text{-}6}$), 5.08 (d, *J* = 7.6 Hz, 1 H, O$H_{C\text{-}4}$), 6.65 (s, 1 H, 18-H), 7.13–7.19 (m, 2 H, 12-H, 13-H), 7.25–7.30 (m, 2 H, 11-H, 14-H), 7.31 (s, 1 H, 7-H). **^{13}C-NMR** (125 MHz, DMSO-d_6): δ = 34.5, 35.0 (C-9, C-16), 60.6 (C-6), 64.7 (C-3), 66.3 (C-4), 77.5 (C-5), 113.3 (C-18), 122,7, 127,7, 136,0, 136,4, 137,0 (C-2, C-8, C-10, C-15, C-17), 125.7, 125.7, 126.3, 127.0, 127,0 (C-7, C-11, C-12, C-13, C-14), 151.7 (C-1). **IR** (ATR): $\tilde{\nu}$ (cm^{-1}) = 3255, 2919, 2344, 1672, 1295. **UV** (CH_3CN): λ_{max} [nm] (lg ε) = 261 (4.22). **MS** (ESI): *m*/*z* (%) = 321.1 (15) $[M+Na]^+$. **$C_{18}H_{18}O_4$** (298.33), calc.: 321.1097, found: 321.1096, $[M+Na]^+$ (ESI-HRMS).

Compound 28b

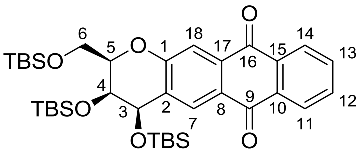

Compound **12b** (29 mg, 0.099 mmol, 1.0 equiv), TBSCl (224 mg, 1.48 mmol, 15.0 equiv), imidazole (303 mg, 4.45 mmol, 45.0 equiv) and DMAP (19 mg, 0.148 mmol, 1.0 equiv) were dissolved in DMF (8.0 mL). The reaction mixture was heated in a sealed vial at 90 °C for 68 h. TLC still showed no complete conversion. The mixture of products was purified and the crude product was again set to reaction according the described procedure for 18 h. The reaction was stopped by the addition of saturated aq. NH_4Cl solution. The aqueous layer was extracted three times with EtOAc. The combined organic layers were washed three times with saturated aq. NaCl solution and dried over Na_2SO_4. The solvent was removed by rotary evaporation and the residue was purified by silica gel column chromatography (pentane/EtOAc = 100:1) to afford 18 mg (30%) of compound **27b** as a colorless oil.

Compound **27b** (18 mg, 0.028 mmol, 1.0 equiv), $FeCl_3 \cdot 6H_2O$ (2 mg, 0.006 mmol, 20 mol %) and *t*-BuOOH (5.5 mol/L in decane, 52 µL, 0.281 mmol, 10.0 equiv) were dissolved in dry pyridine (2.0 mL). The reaction mixture was aerated with molecular oxygen and heated in a sealed vial at 80 °C for 3 h. After common aqueous workup (general procedures) the residue was purified by silica gel column chromatography (pentane/EtOAc = 25:1) to afford 11 mg (59%) of compound **28b** as a yellow solid: **R$_f$**: 0.21 (hexane/EtOAc = 20:1). $[\alpha]_D^{20}$ = -20.0° (c = 0.10, $CHCl_3$). **^{1}H-NMR** (300 MHz, $CDCl_3$): δ = 0.01 (s, 3 H, Me_{TBS}), 0.09 (s, 3 H, Me_{TBS}), 0.11 (s, 3 H, Me_{TBS}), 0.13 (s, 3 H, Me_{TBS}), 0.24 (s, 3 H, Me_{TBS}), 0.27 (s, 3 H, Me_{TBS}), 0.68 (s, 9 H, *t*Bu_{TBS}), 0.93 (s, 9 H, *t*Bu_{TBS}), 1.07 (s, 9 H, *t*Bu_{TBS}), 3.87–3.92 (m, 2 H, 5-H*, 6-H_a*),

4.29–4.36 (m, 2 H, 4-H*, 6-H_b*), 4.96 (s, 1 H, 3-H*), 7.57 (s, 1 H, 18-H), 7.73–7.78 (m, 2 H, 12-H, 13-H), 8.25–8.31 (m, 2 H, 11-H, 14-H), 8.33 (s, 1 H, 7-H). **^{13}C-NMR** (125 MHz, $CDCl_3$): δ = –5.3, –5.2, –4.9, –4.4, –3.9 (Me_{TBS}), 18.2, 18.3, 18.8 ($C_{q,TBS}$), 25.7, 25.8, 26.3 ($t$$Bu_{TBS}$), 61.5, 67.5, 70.9, 80.2 (C-3, C-4, C-5, C-6), 113.0 (C-18), 126.5, 133.8, 133.9, 134.6, 134.6 (C-2, C-8, C-10, C-15, C-17), 127.0, 127.0, 133.5, 133.9 (C-11, C-12, C-13, C-14), 131,7 (C-7), 159.0 (C-1), 182.1, 183.2 (C-9, C-16). **IR** (ATR): ṽ (cm^{-1}) = 2927, 2855, 1674, 1592, 1292. **UV** (CH_3CN): λ_{max} [nm] (lg ε) = 276 (3.91). **MS** (ESI): *m*/*z* (%) = 692.3 (65) $[M+Na]^+$. **$C_{36}H_{56}O_6Si_3$** (669.08), calc.: 691.3277, found: 691.3266, $[M+Na]^+$ (ESI-HRMS).

Compound 11b

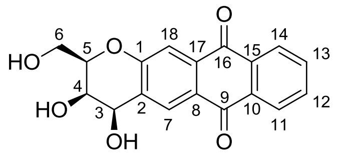

To a solution of **28b** (8 mg, 0.012 mmol, 1.0 equiv) in MeOH (1.0 mL) and water (0.1 mL) was added HCl (0.3 mol/L, 0.4 mL). The mixture was stirred for 17 h at 75 °C and stopped by the addition of saturated aq. $NaHCO_3$ solution. The aqueous layer was extracted three times with EtOAc. The combined organic layers were washed with saturated aq. NaCl solution and dried over Na_2SO_4. The solvent was removed by rotary evaporation and the residue was filtered over a small plug of celite (CH_2Cl_2: 20 mL and CH_2Cl_2/MeOH = 5:1: 250 mL). The product-containing fractions were collected, the solvent was removed by rotary evaporation and the residue was washed with water (50 mL). 3.5 mg (90%) of compound **11b** were obtained as a yellow solid: **R_f**: 0.16 (CH_2Cl_2/MeOH = 10:1). $[\boldsymbol{\alpha}]_{\mathbf{D}}^{\mathbf{20}}$ = -10.0° (c = 0.30, DMSO). **^{1}H-NMR** (300 MHz, DMSO-d_6): δ = 3.72 (s_{br}, 2 H), 4.06 (s_{br}, 1 H), 4.31 (s_{br}, 1 H), 4.84 (s_{br}, 1 H), 5.08 (s_{br}, 1 H), 5.08 (s_{br}, 1 H), 5.30 (s_{br}, 1 H), 5.94 (s_{br}, 1 H), 7.31 (s, 1 H, 18-H), 7.88 (s_{br}, 2 H, 12-H, 13-H), 8.15 (s_{br}, 2 H, 11-H, 14-H), 8.28 (s, 1 H, 7-H). **^{13}C-NMR** (125 MHz, DMSO-d_6): δ = 60.2, 64.1, 66.5, 79.2 (C-3, C-4, C-5, C-6), 112.1 (C-18), 125.7, 133.1, 133.1, 133.2, 133.6 (C-2, C-8, C-10, C-15, C-17), 126.5, 126.6, 127.7, 134.0, 134.4 (C-7, C-11, C-12, C-13, C-14), 159.0 (C-1), 181.2, 182.1 (C-9, C-16). **IR** (ATR): ṽ (cm^{-1}) = 3281, 2922, 1667, 1588, 1565, 1332, 1287. **UV** (MeOH): λ_{max} [nm] (lg ε) = 239 (3.99), 276 (4.21). **MS** (ESI): *m*/*z* (%) = 349.1 (89) $[M+Na]^+$. **$C_{18}H_{14}O_6$** (326.30), calc.: 325.0718, found: 325.0714, $[M-H]^-$ (ESI-HRMS).

^{1}H and ^{13}C NMR spectra of the compounds

Compound 20

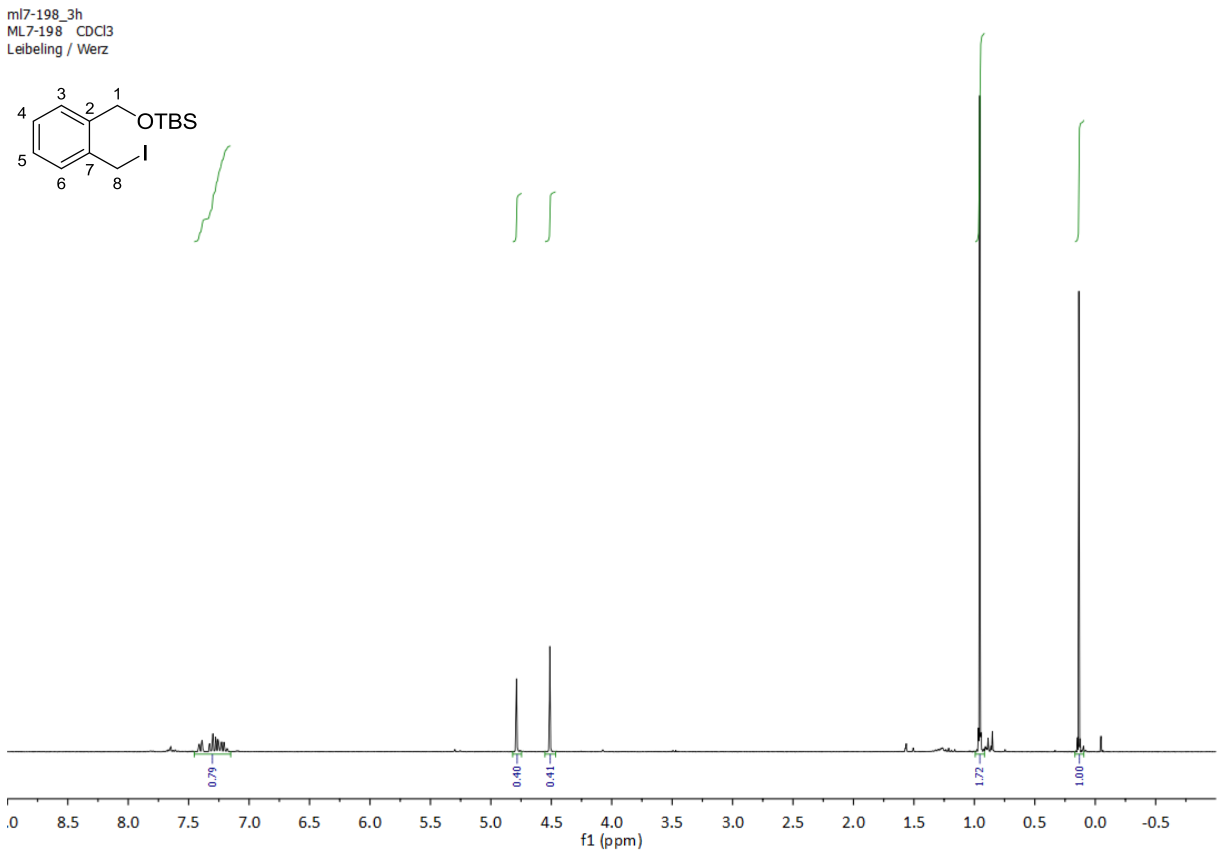

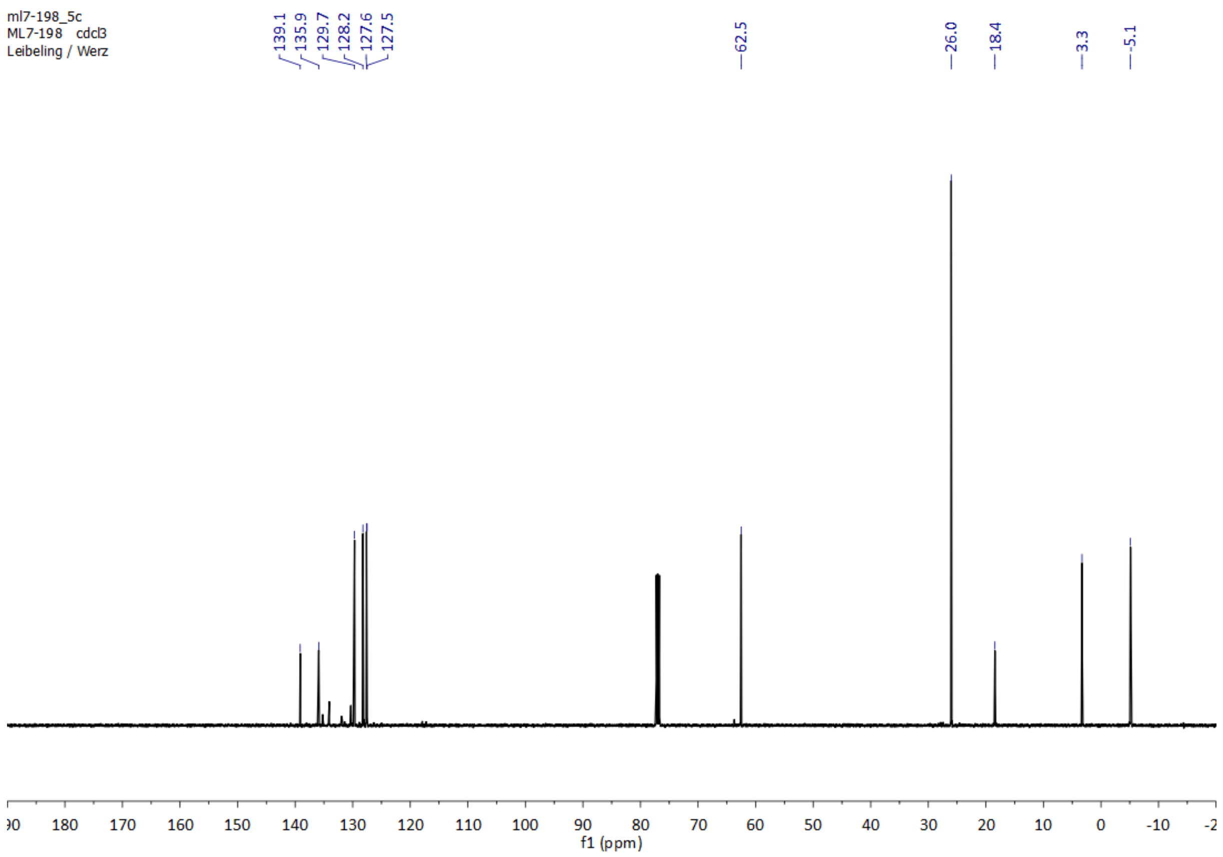

Compound 22a

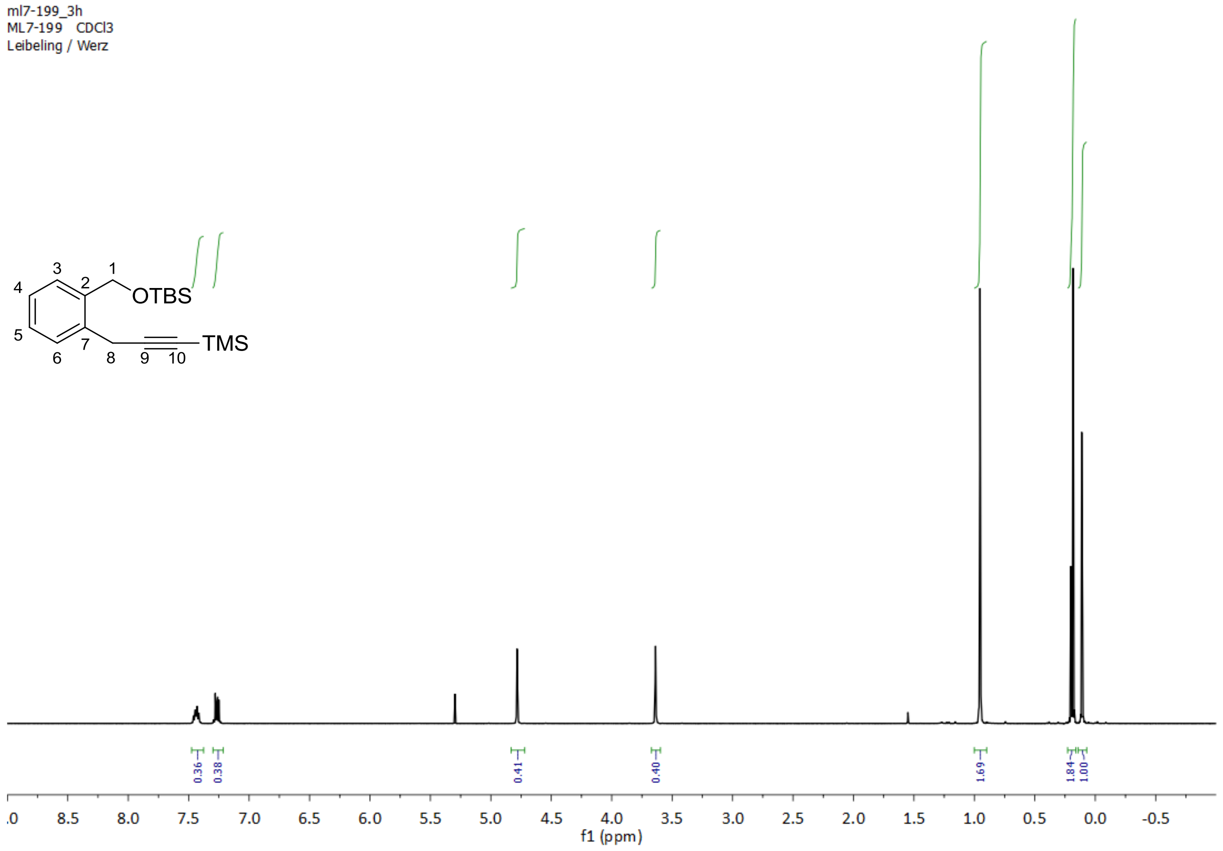

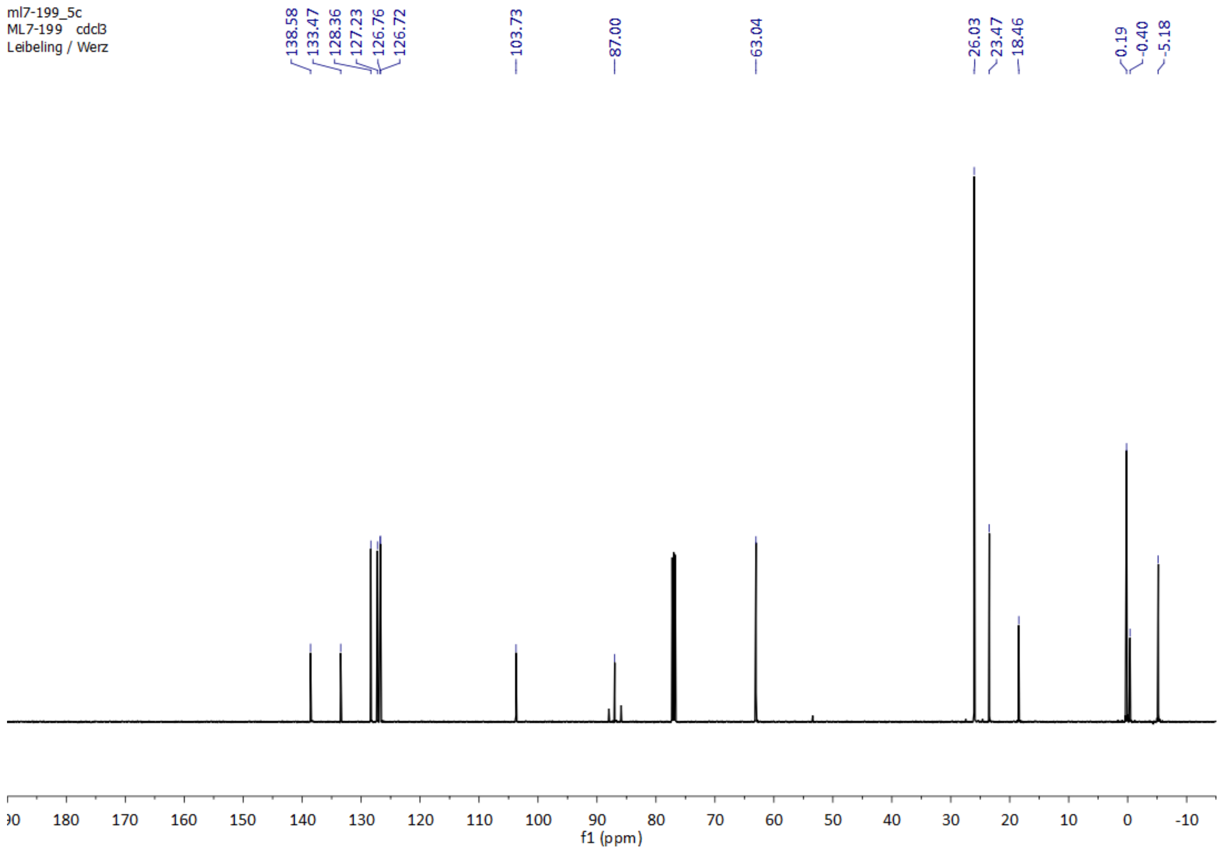

Compound 23a

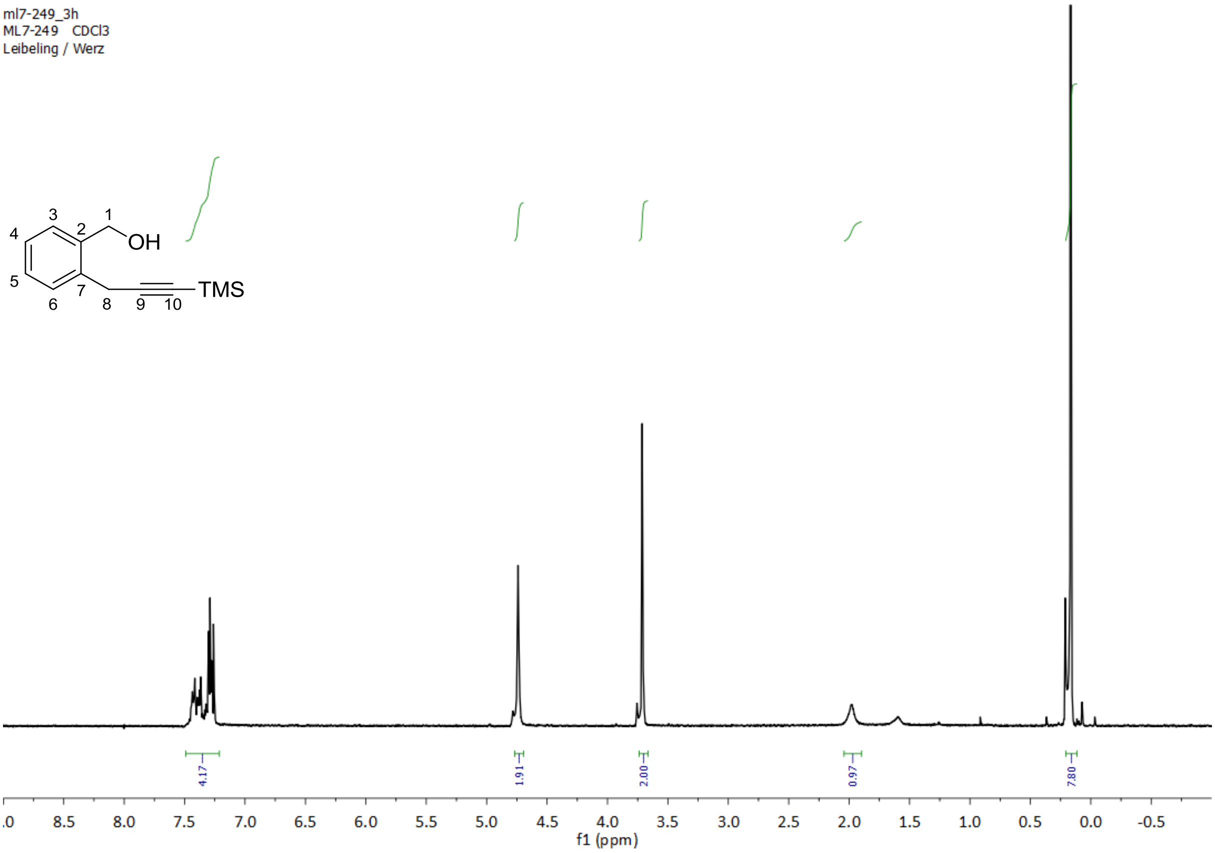

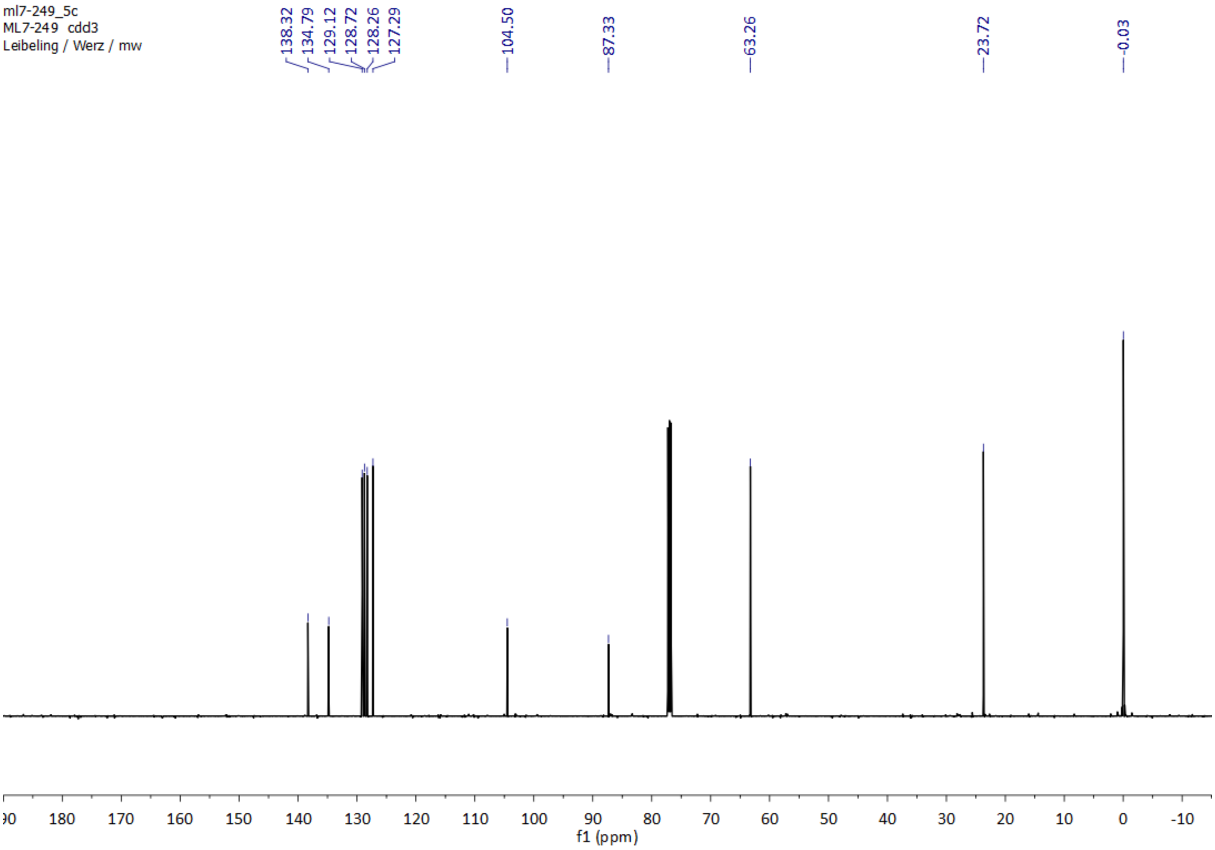

Compound 24a

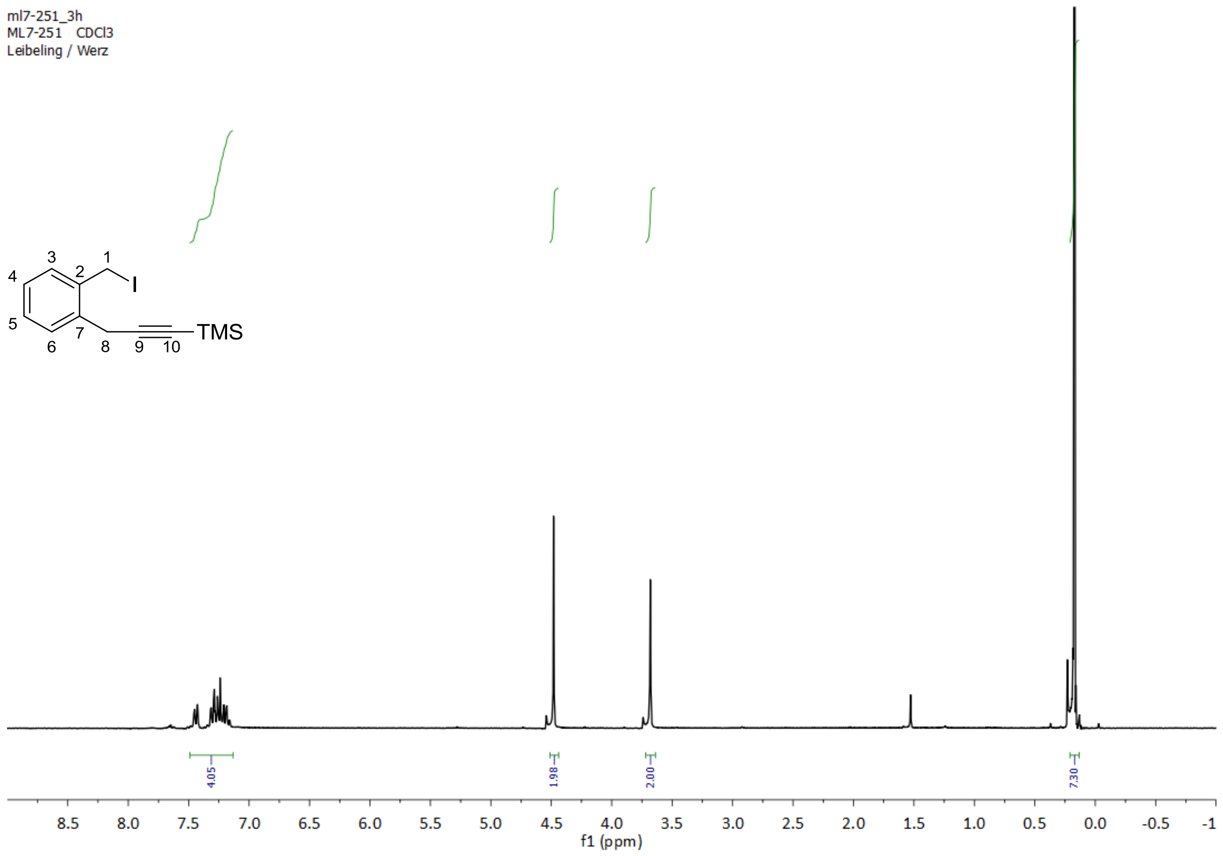

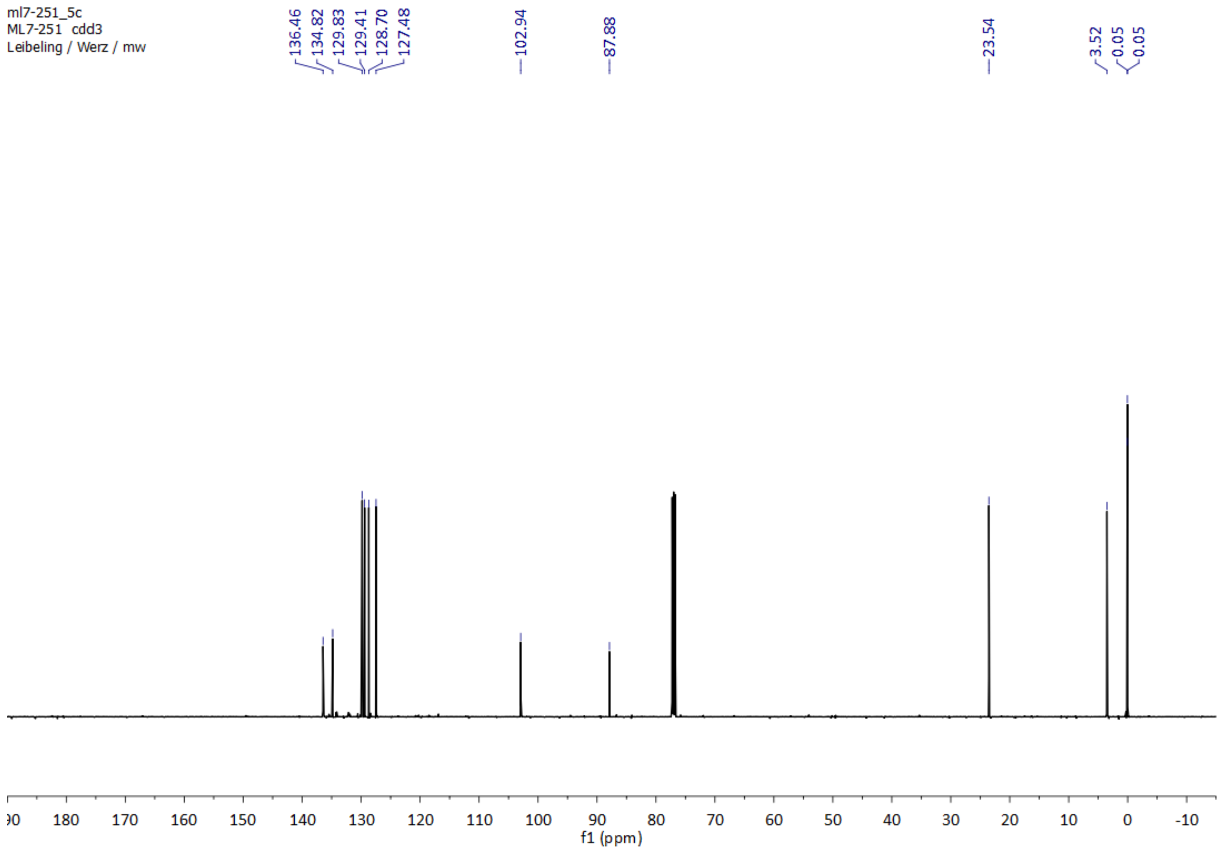

Compound 16a

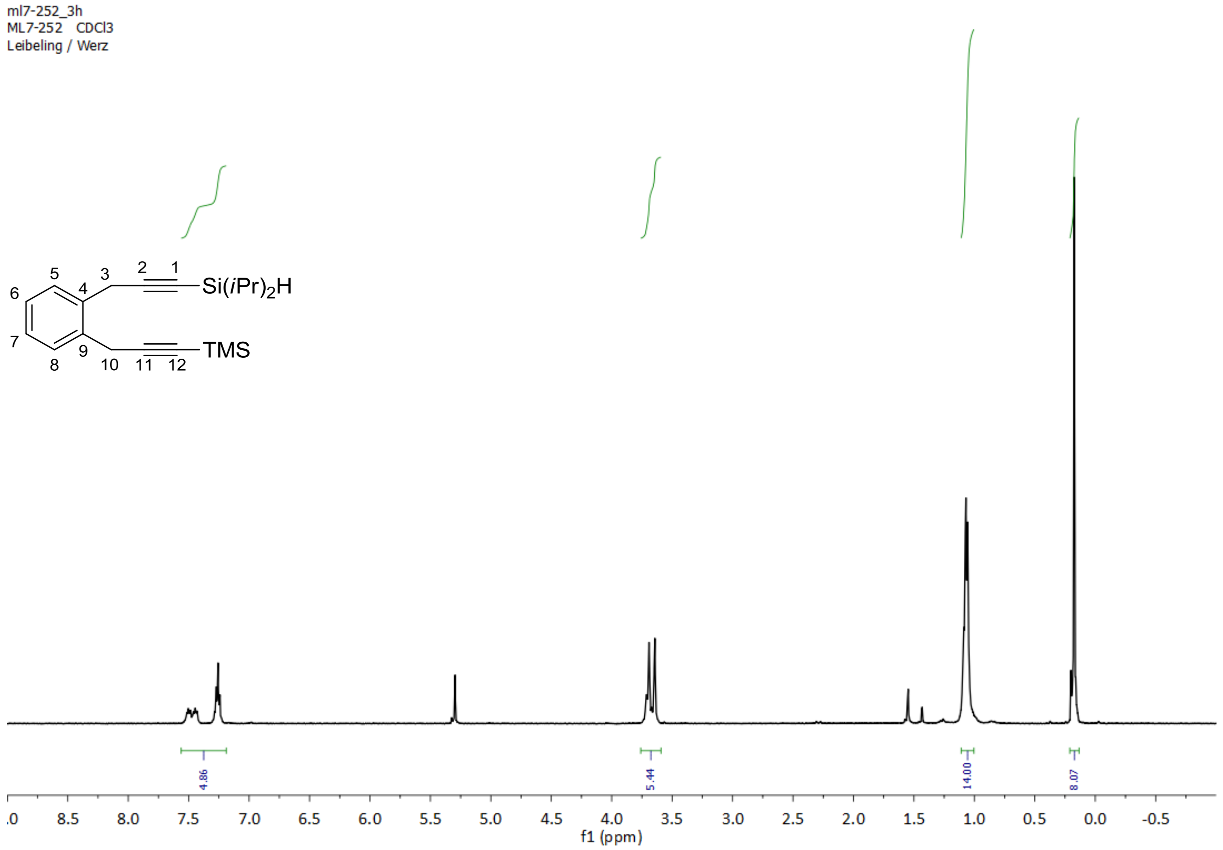

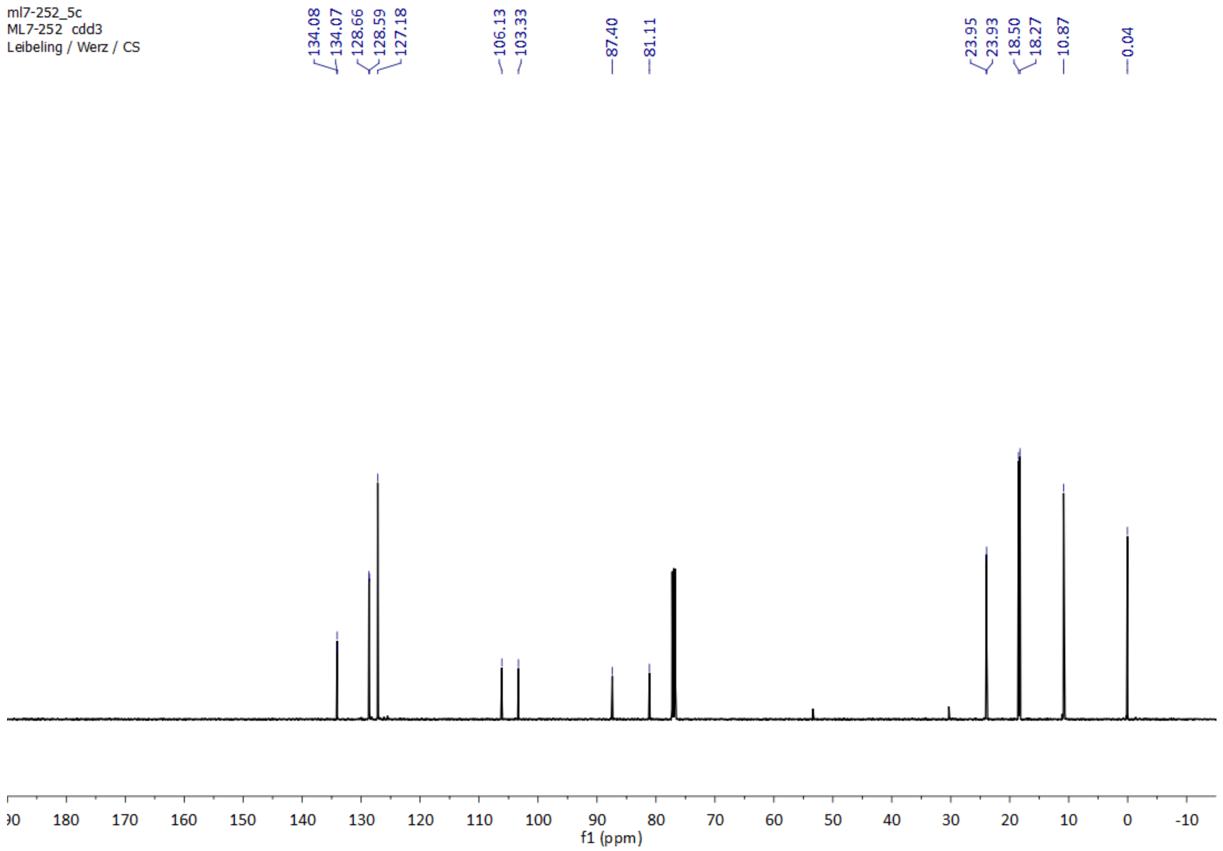

Compound 14a

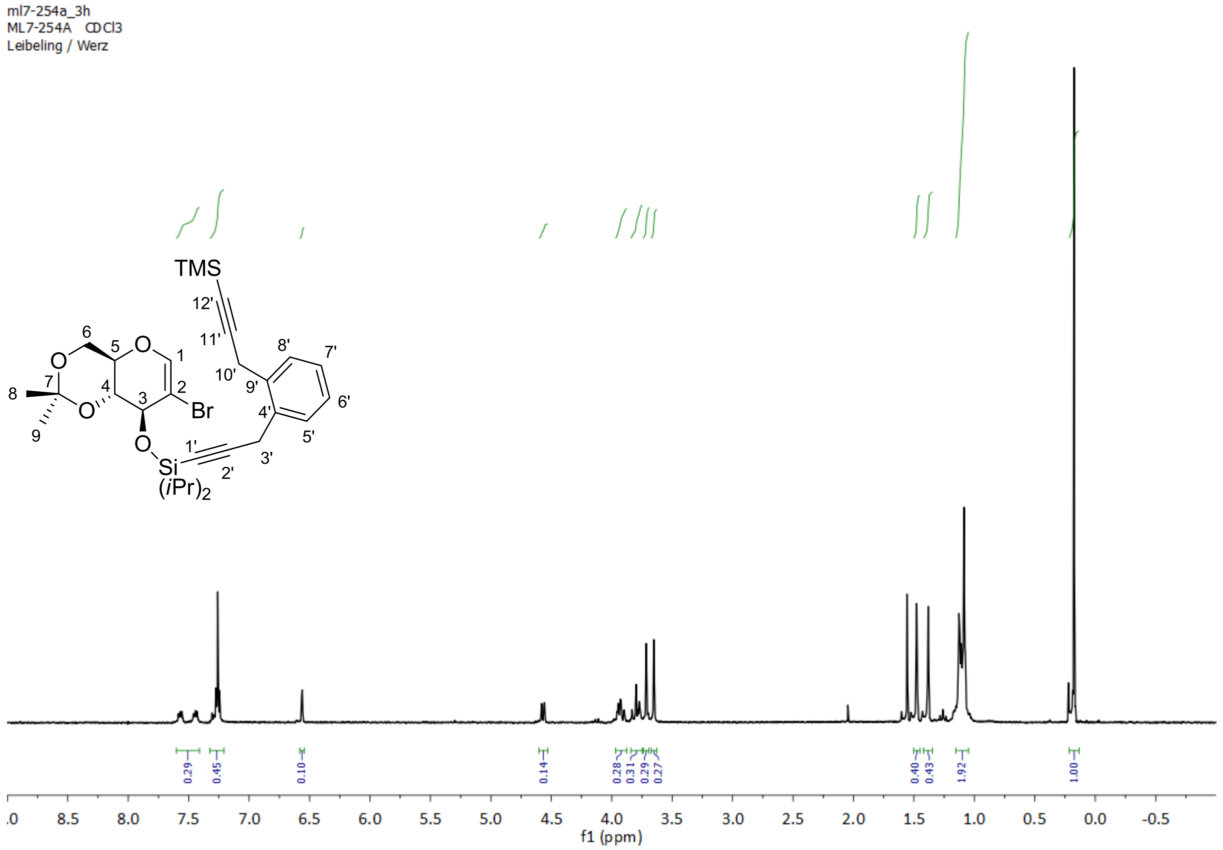

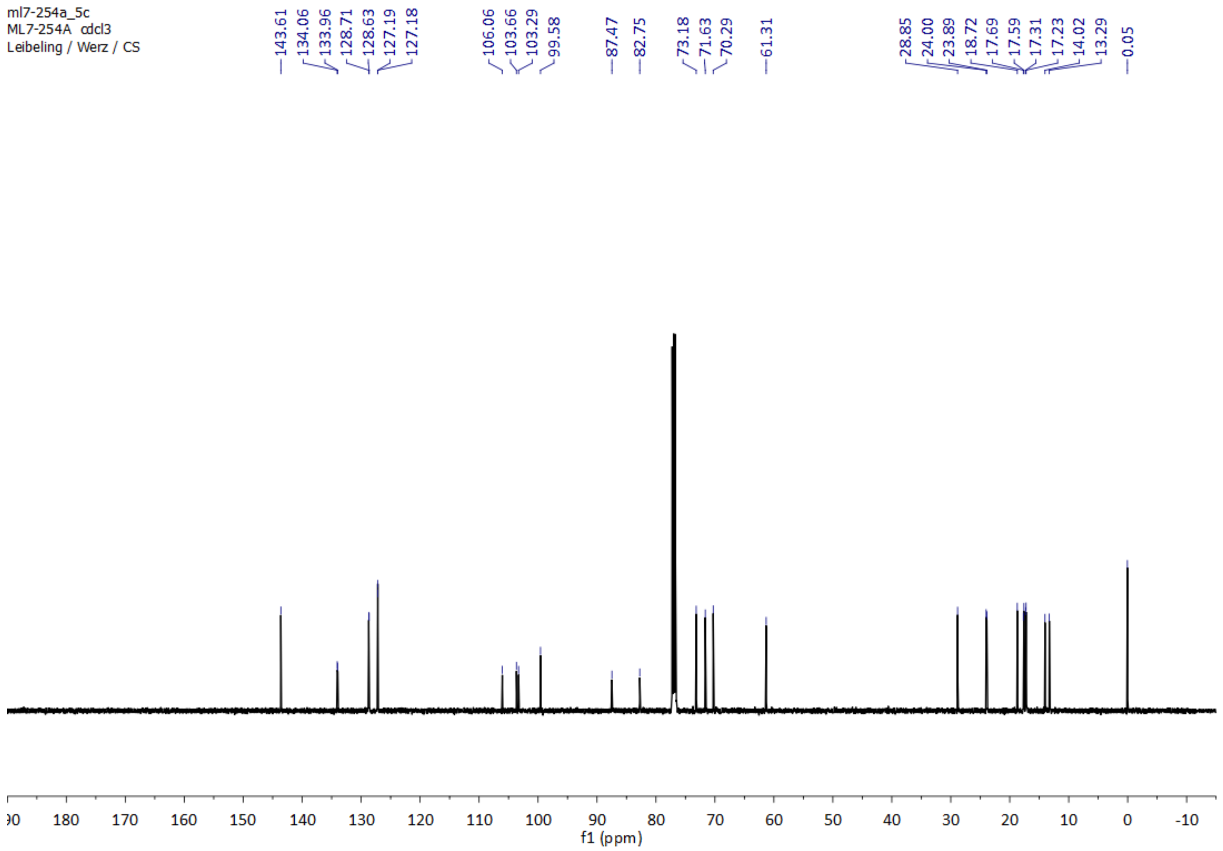

Compound 13a

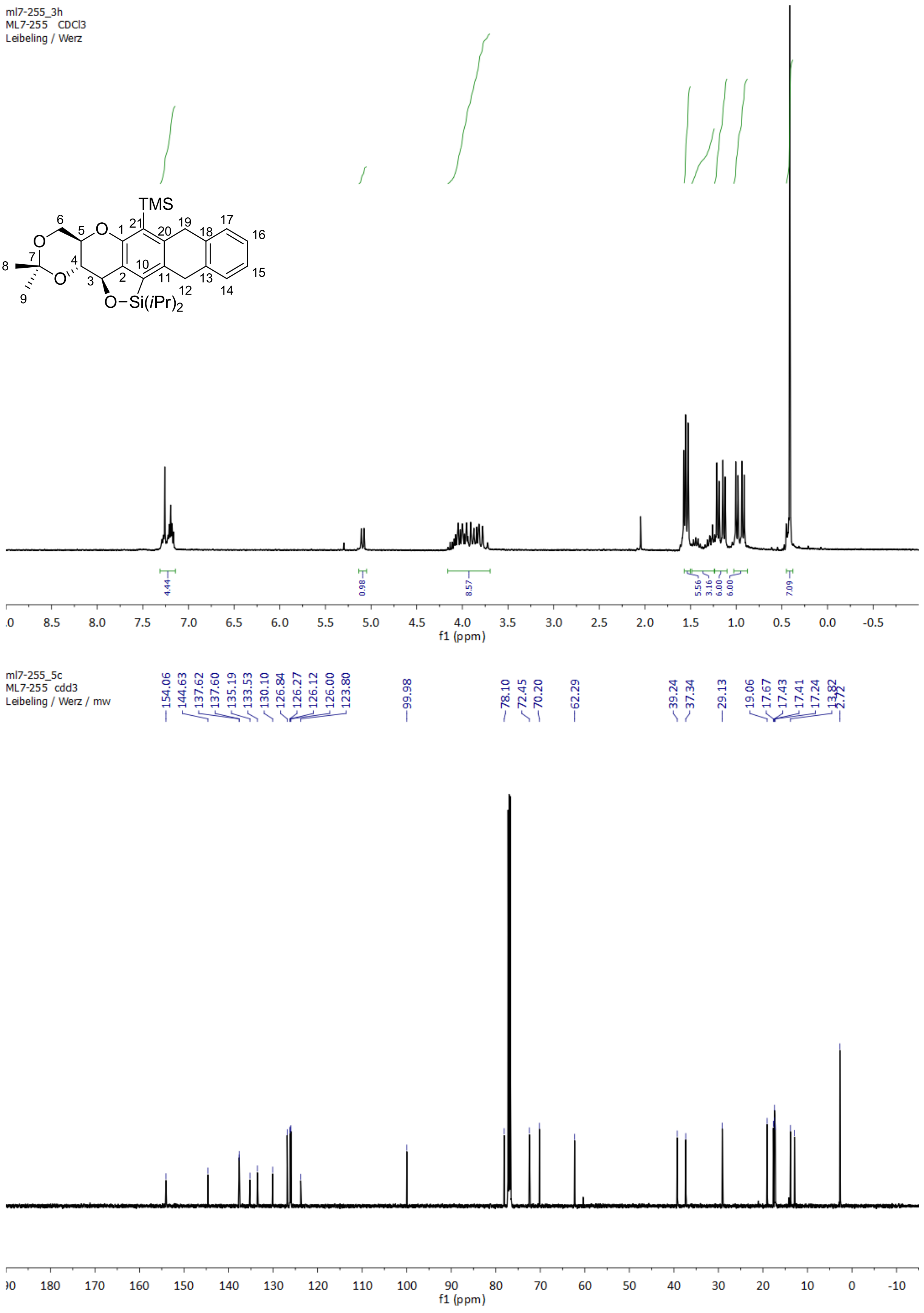

Compound 26a

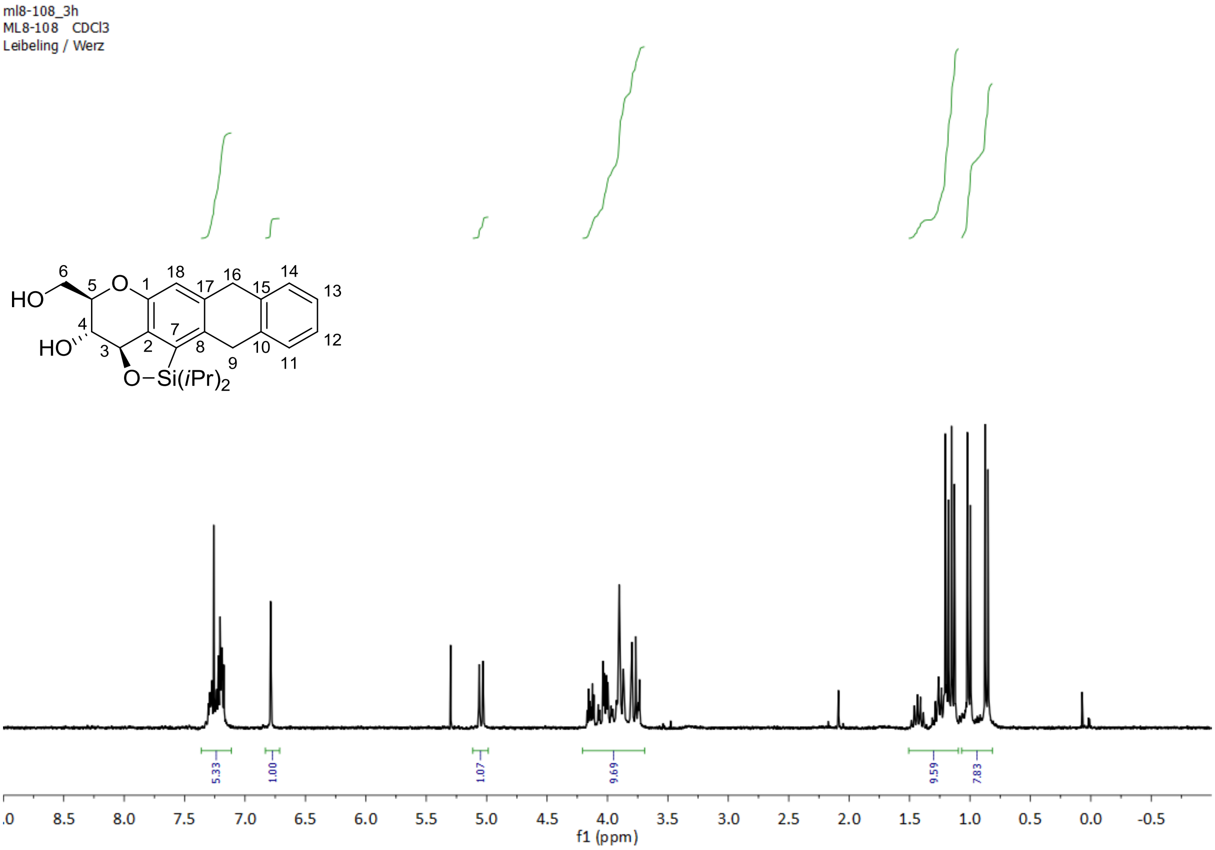

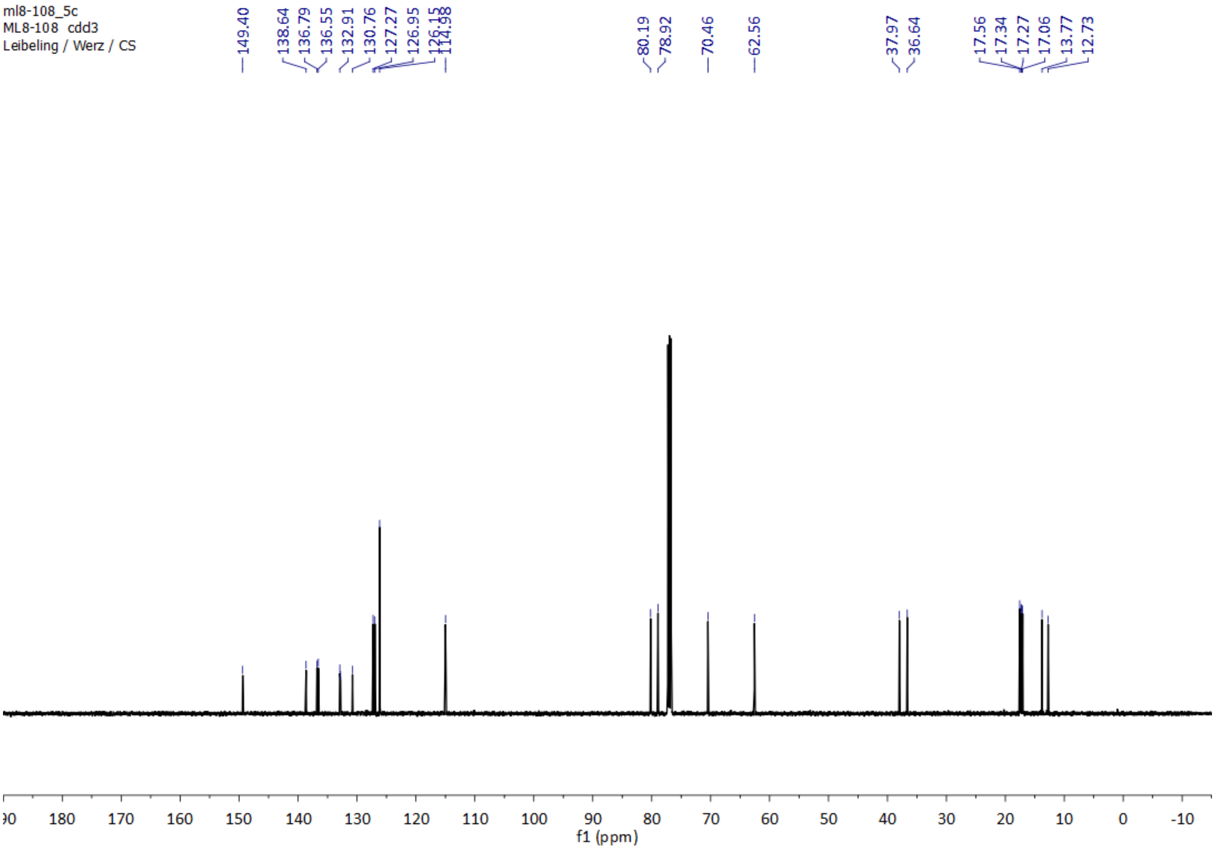

Compound 12a

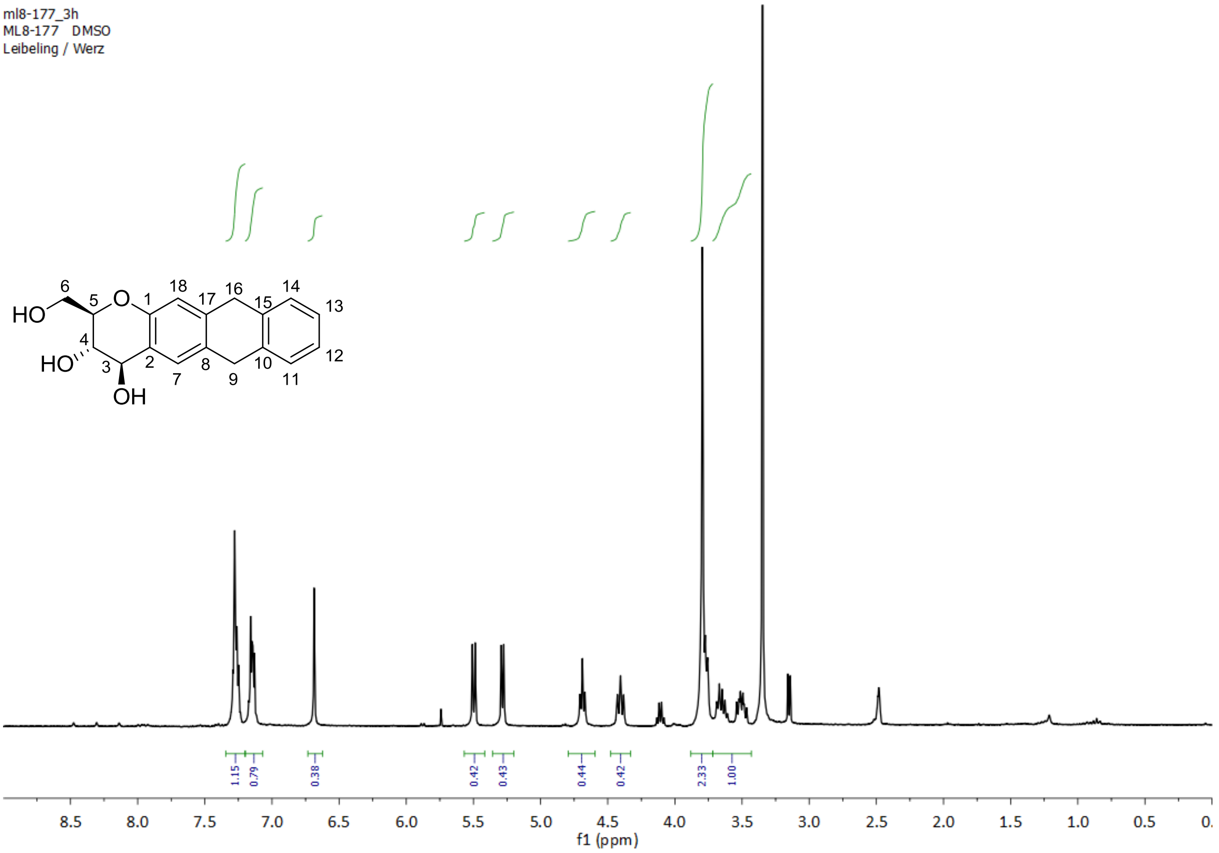

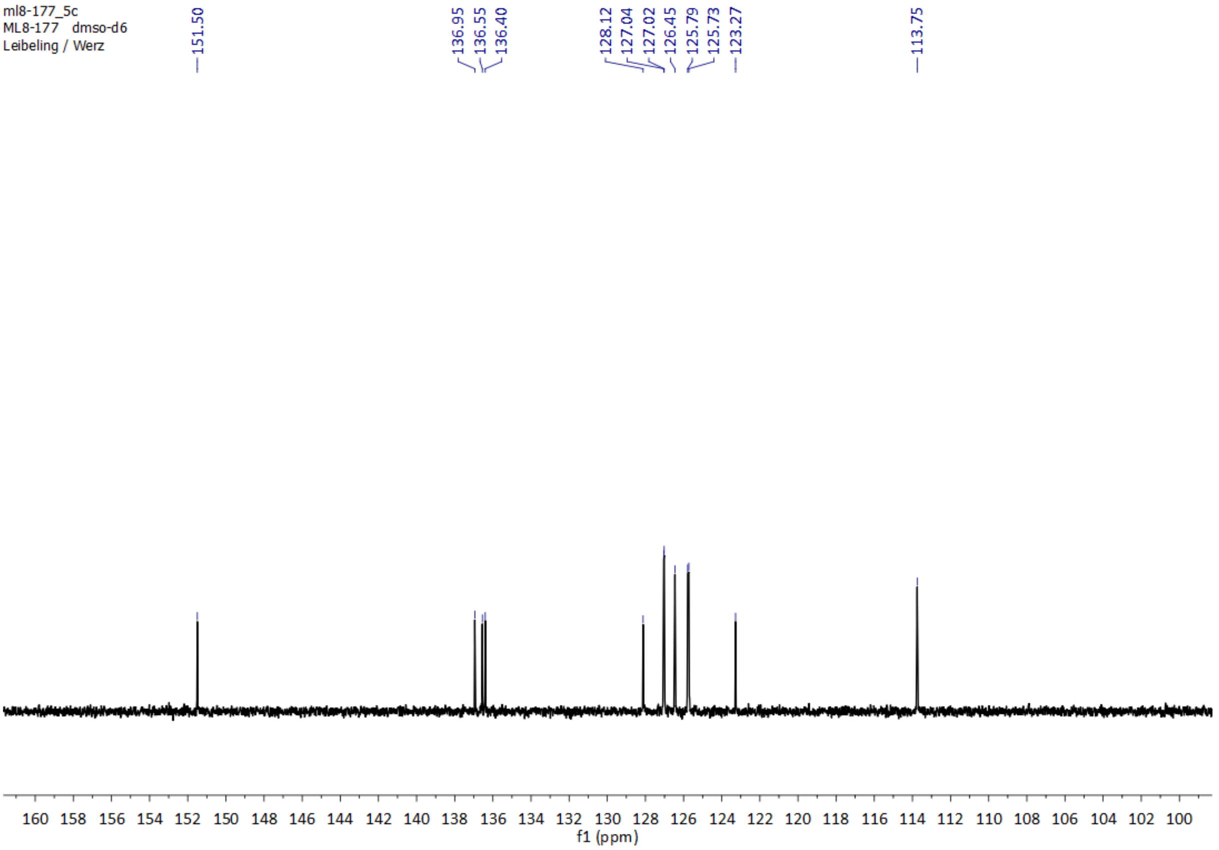

Compound 27a

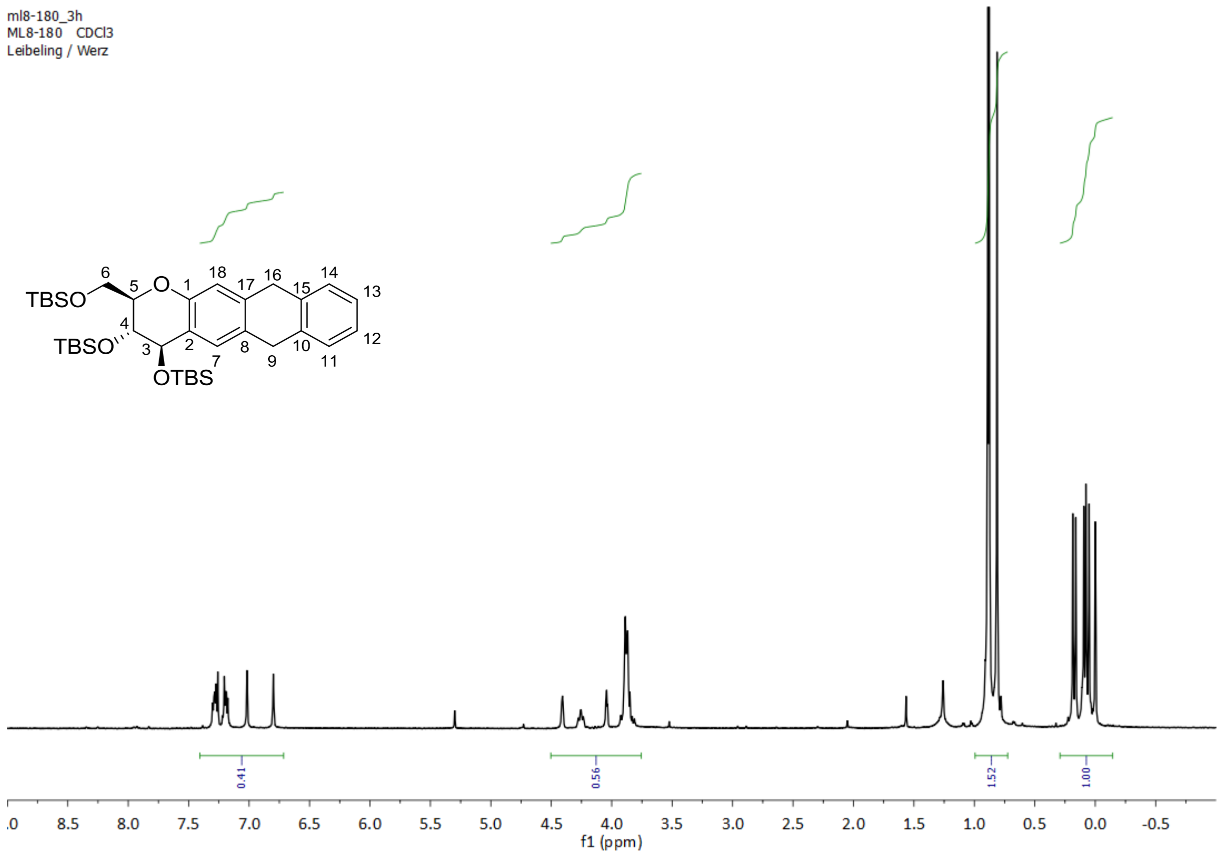

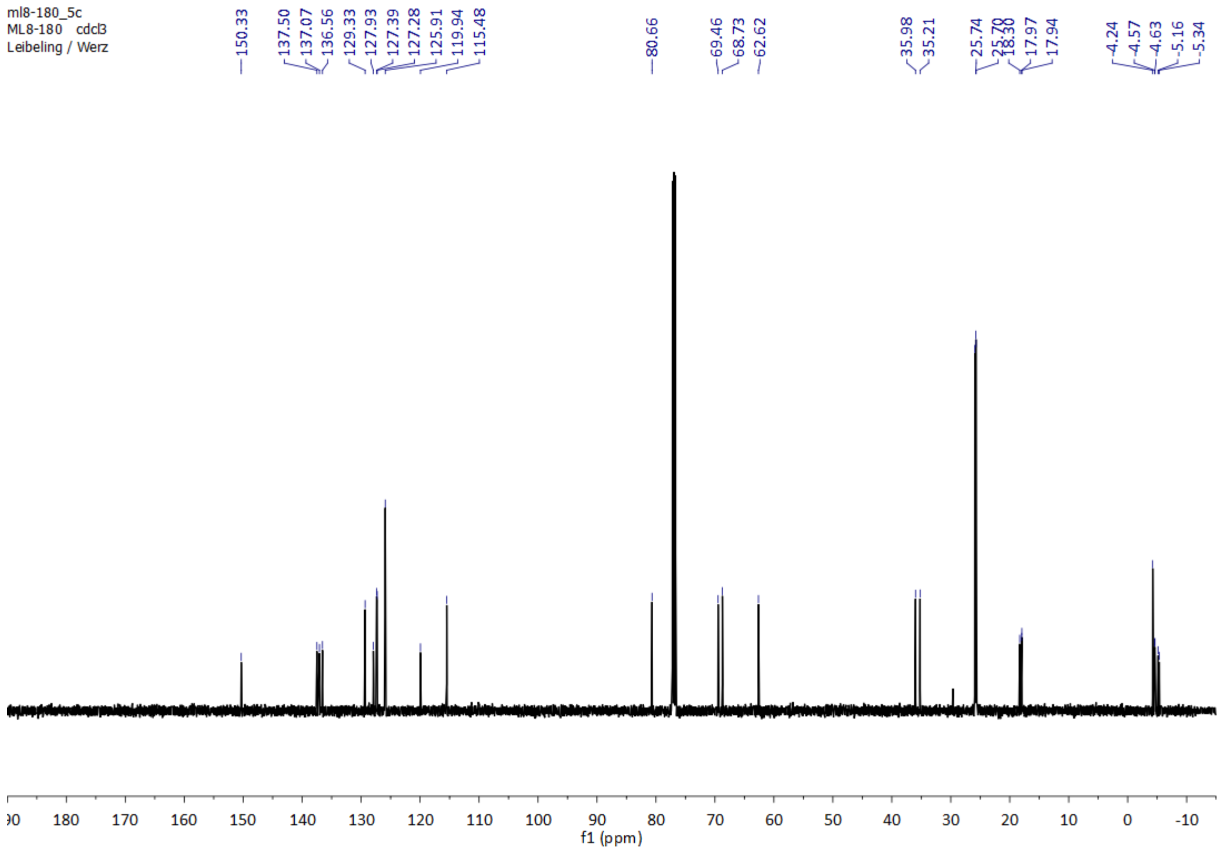

Compound 28a

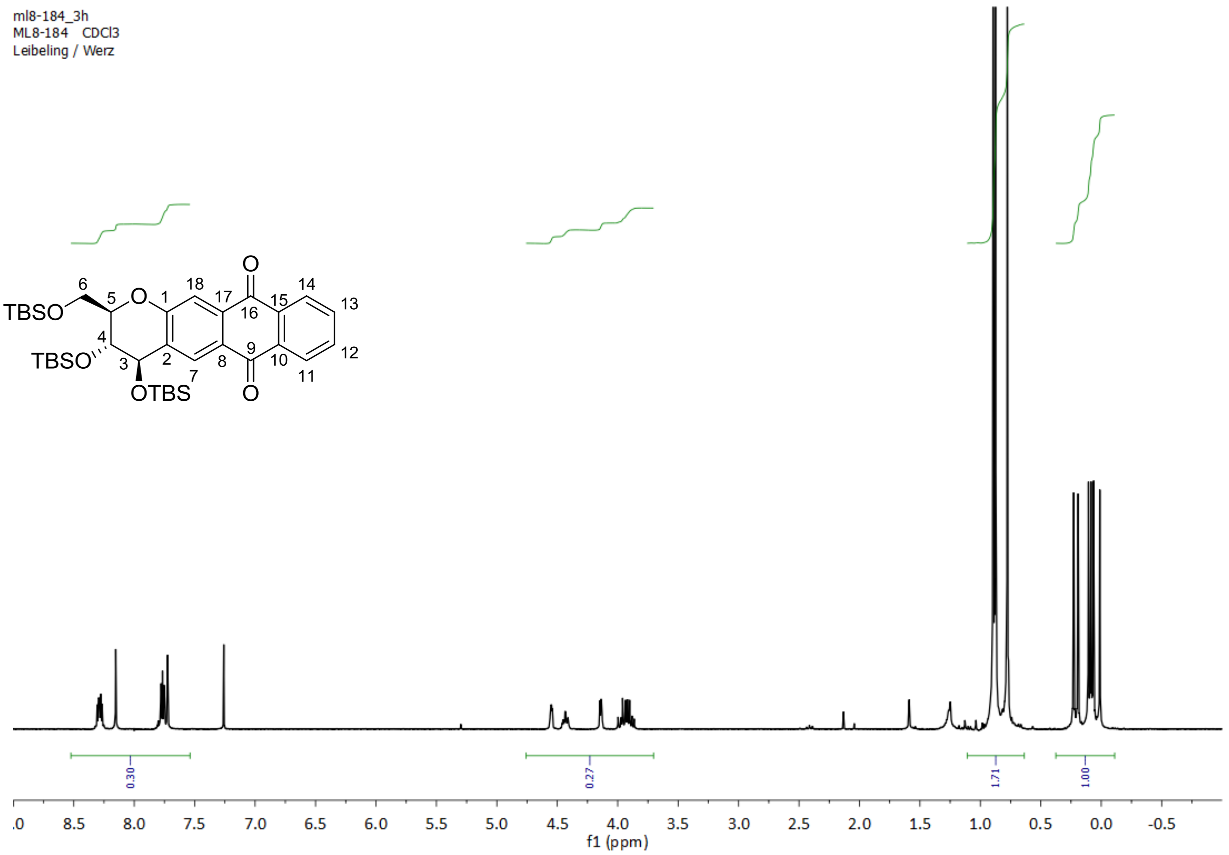

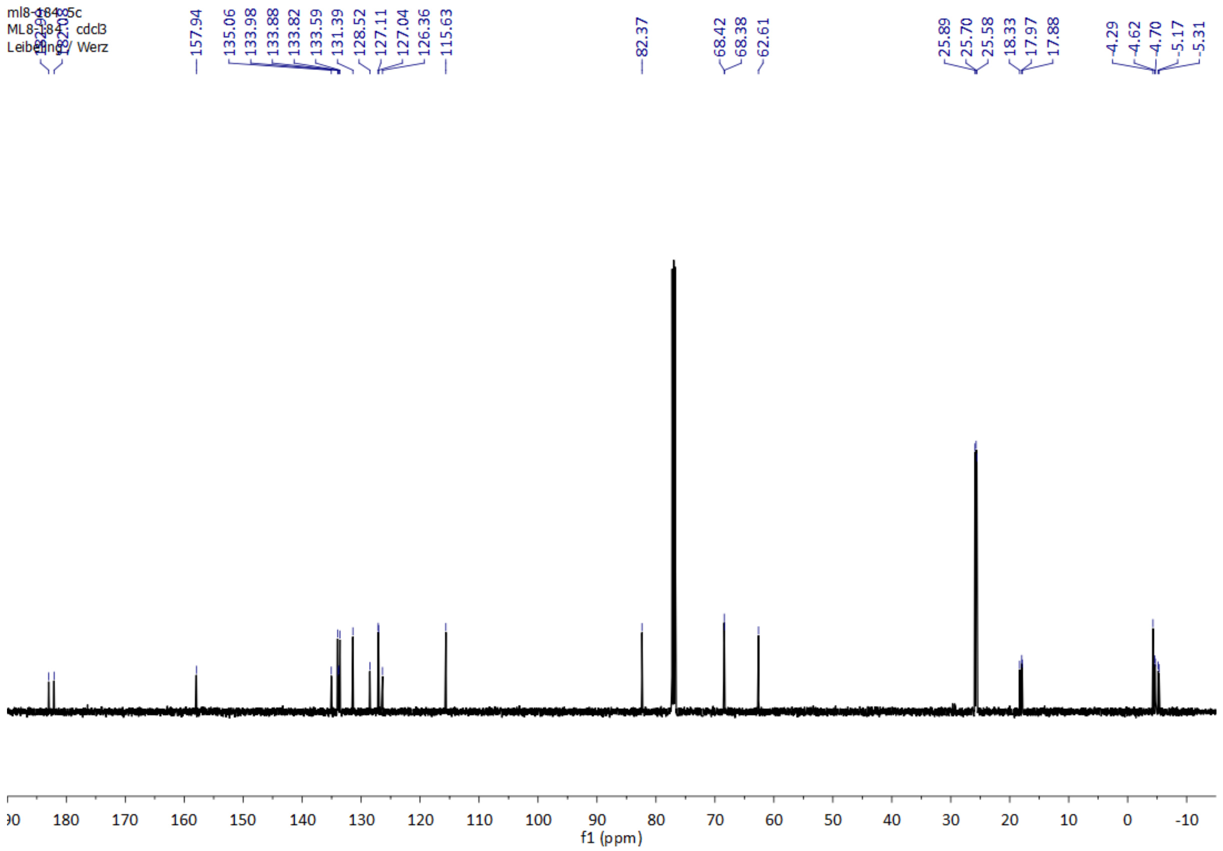

Compound 11a

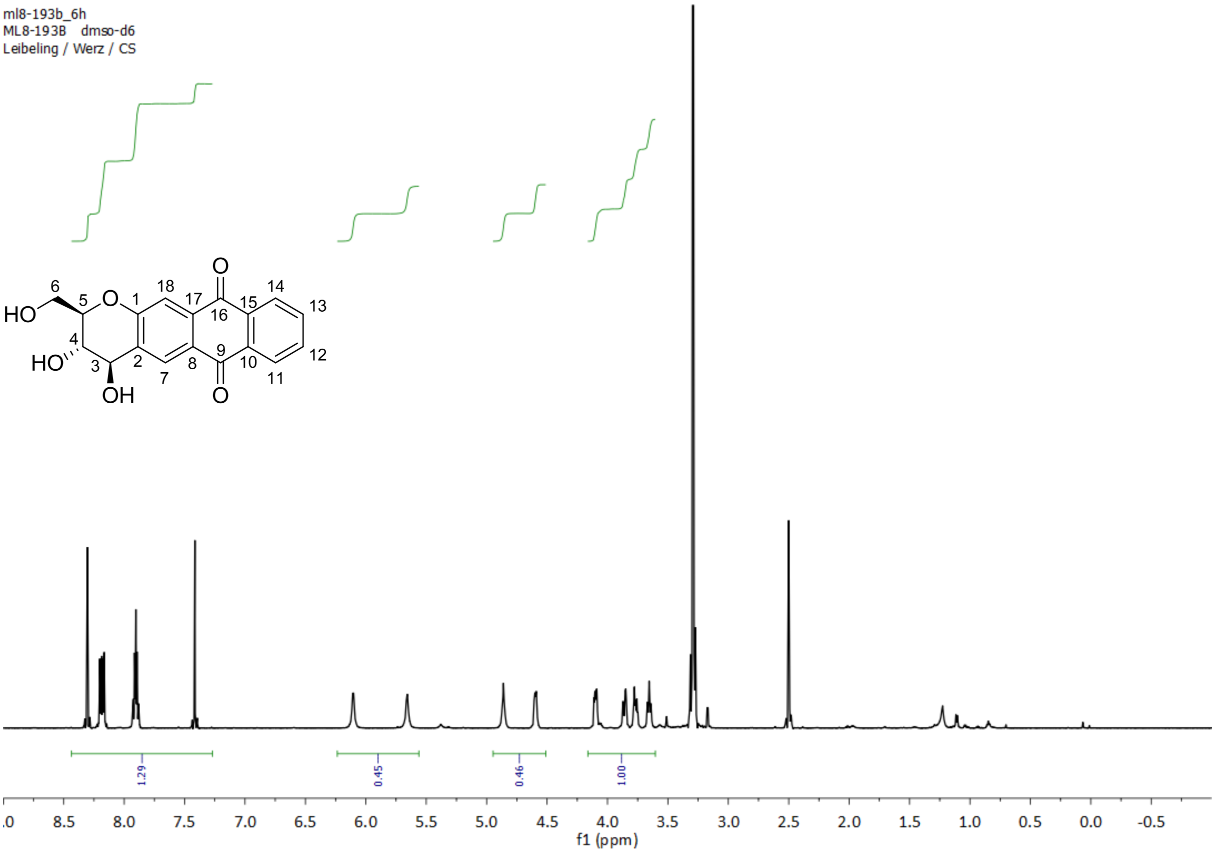

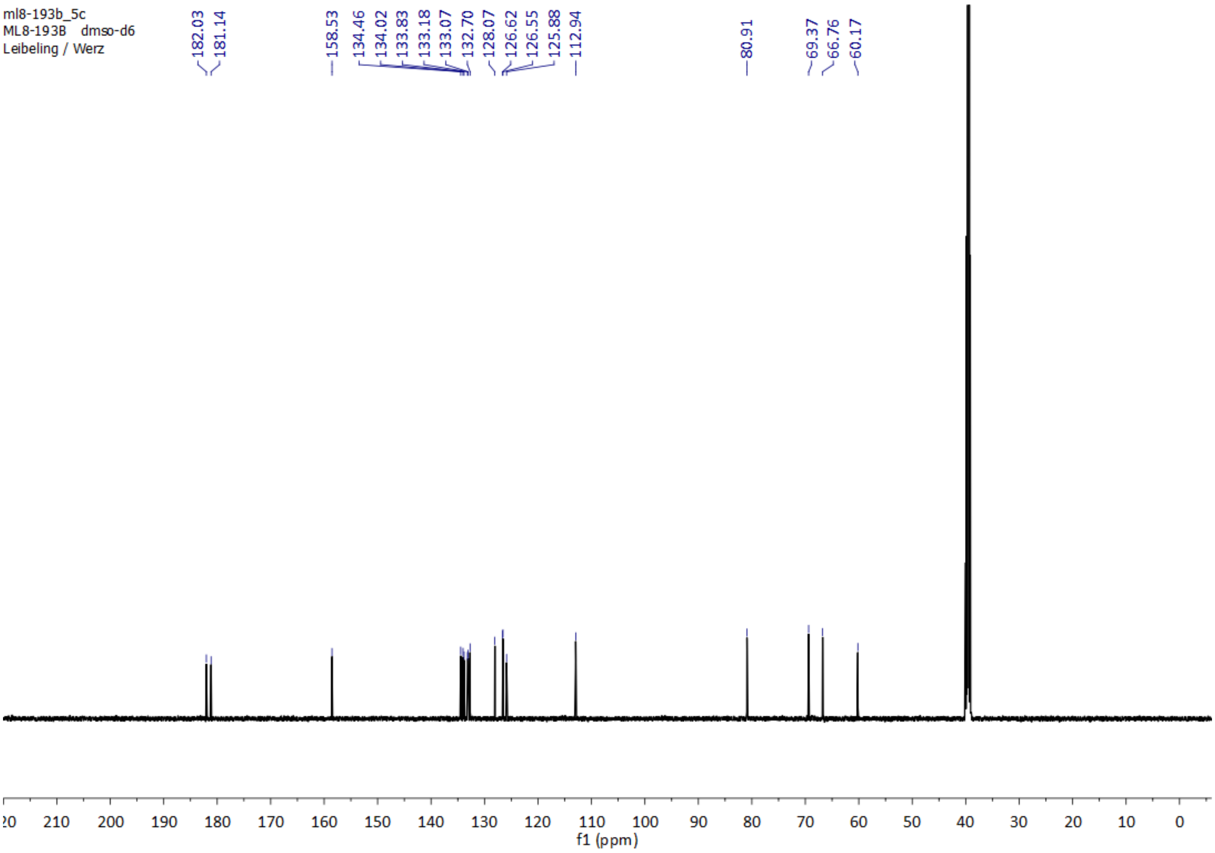

Compound 23b

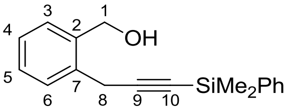

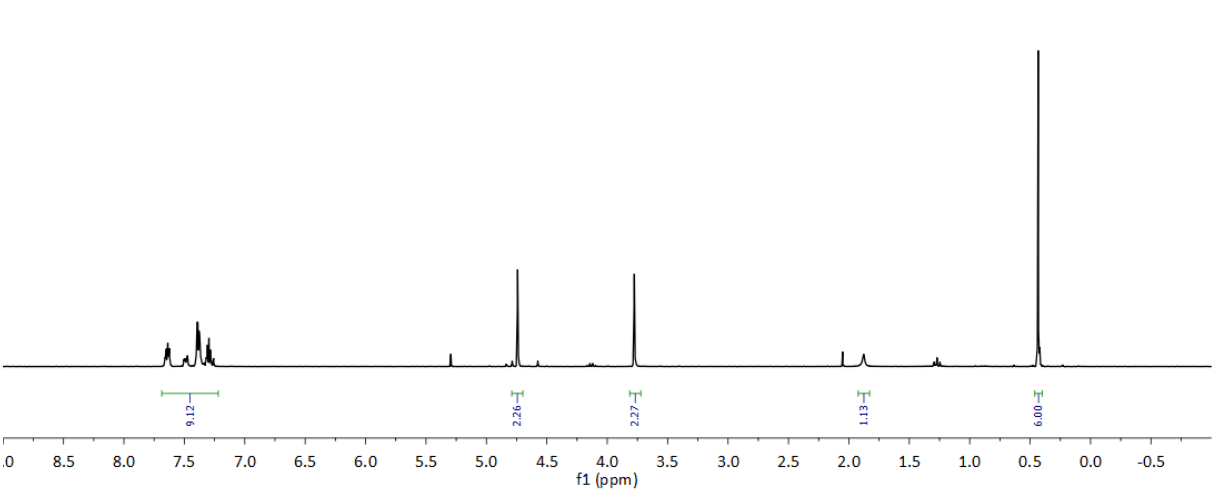

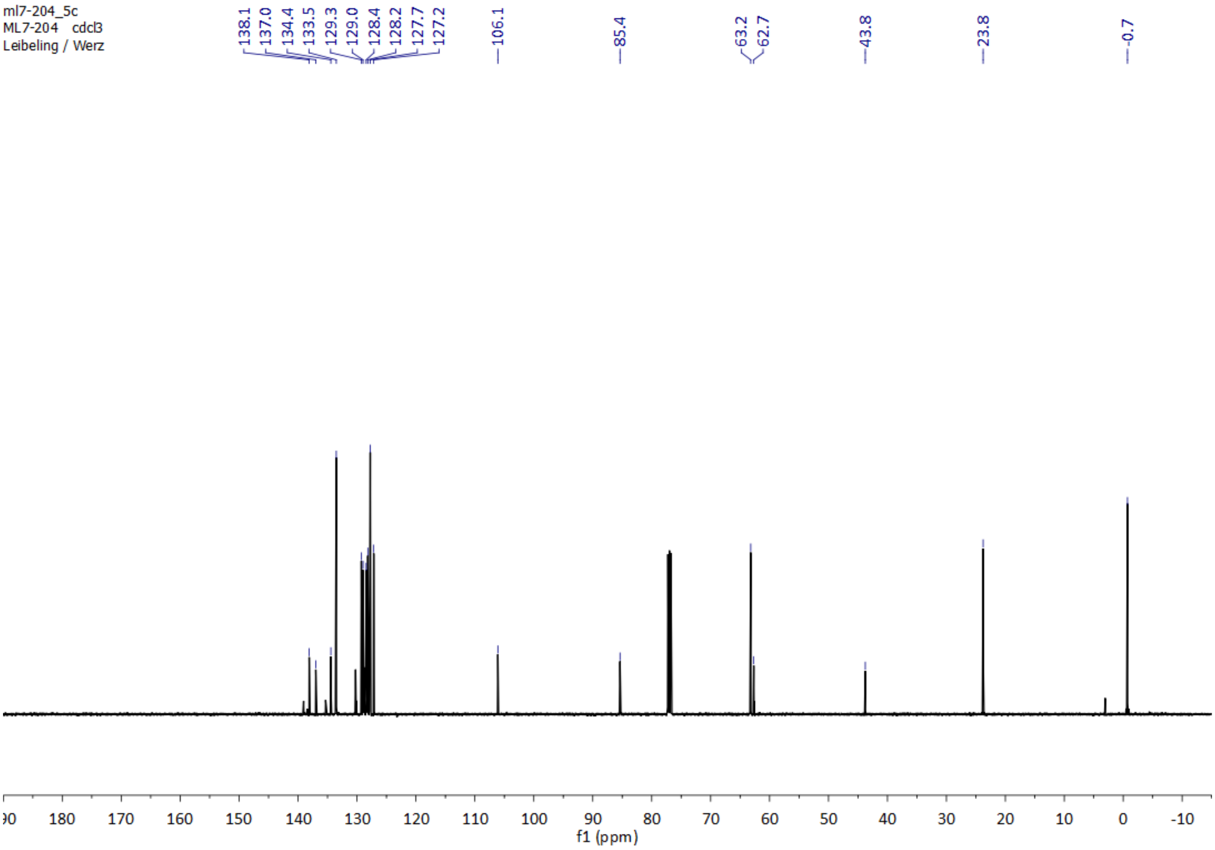

Compound 24b

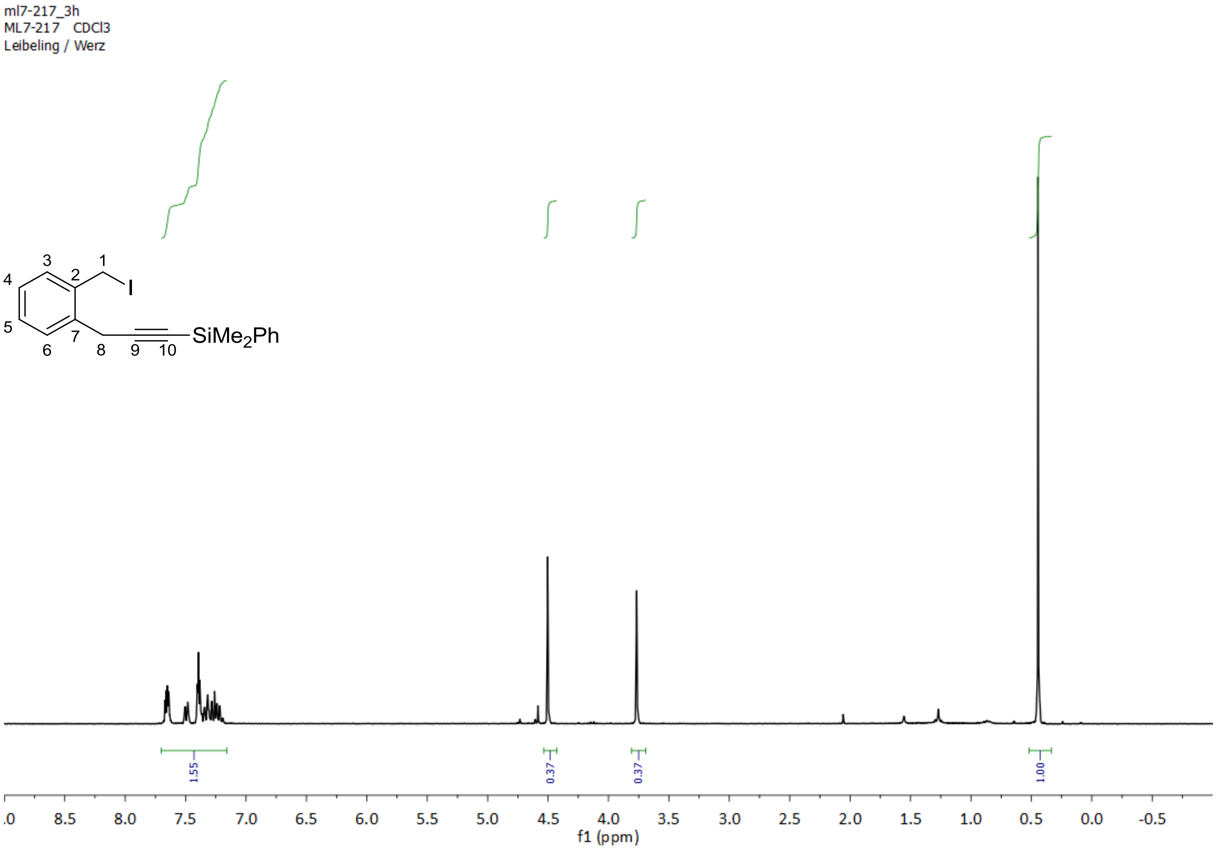

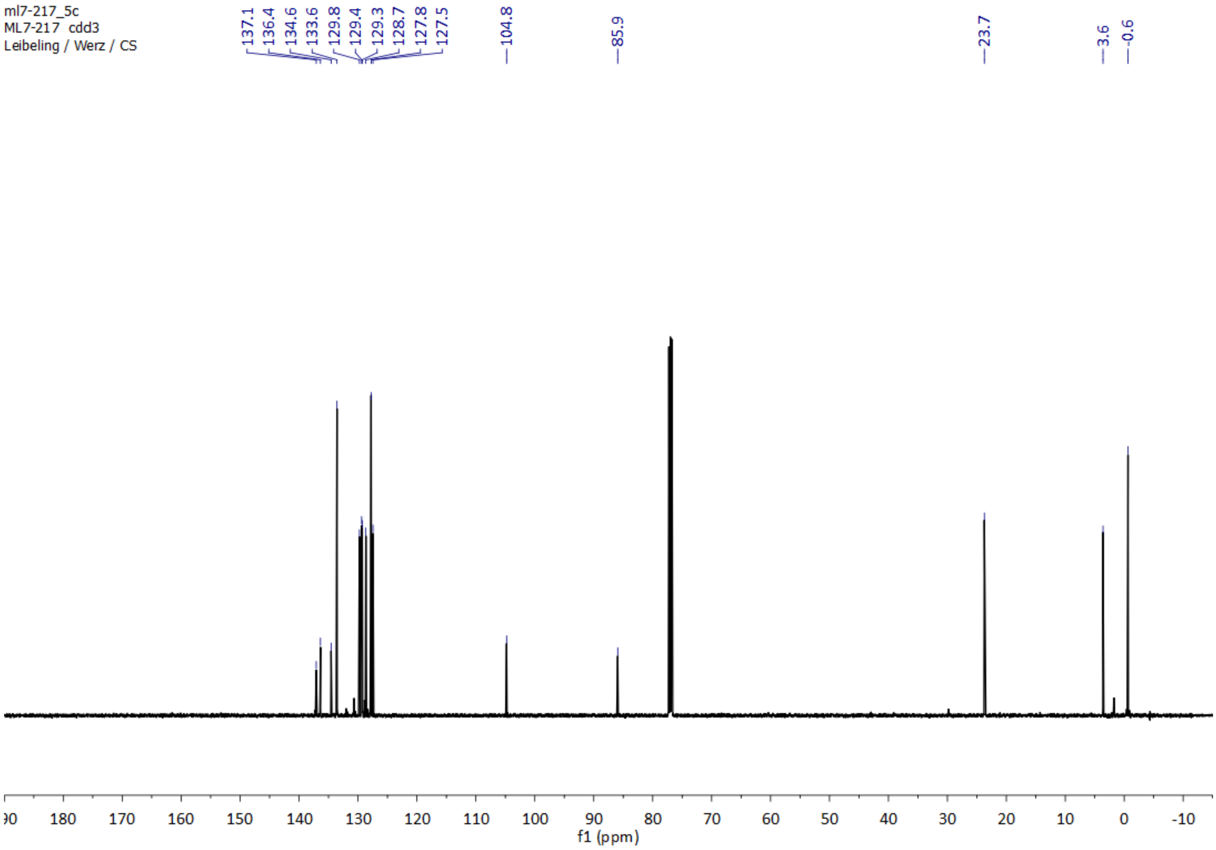

Compound 16b

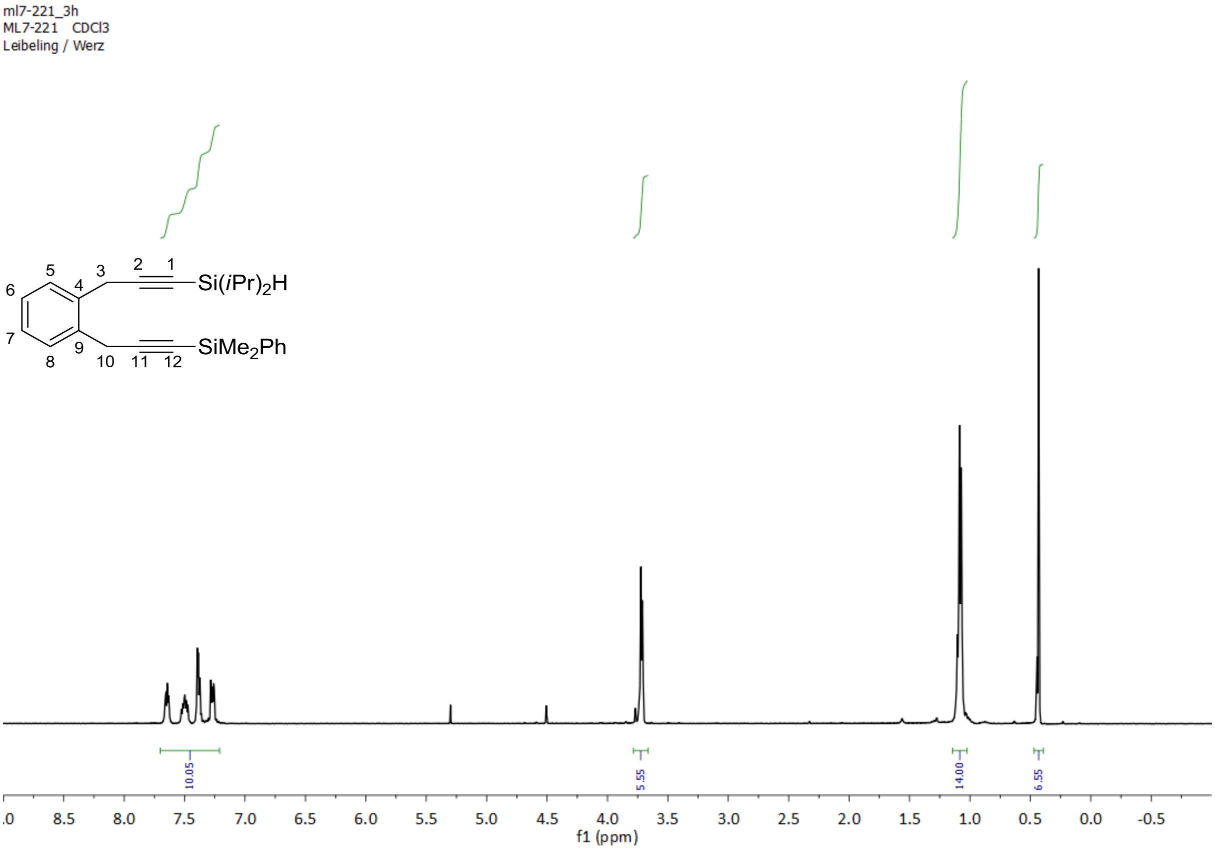

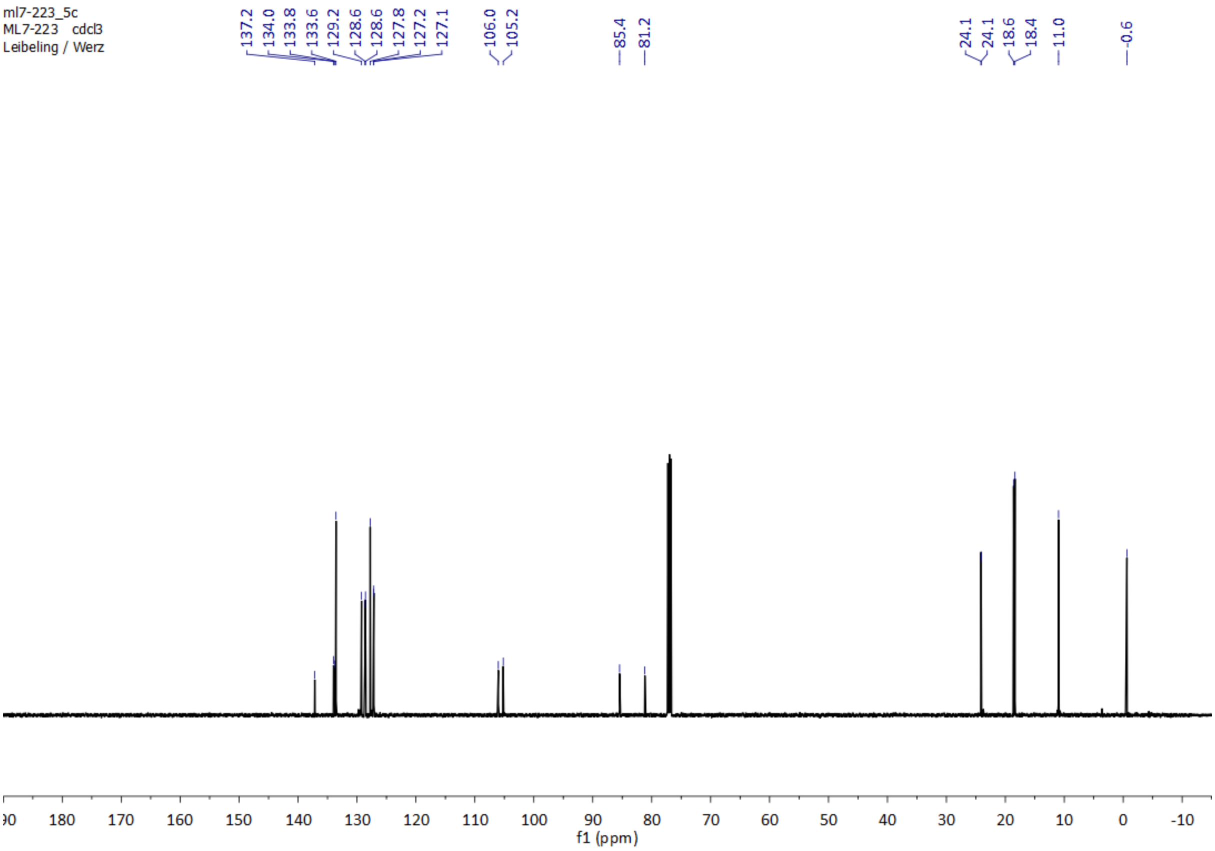

Compound 14b

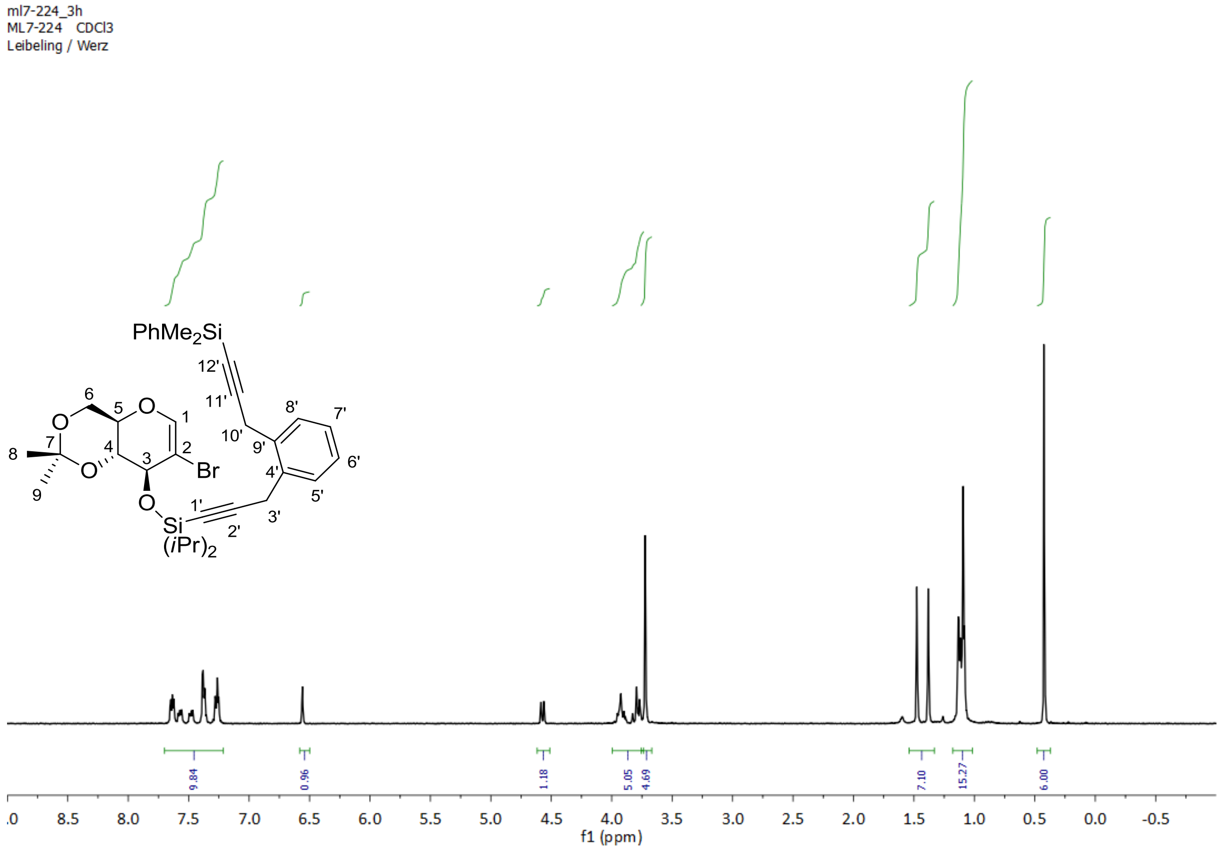

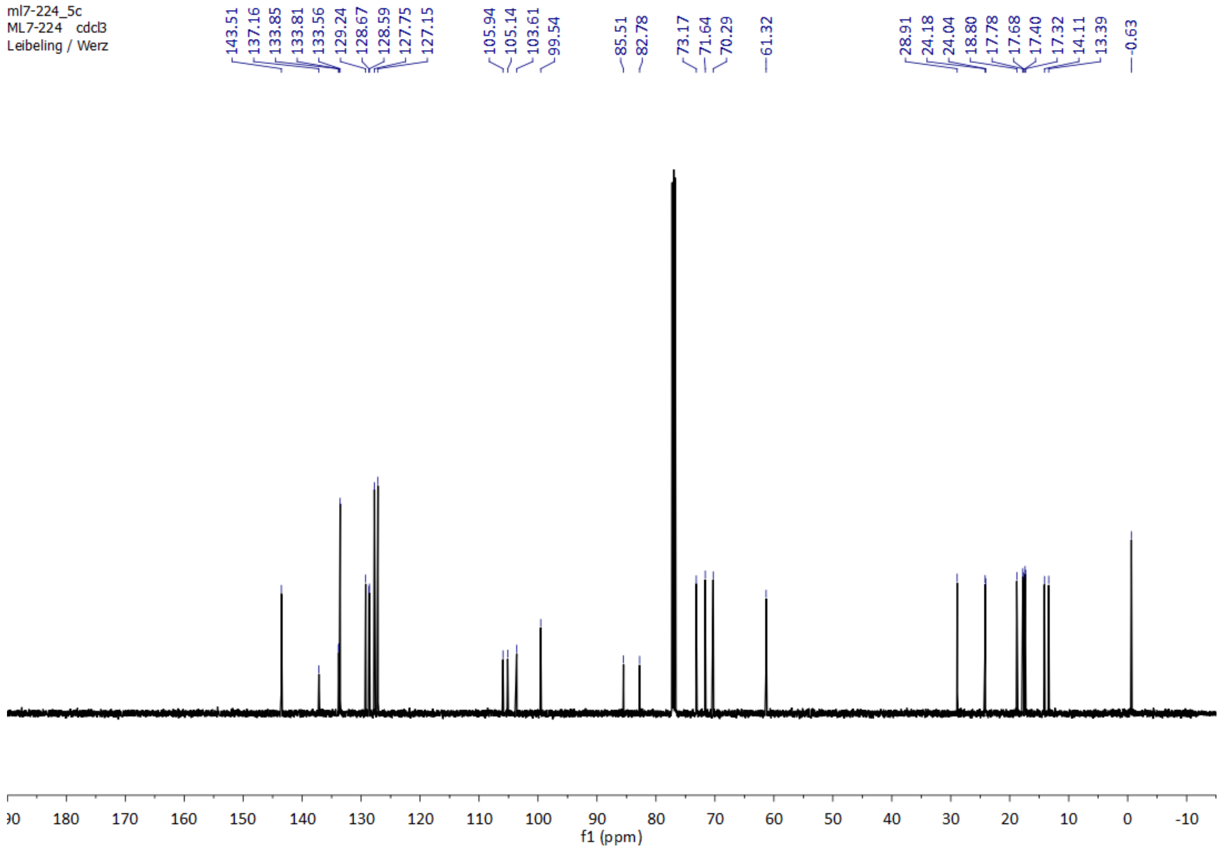

Compound 13b

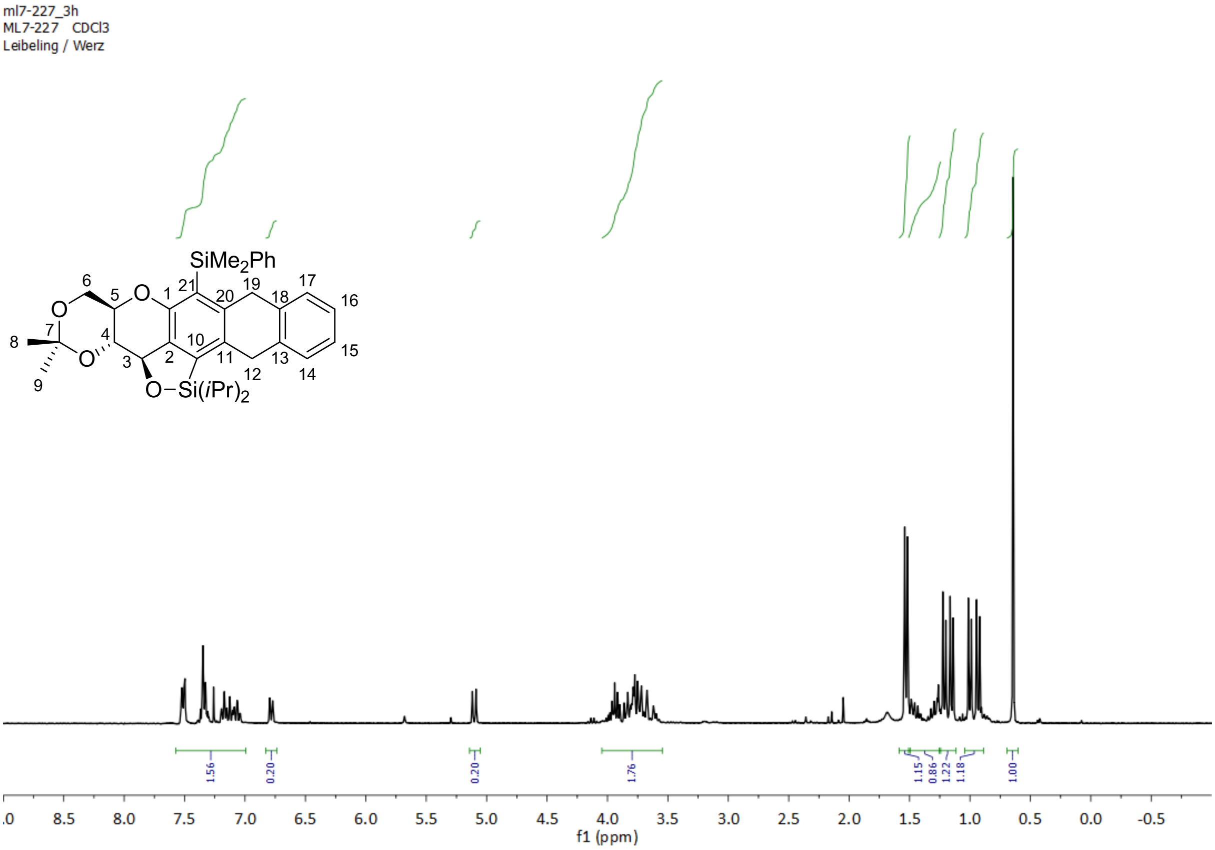

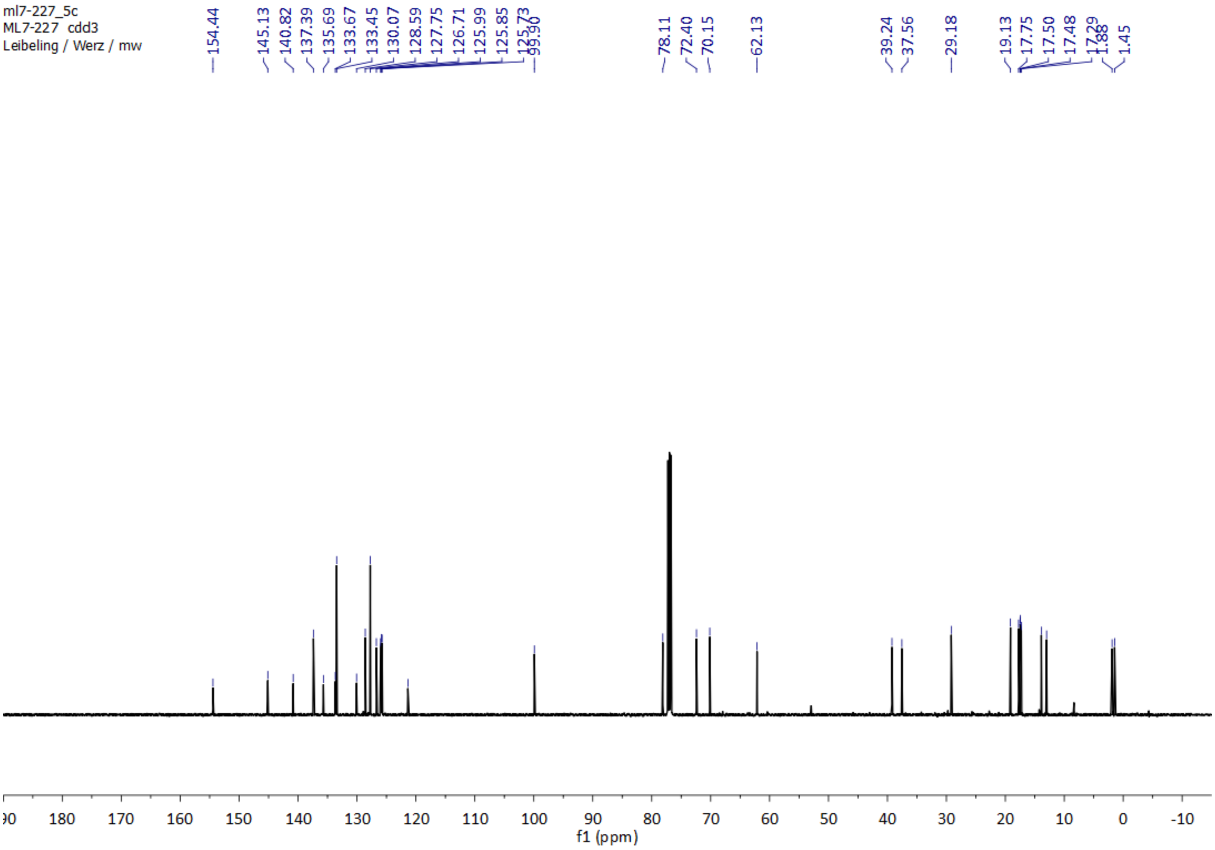

Compound 22c

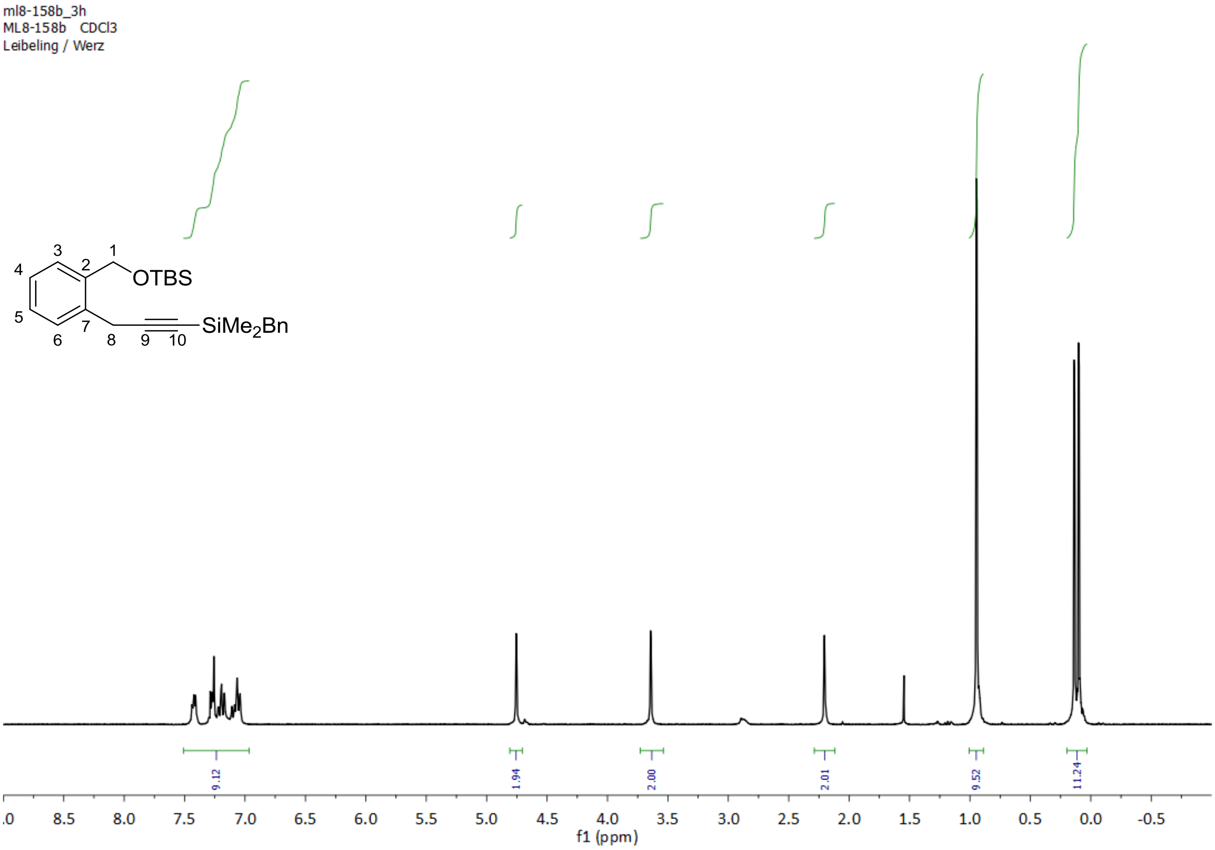

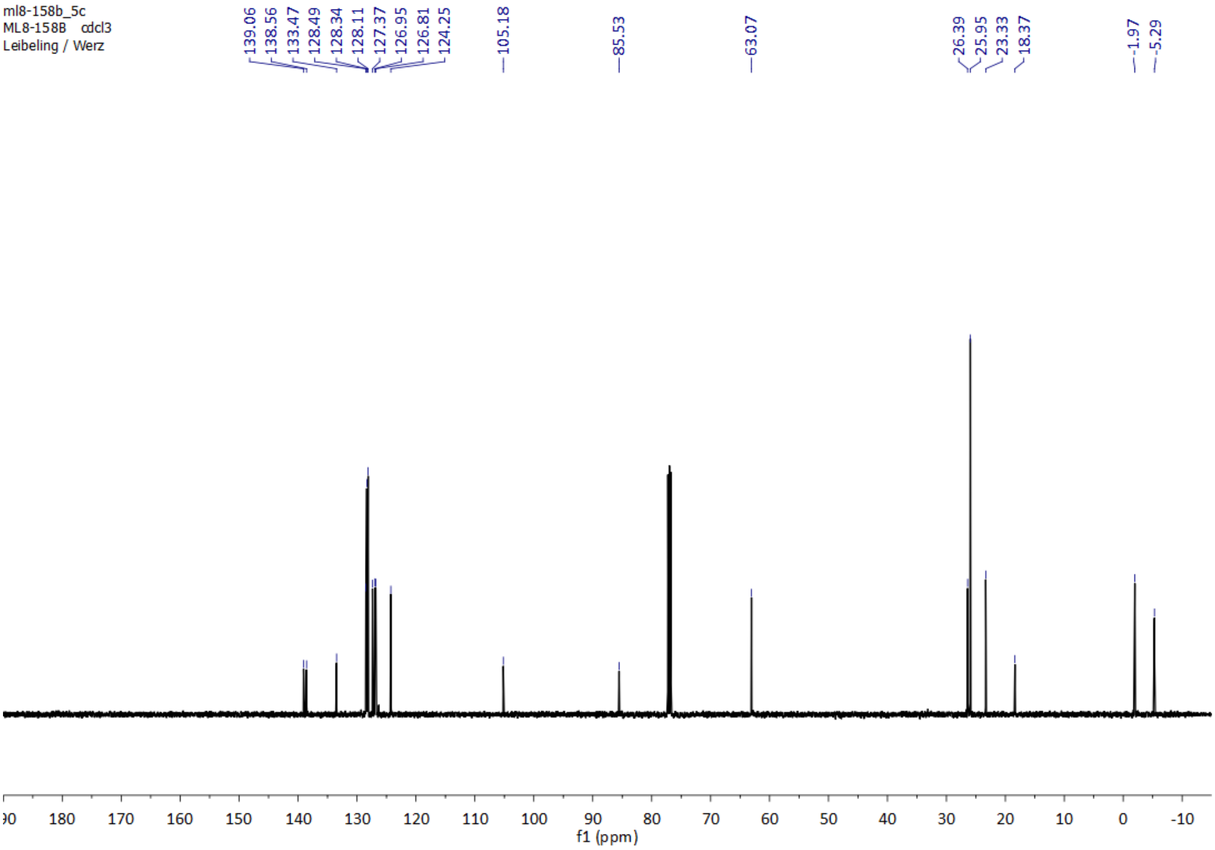

Compound 23c

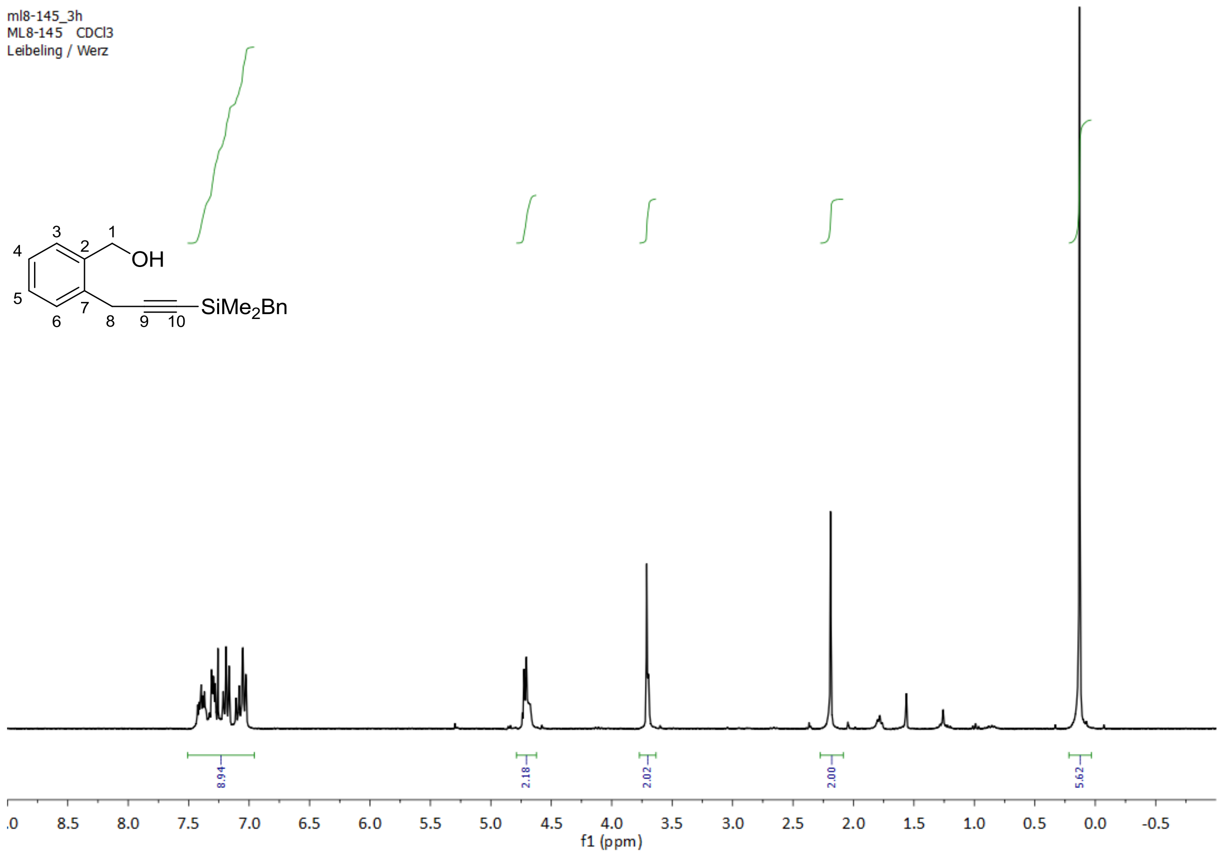

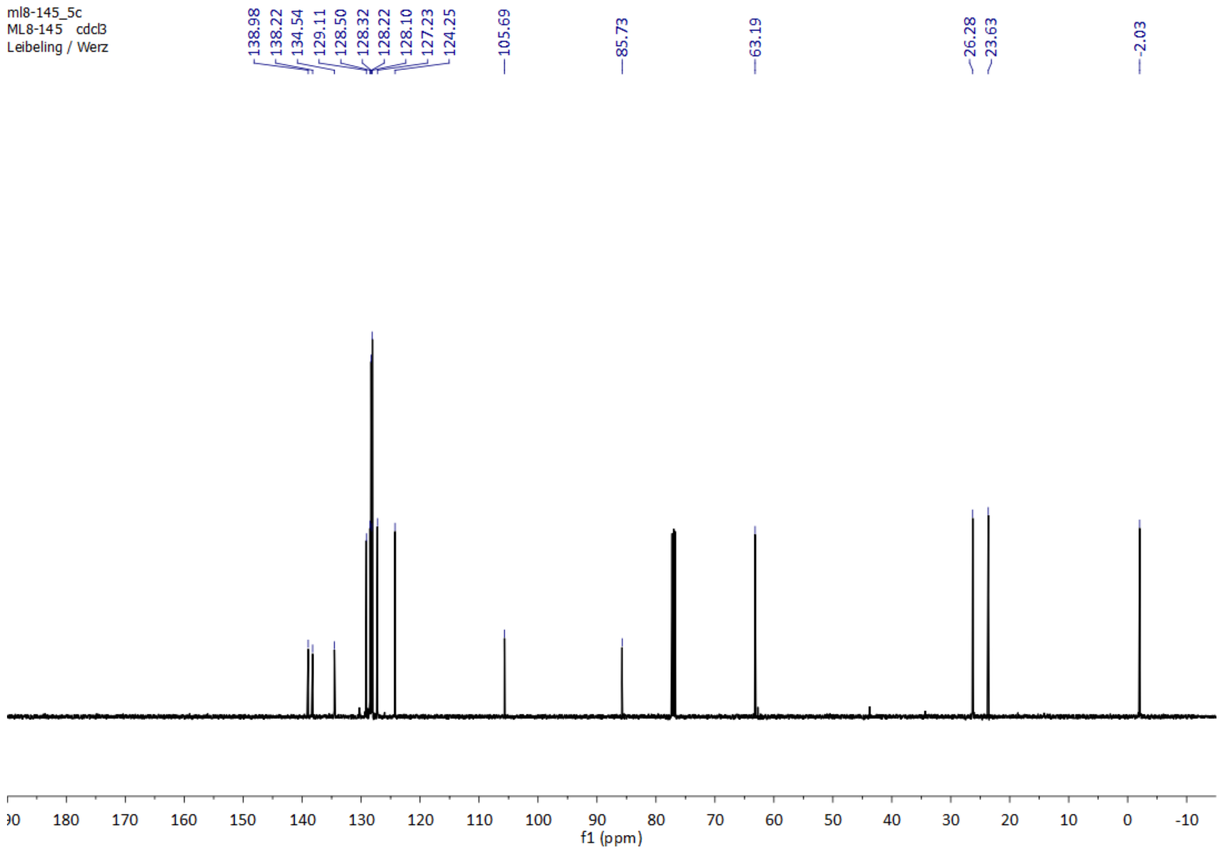

Compound 24c

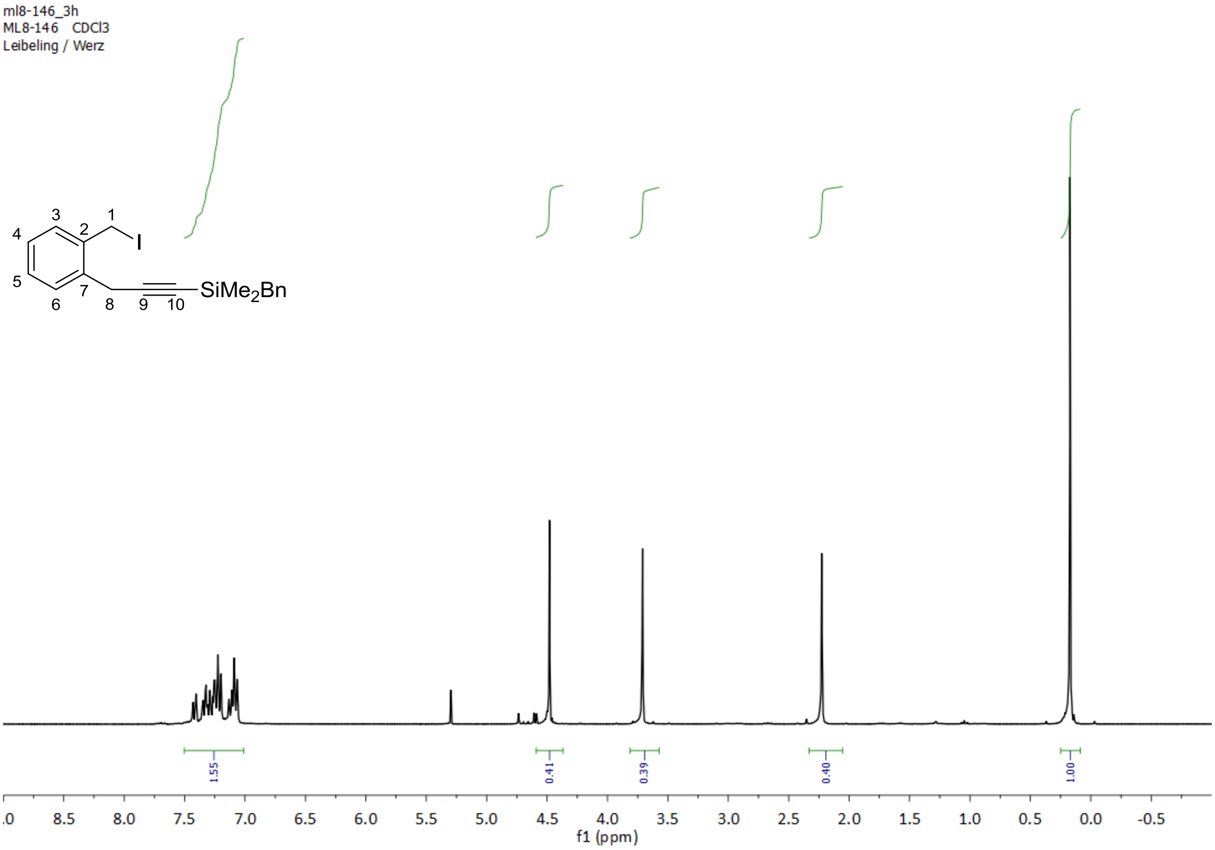

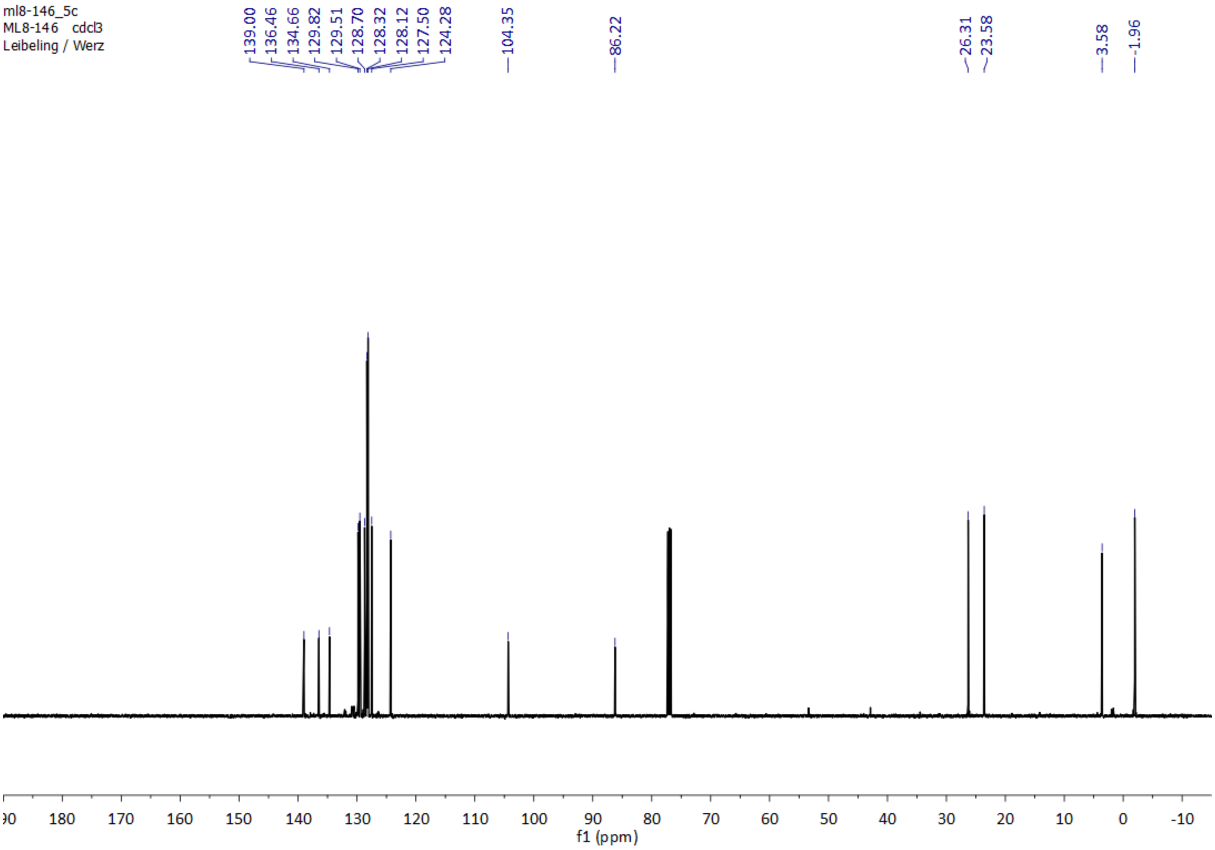

Compound 16c

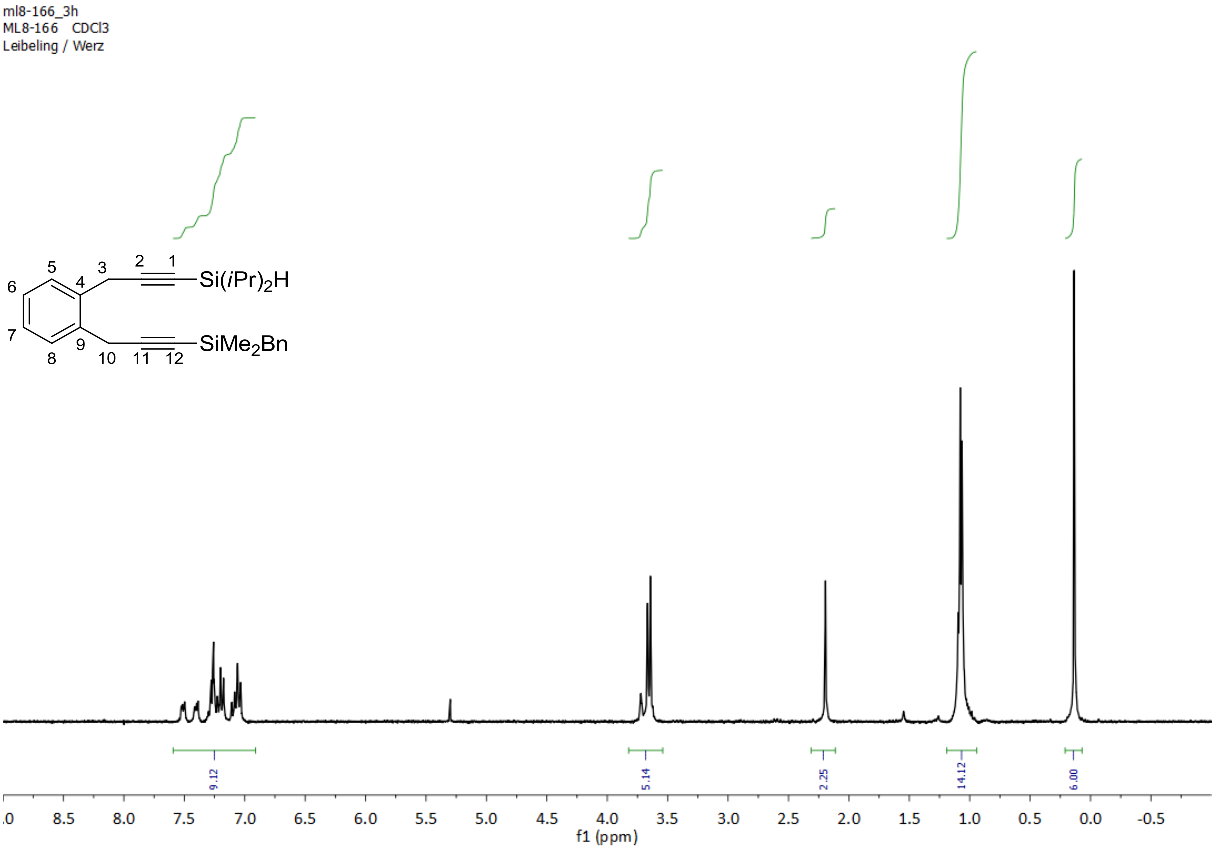

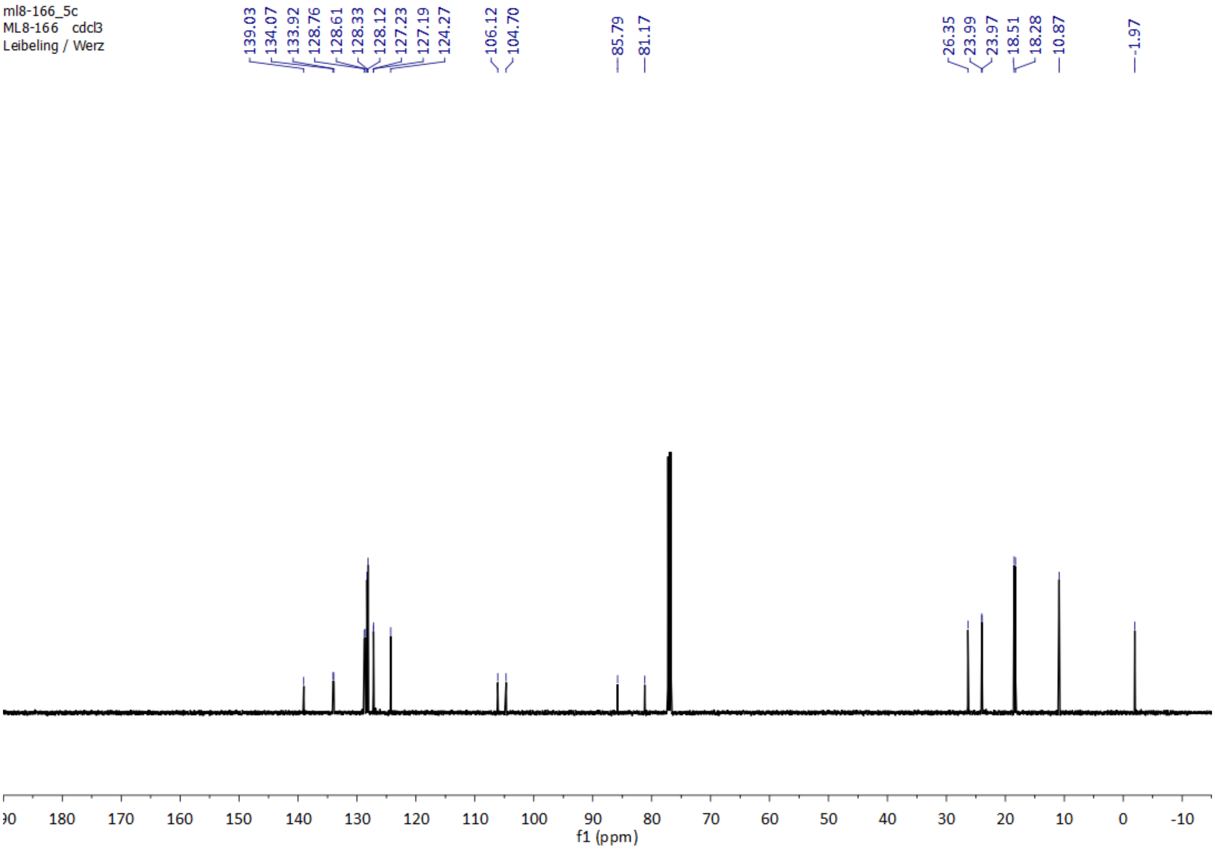

Compound 14c

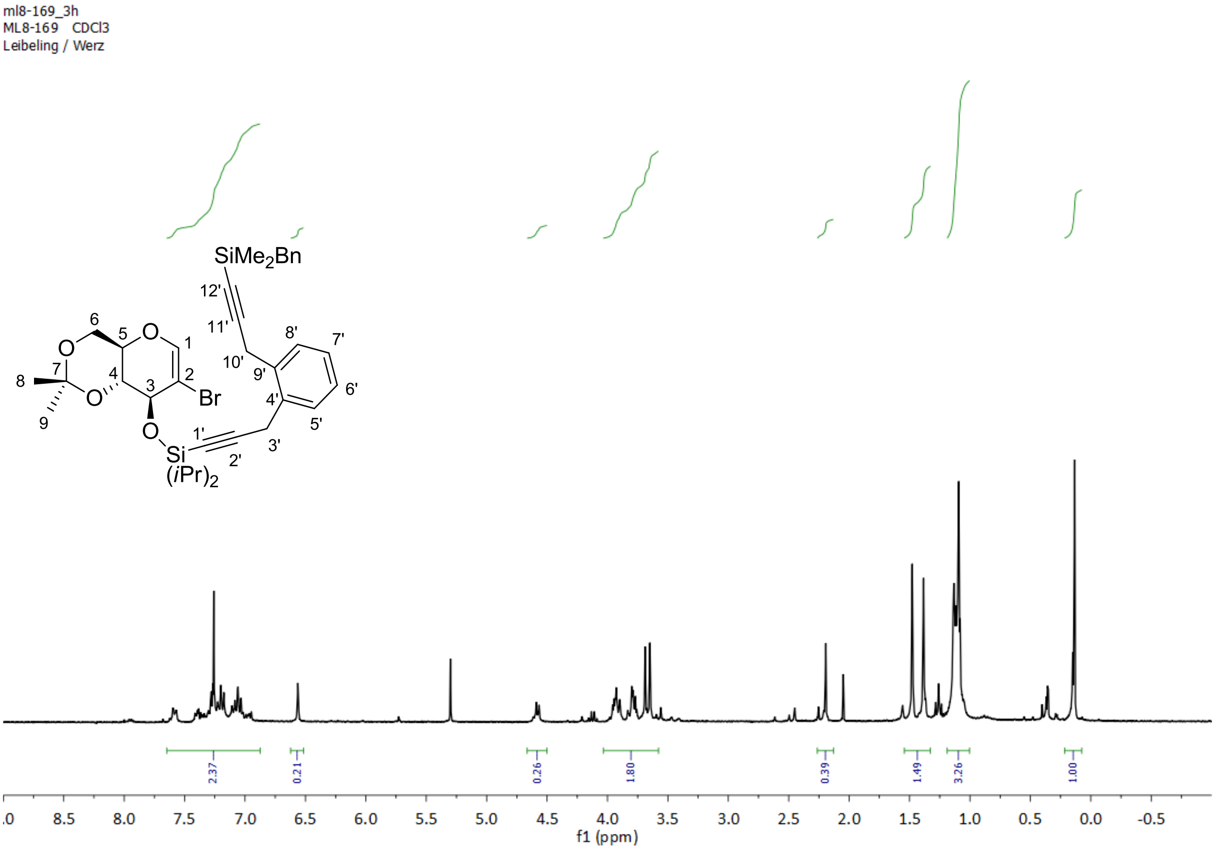

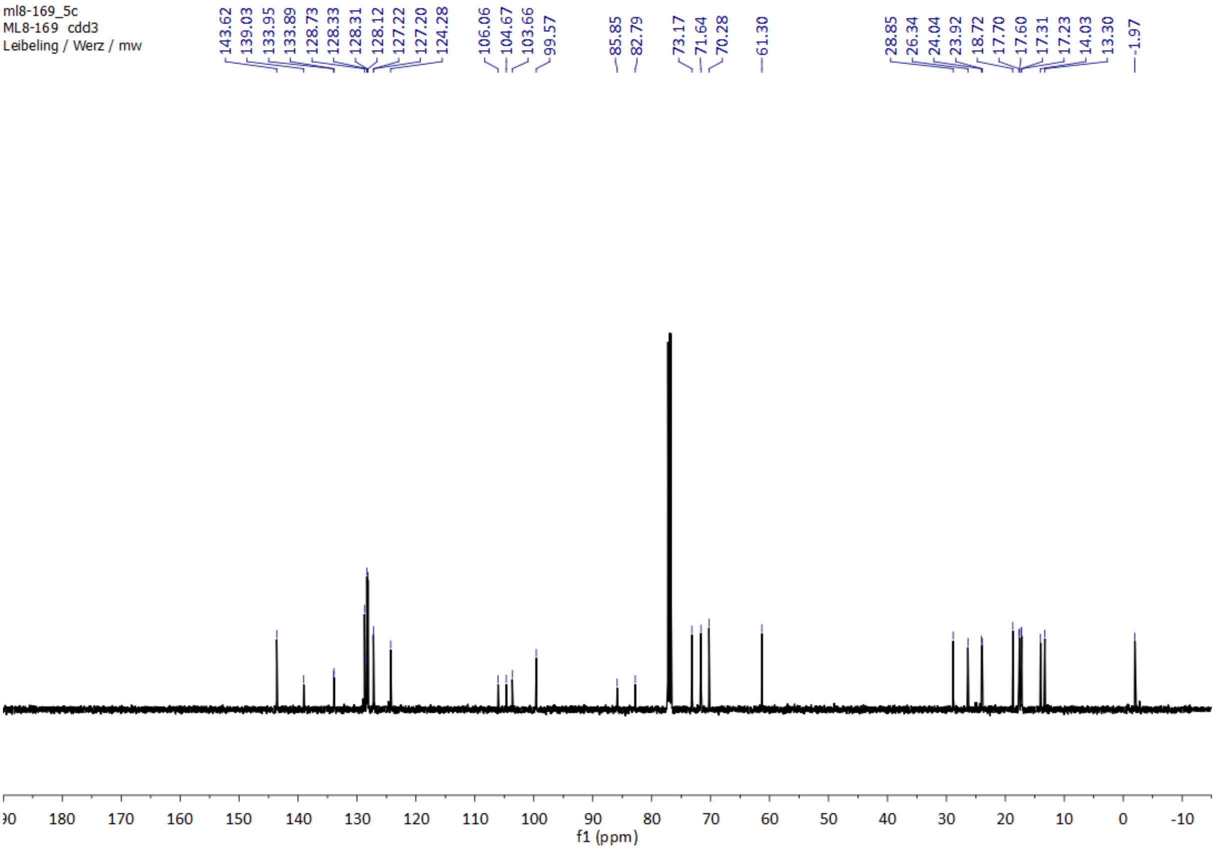

Compound 13c

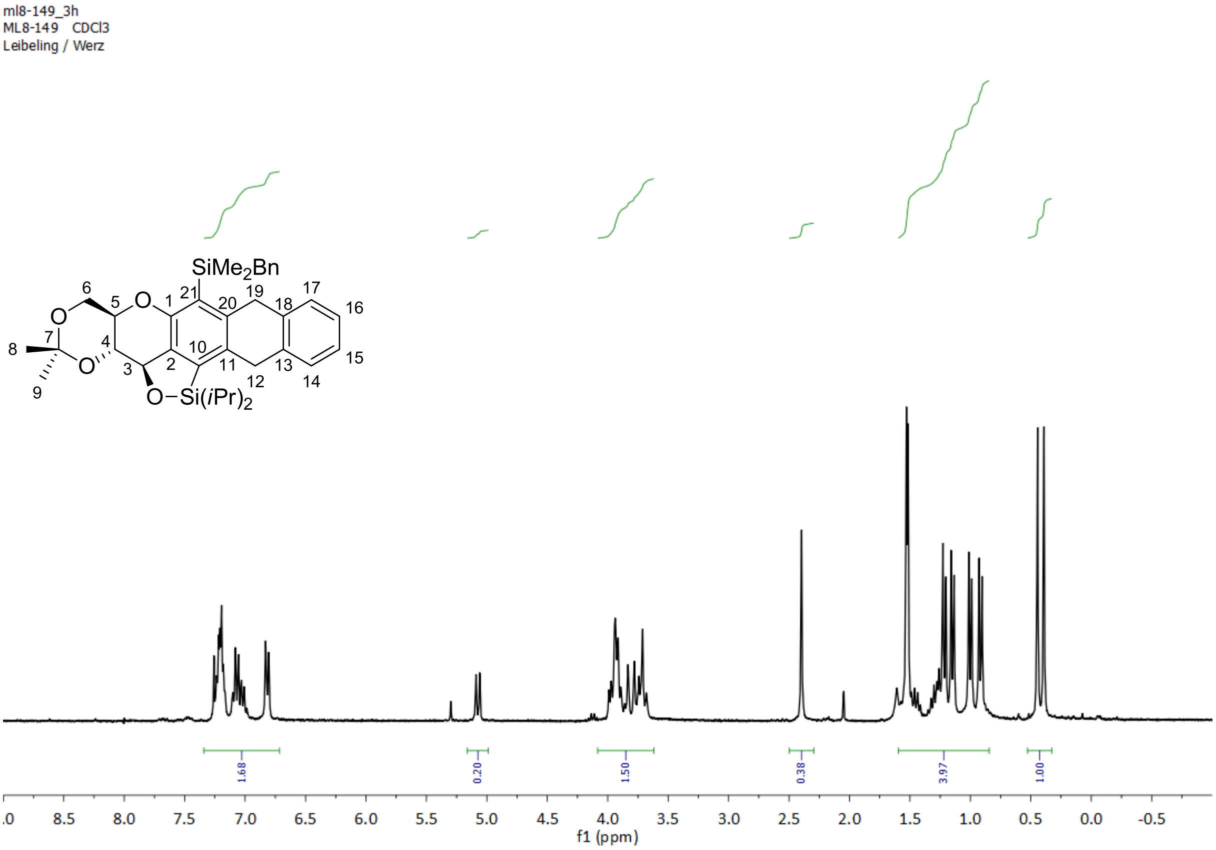

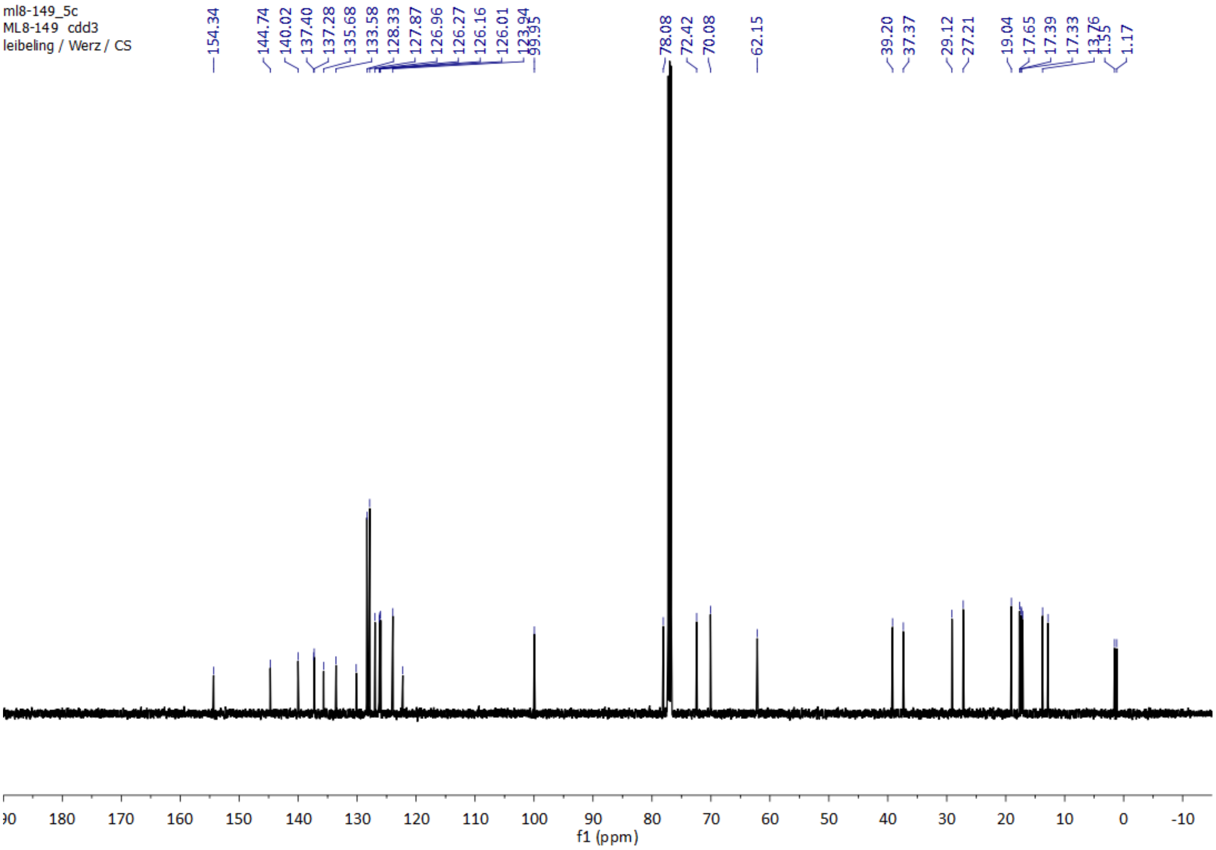

Compound 22d

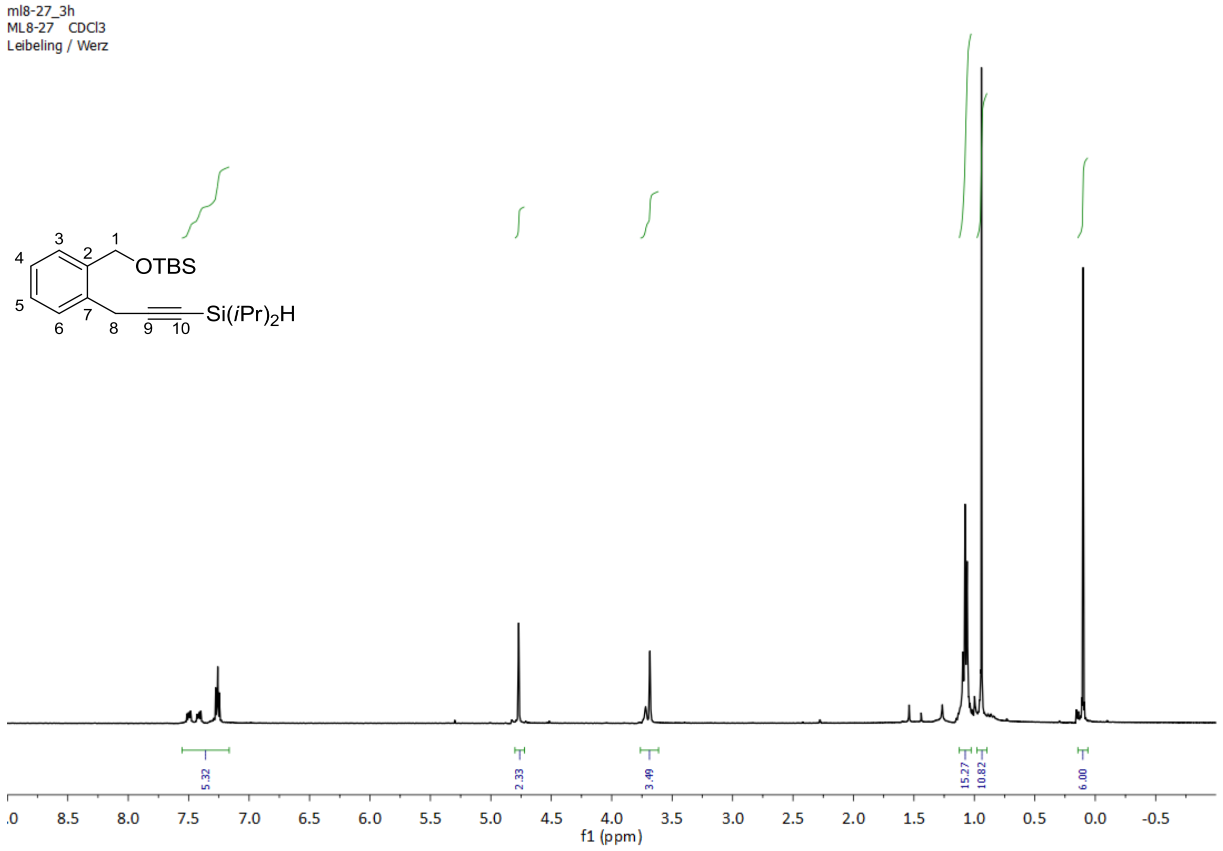

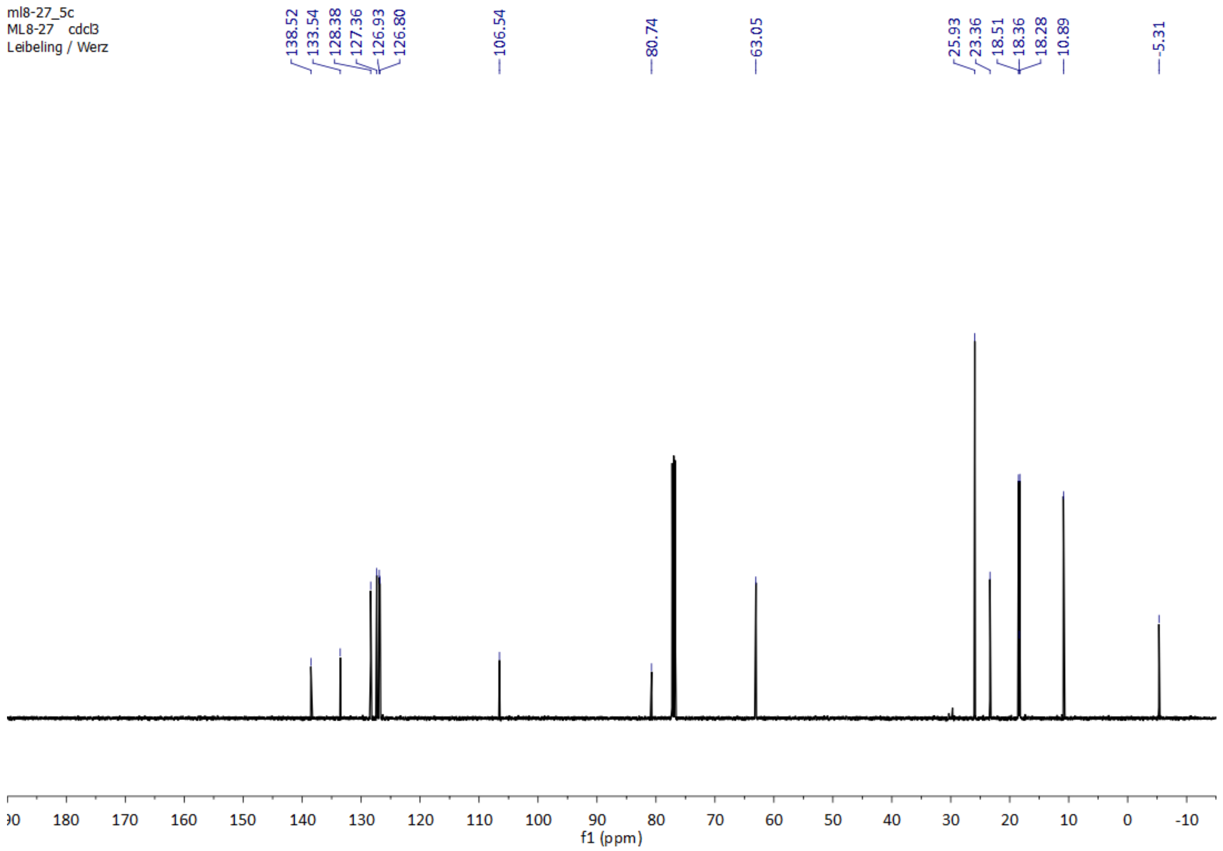

Compound 23d

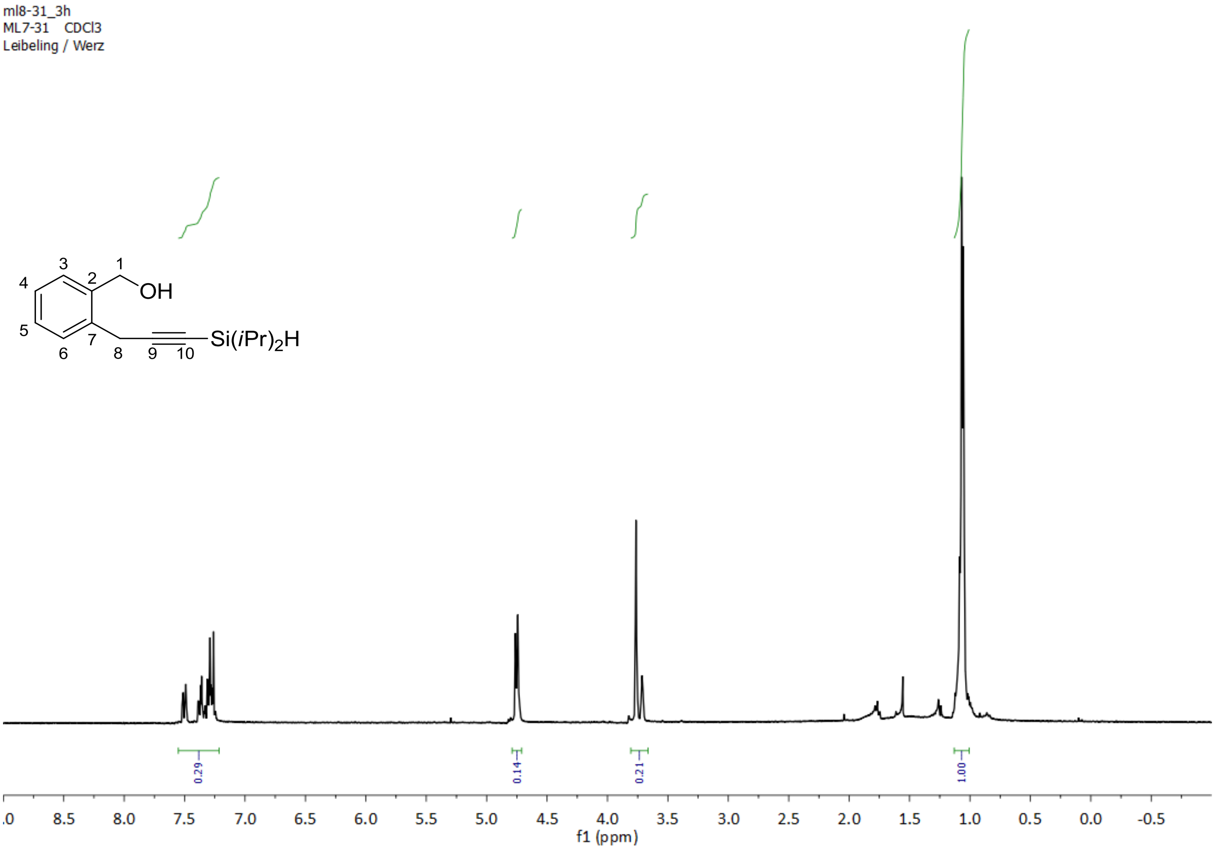

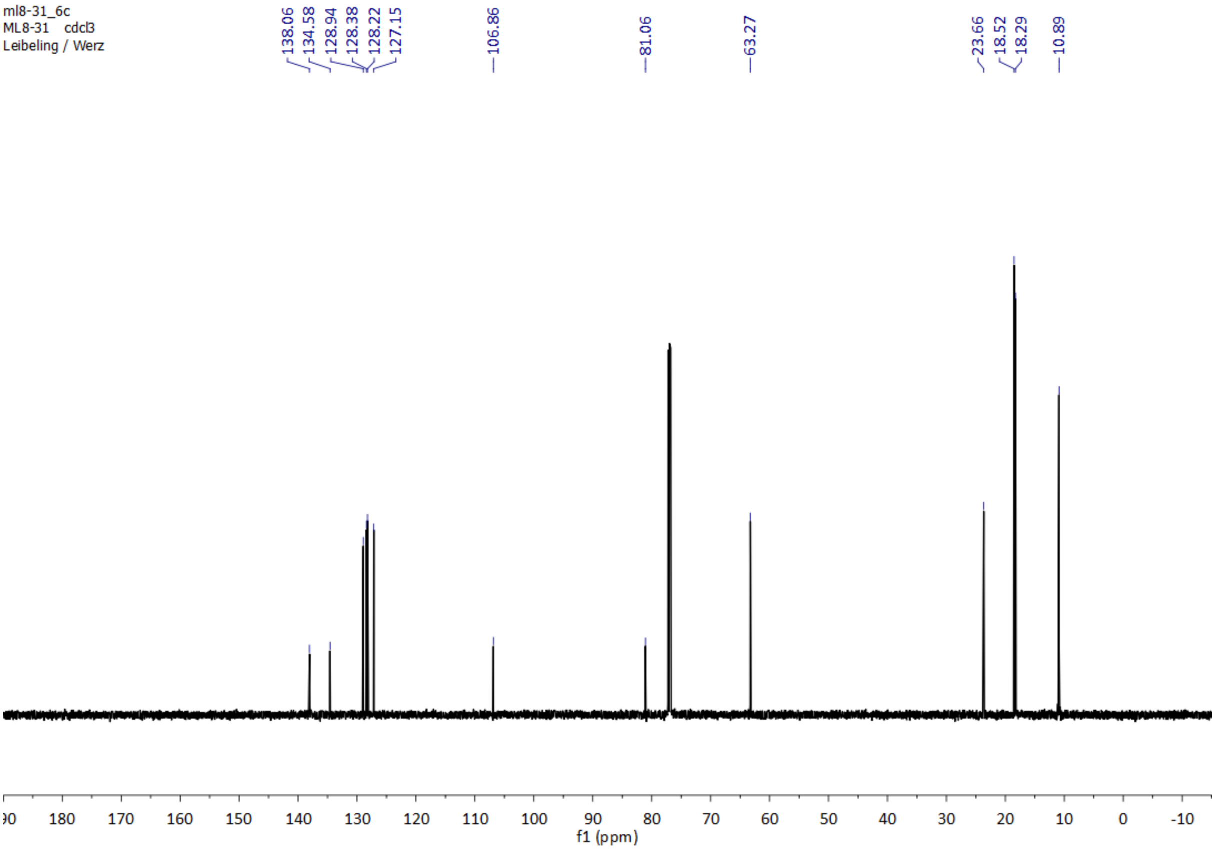

Compound 24d

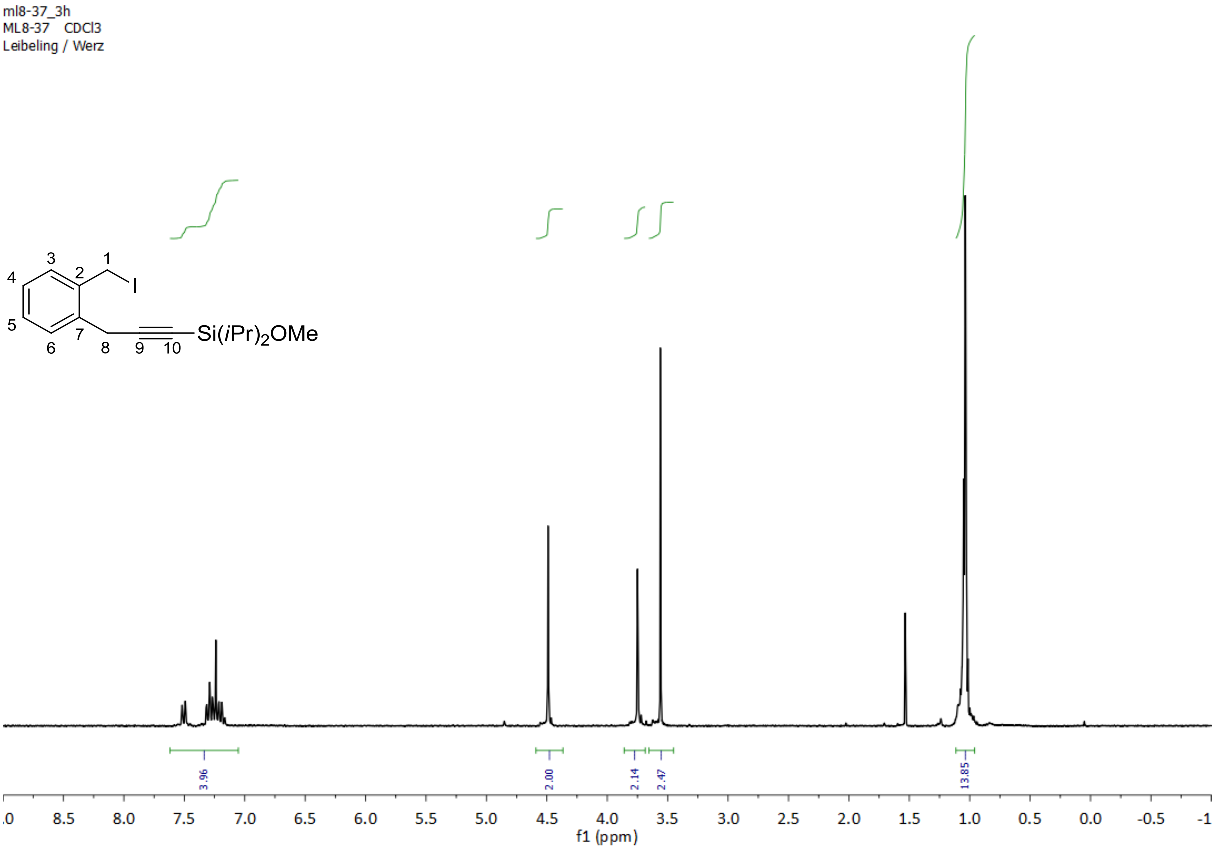

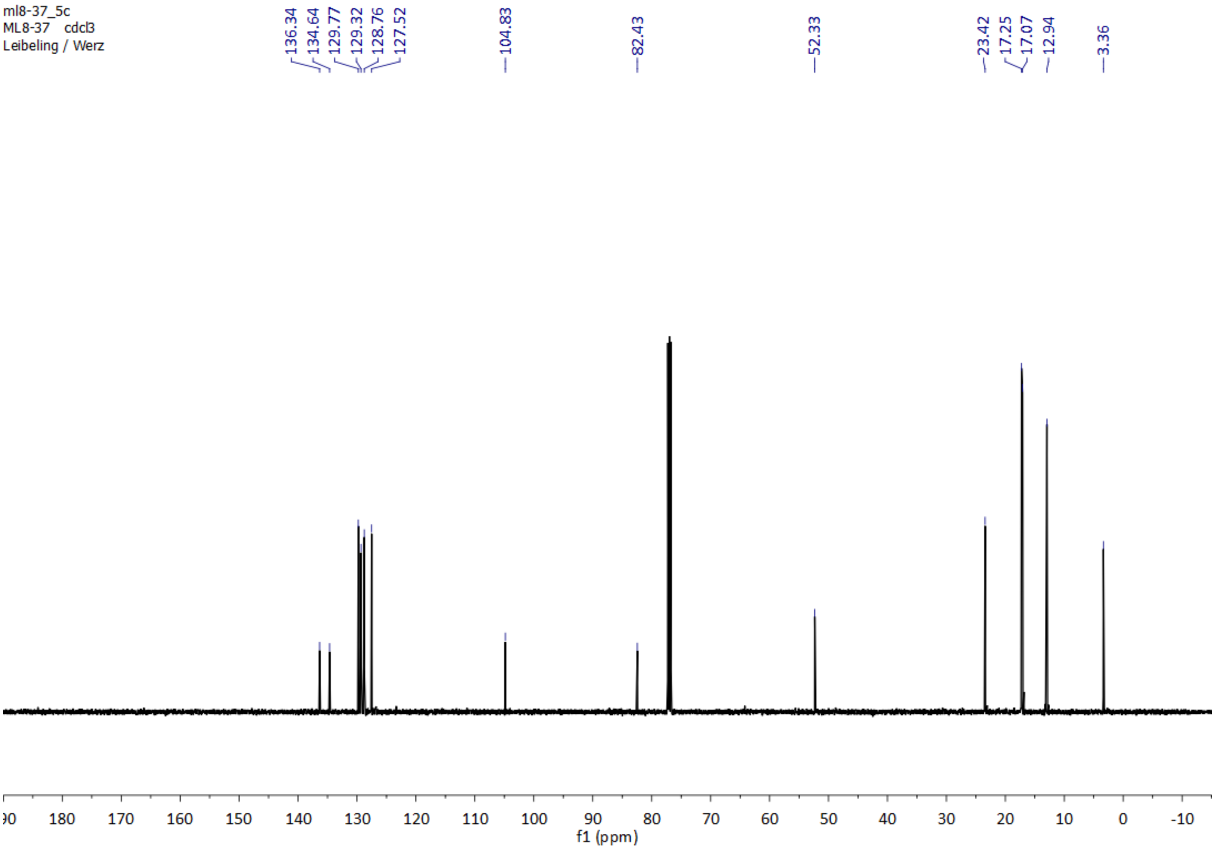

Compound 16d

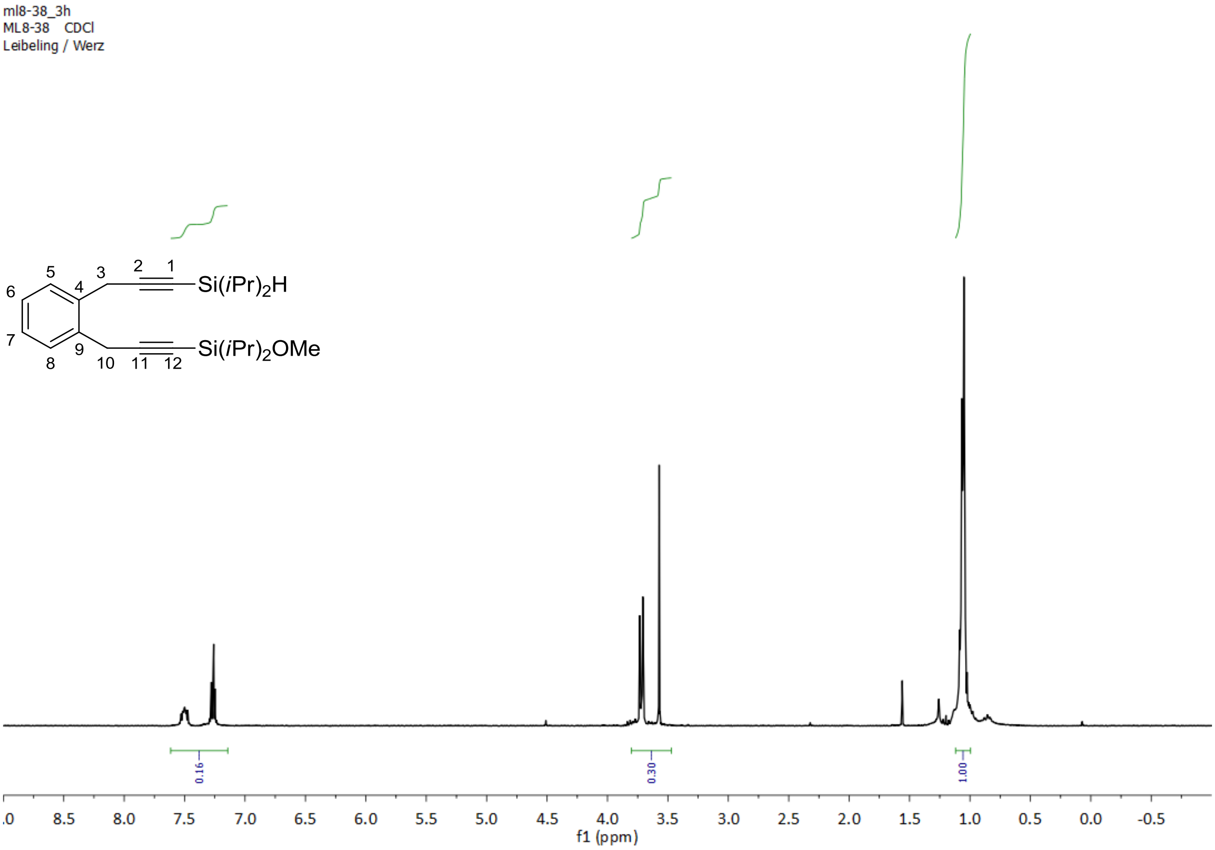

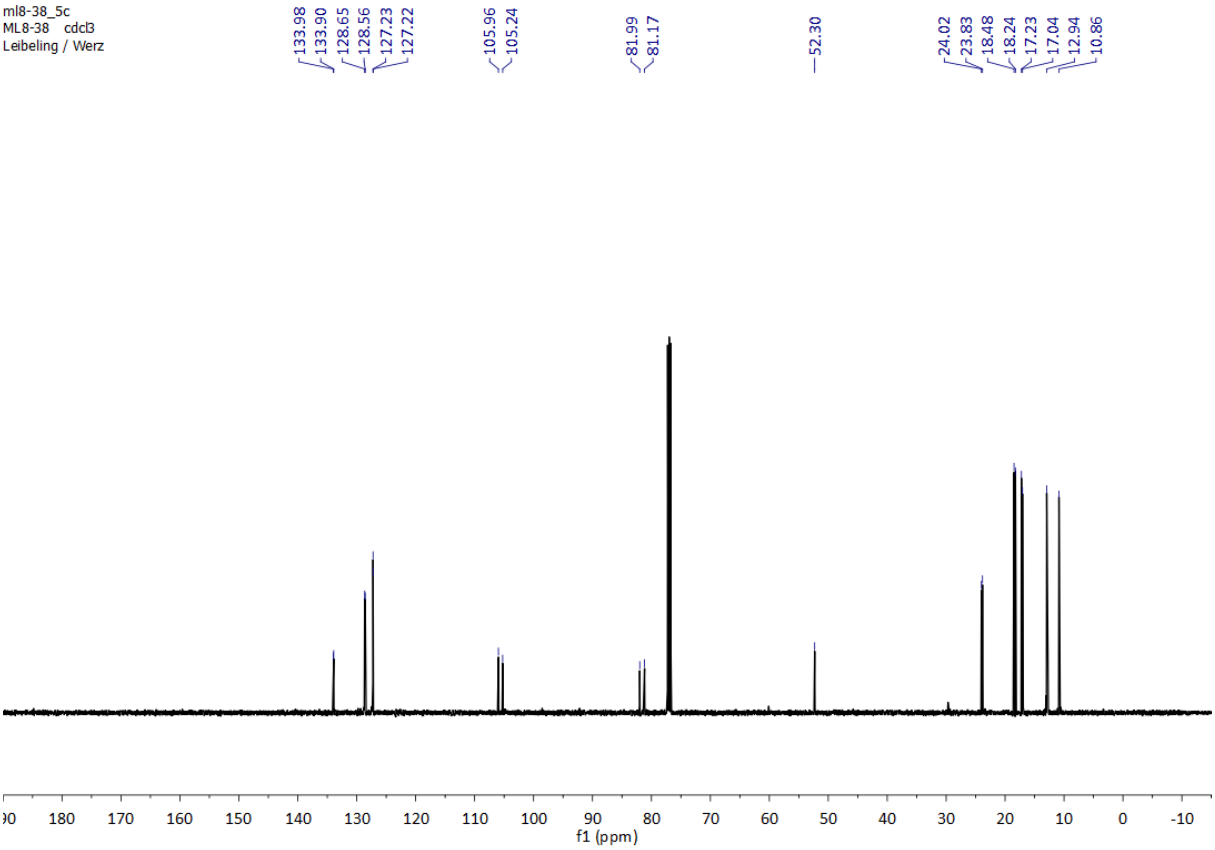

Compound 14d

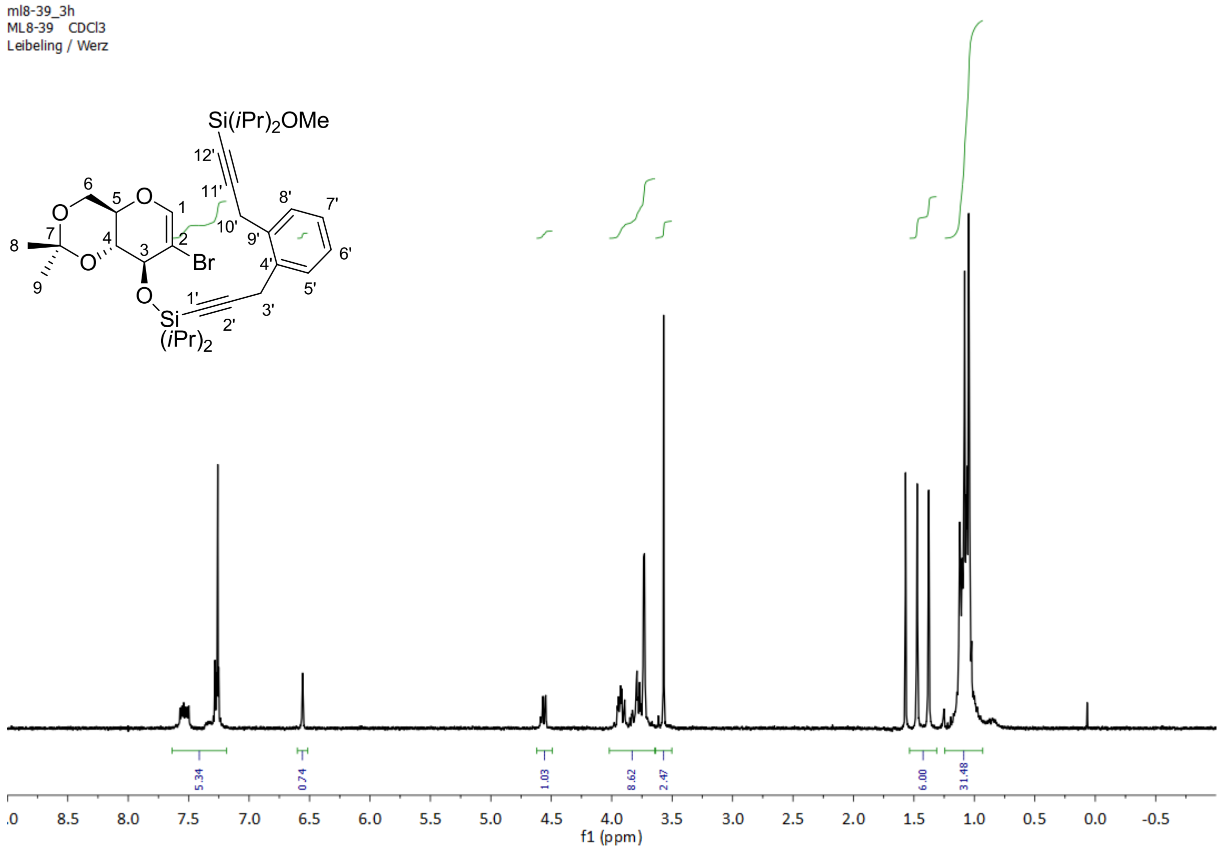

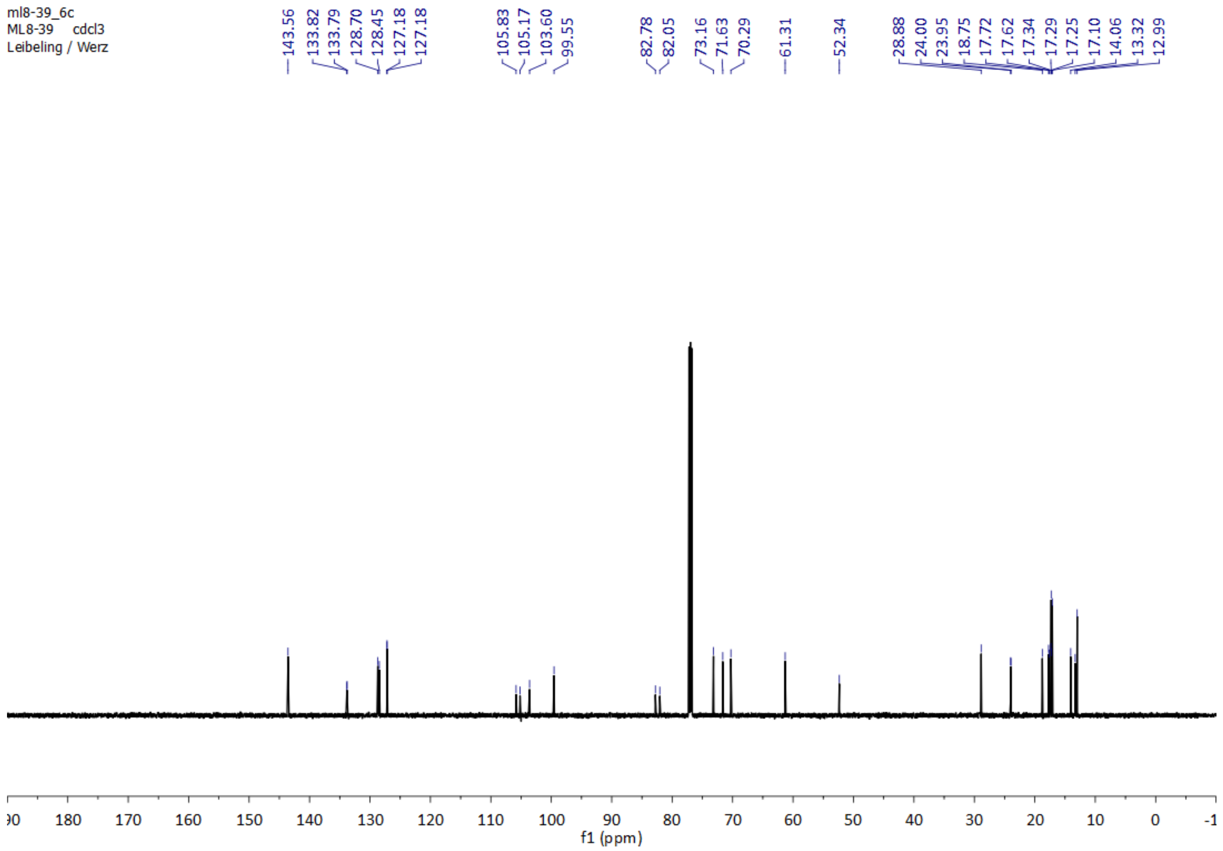

Compound 13d

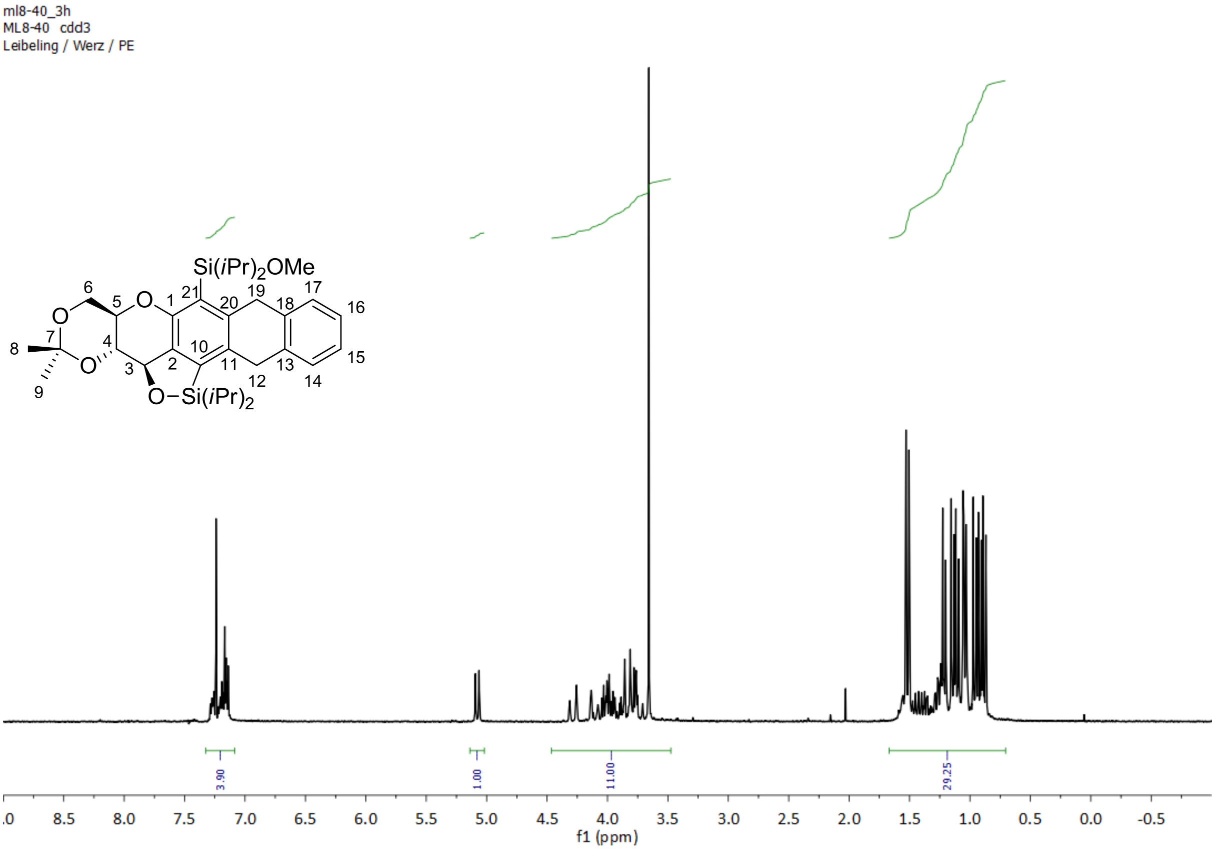

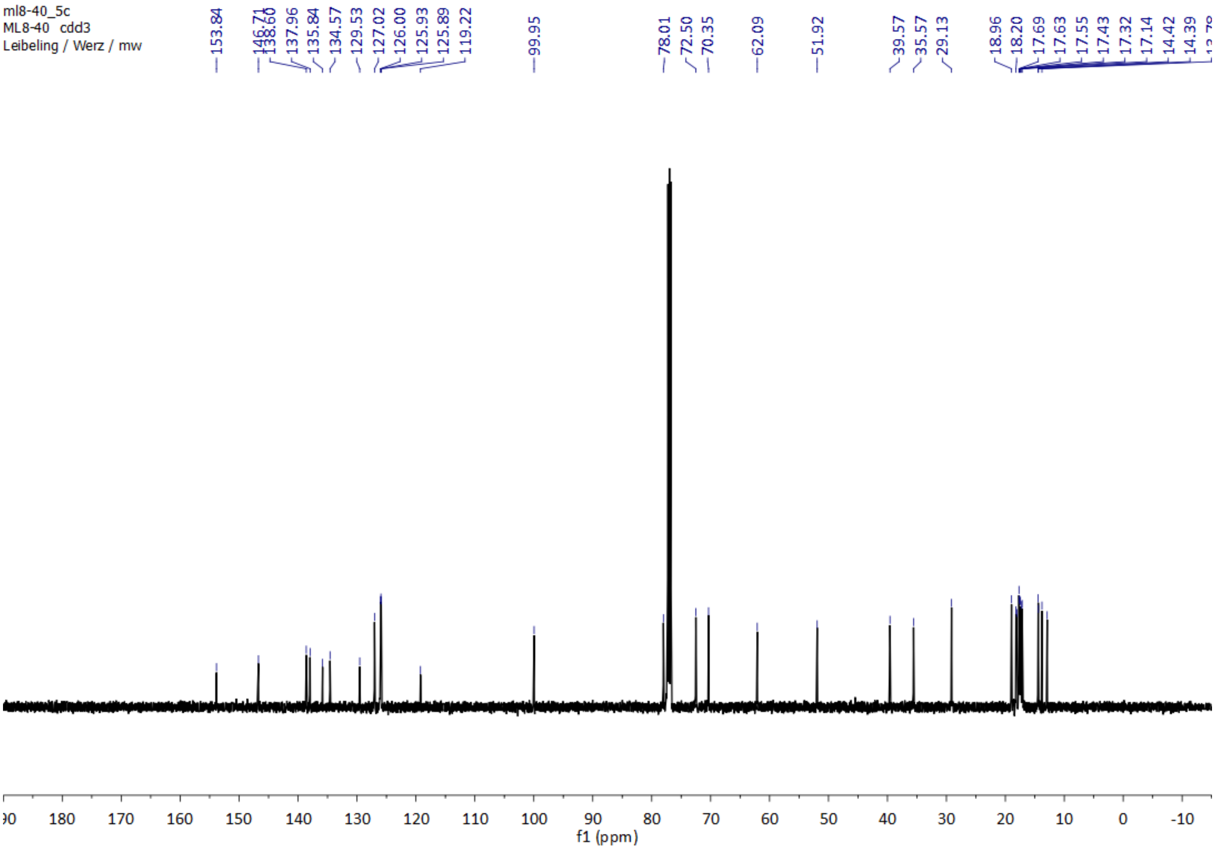

Compound 24e

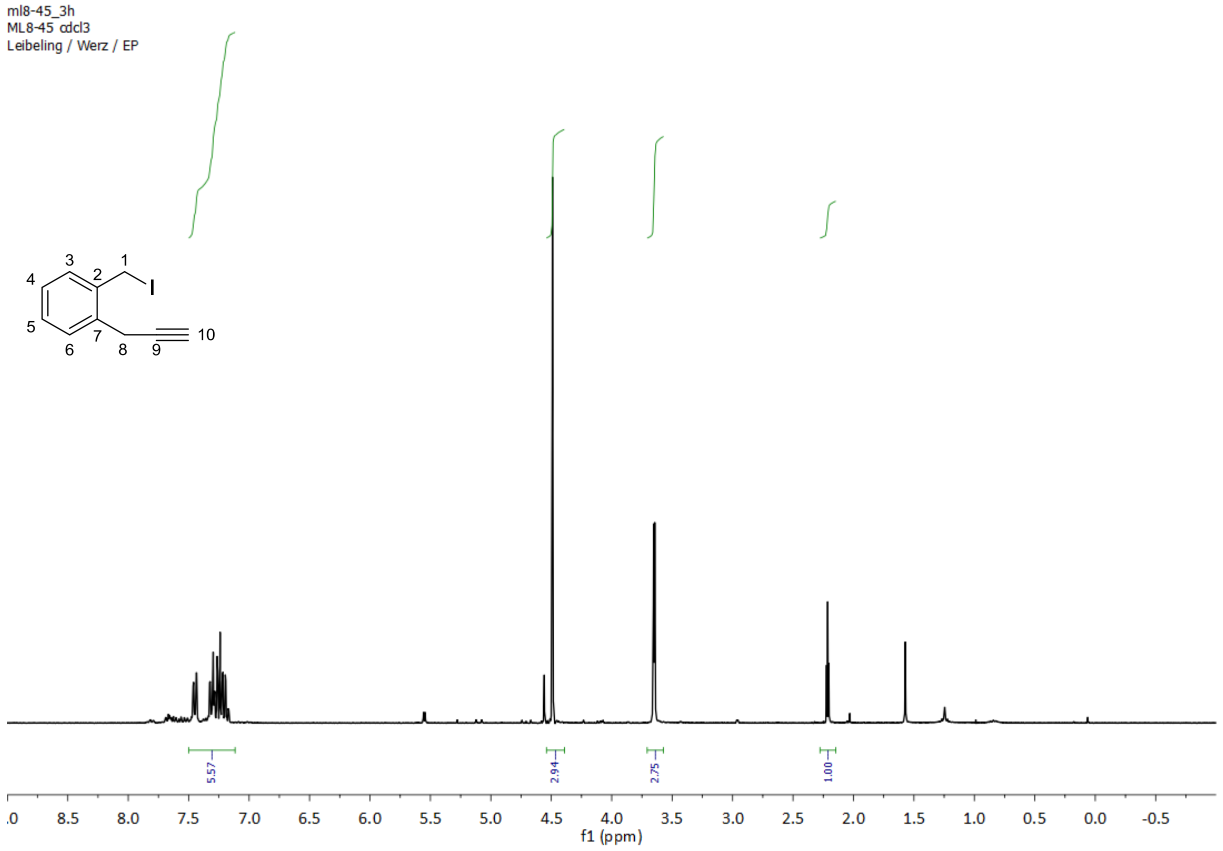

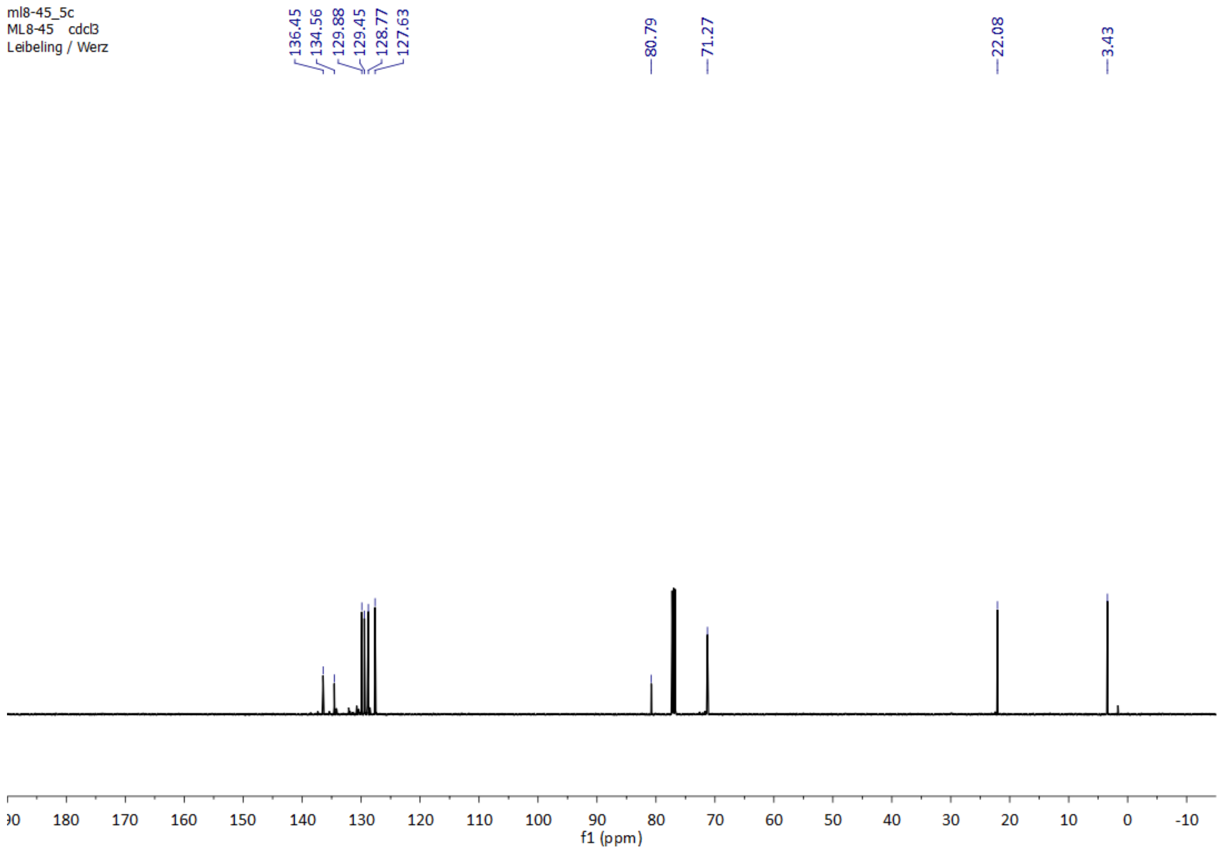

Compound 16e

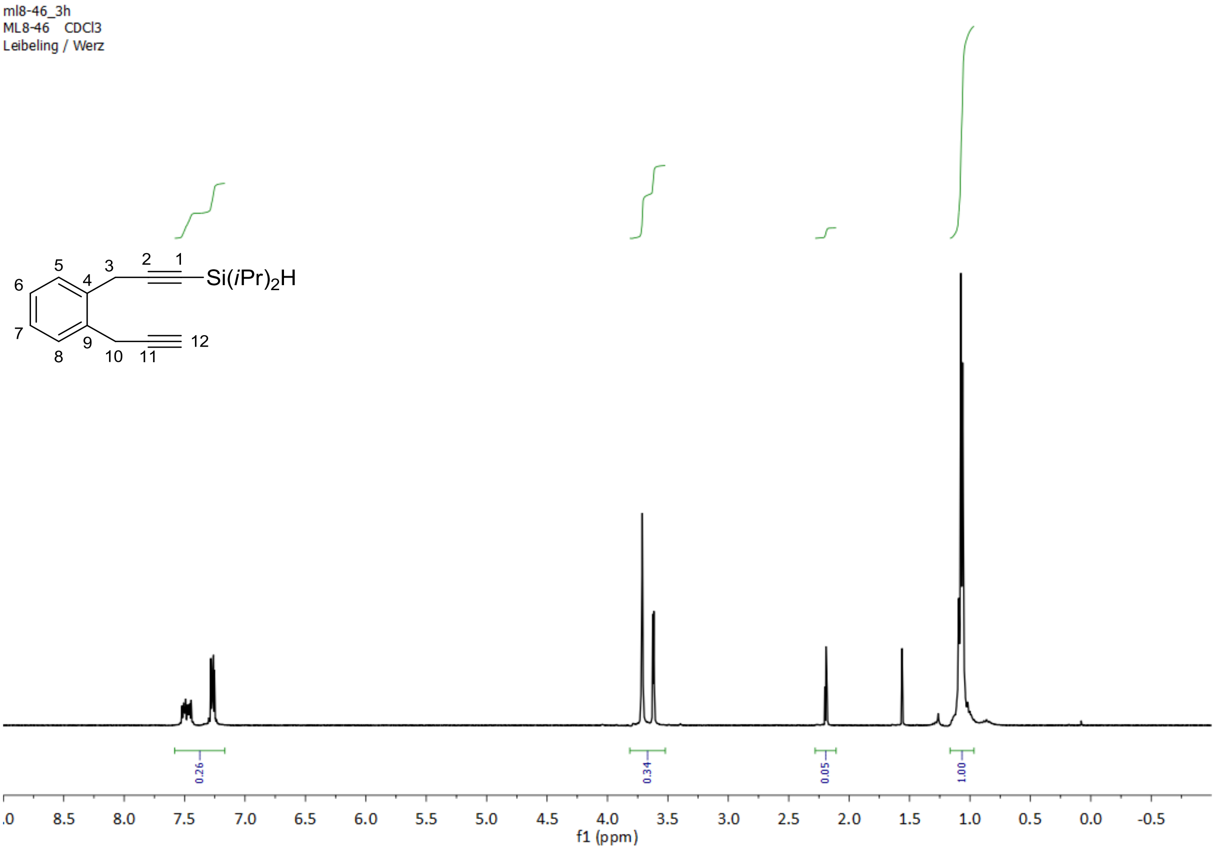

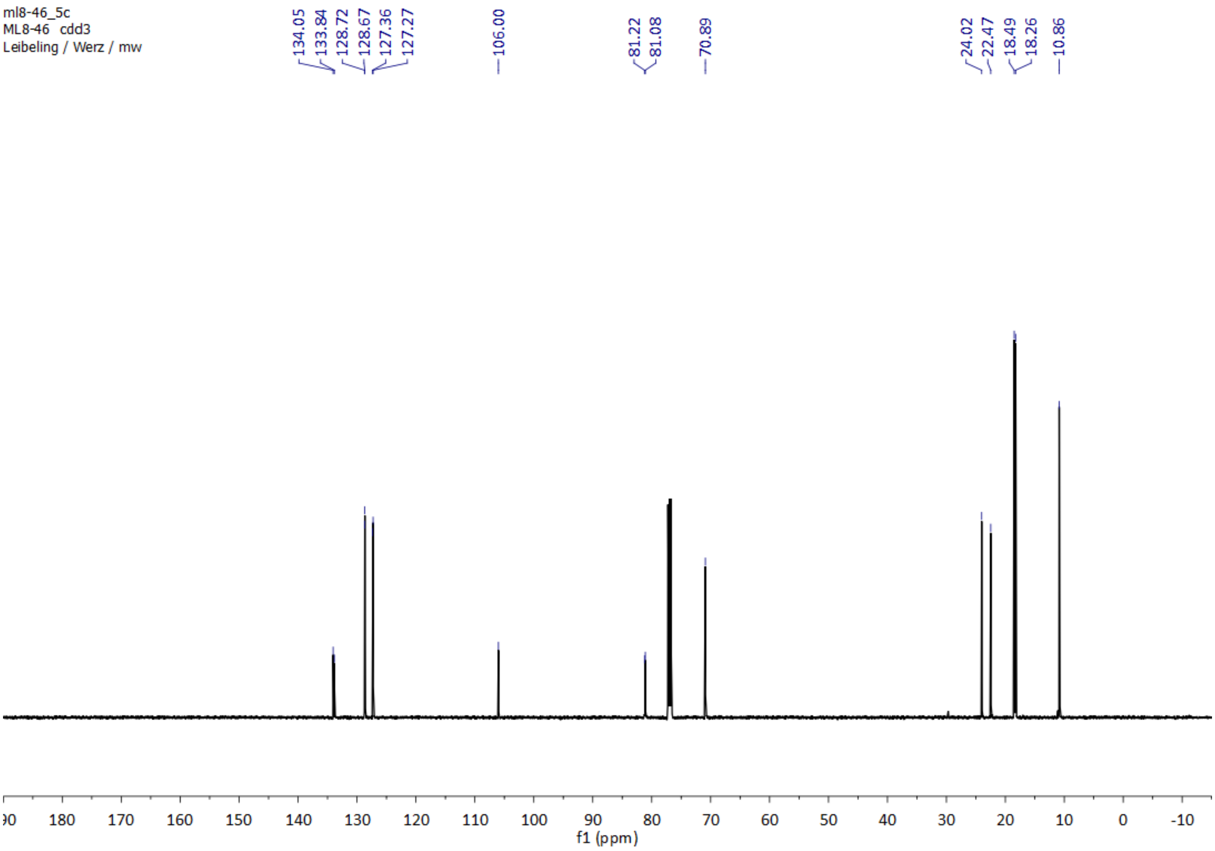

Compound 14e

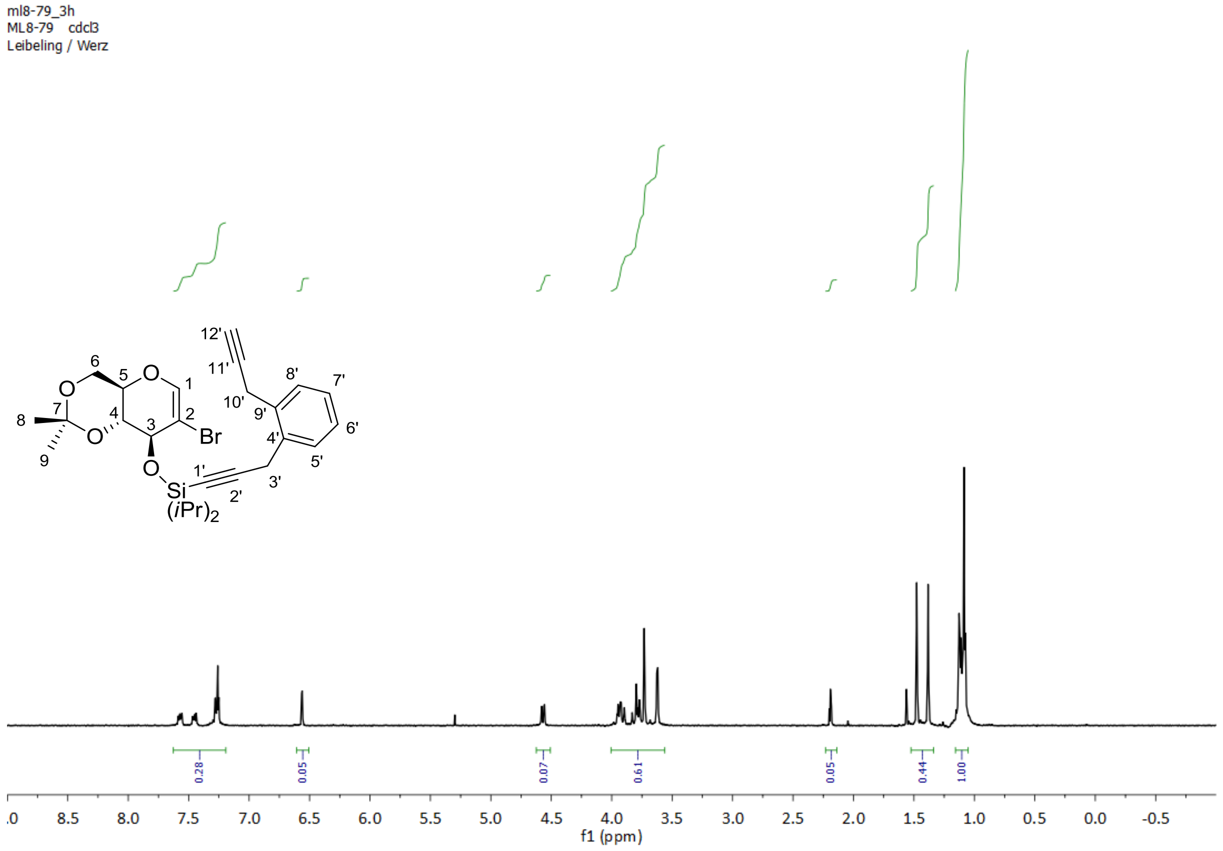

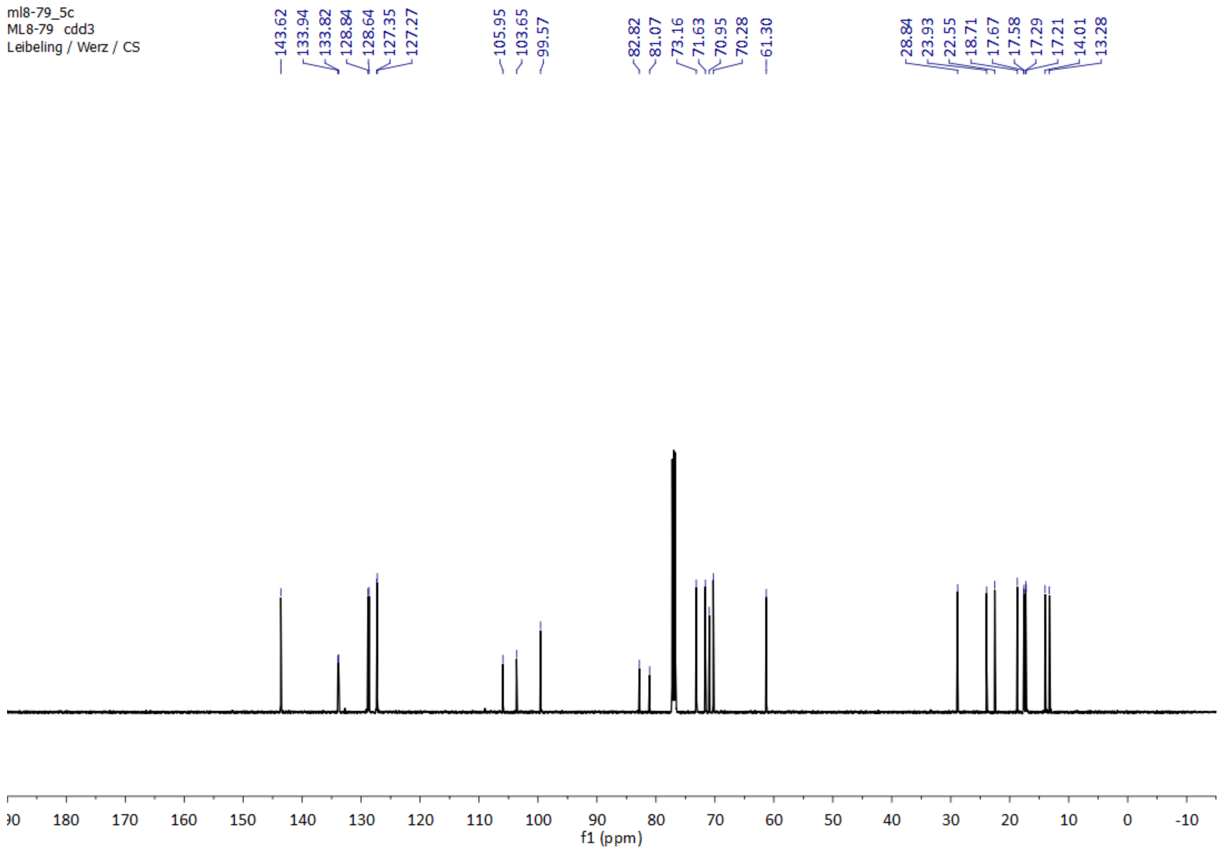

Compound 13e

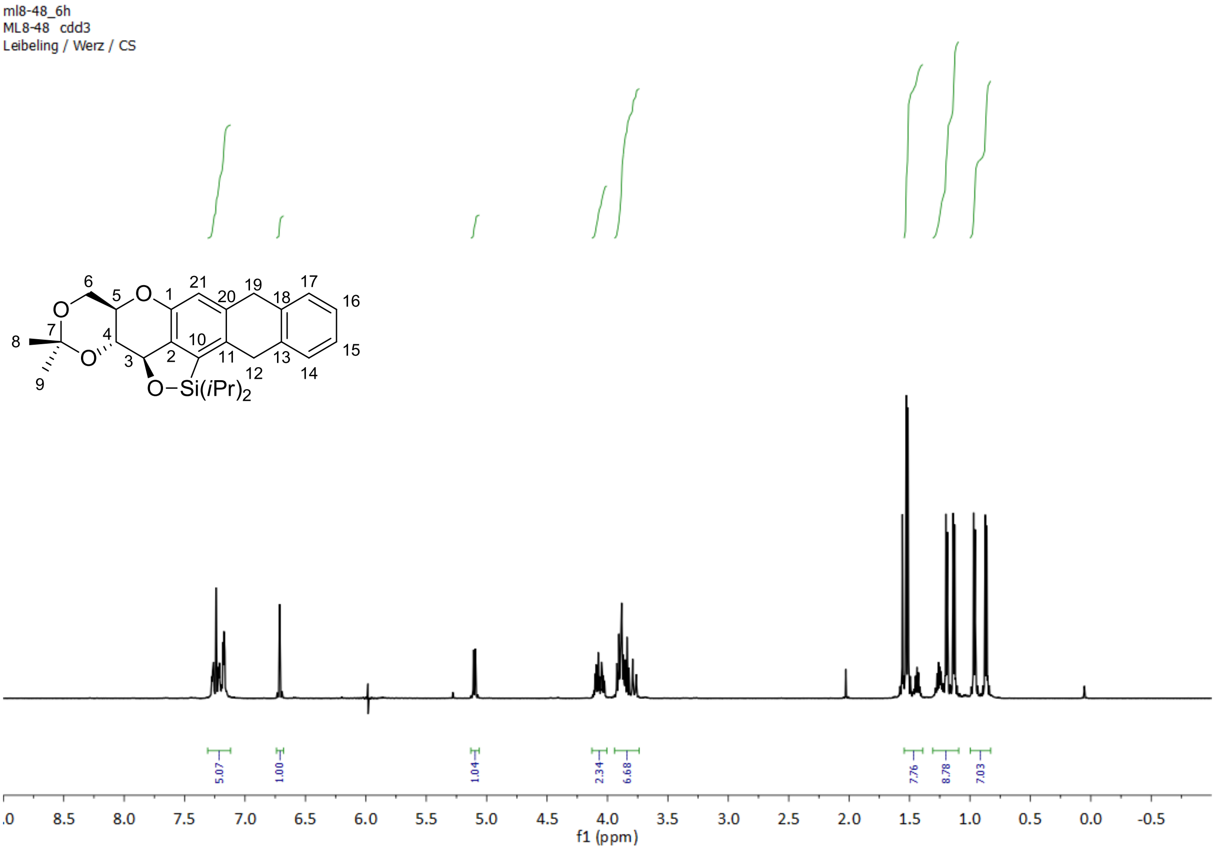

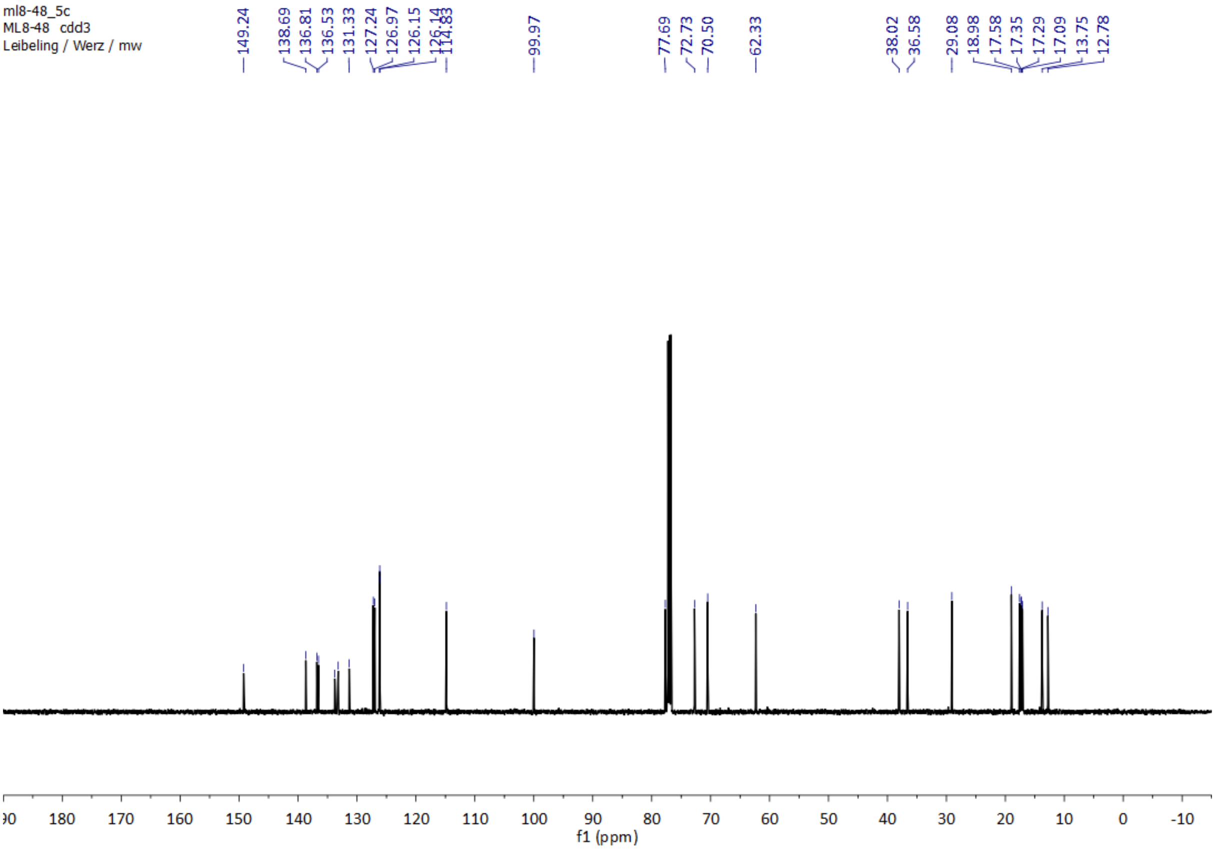

Compound 14f

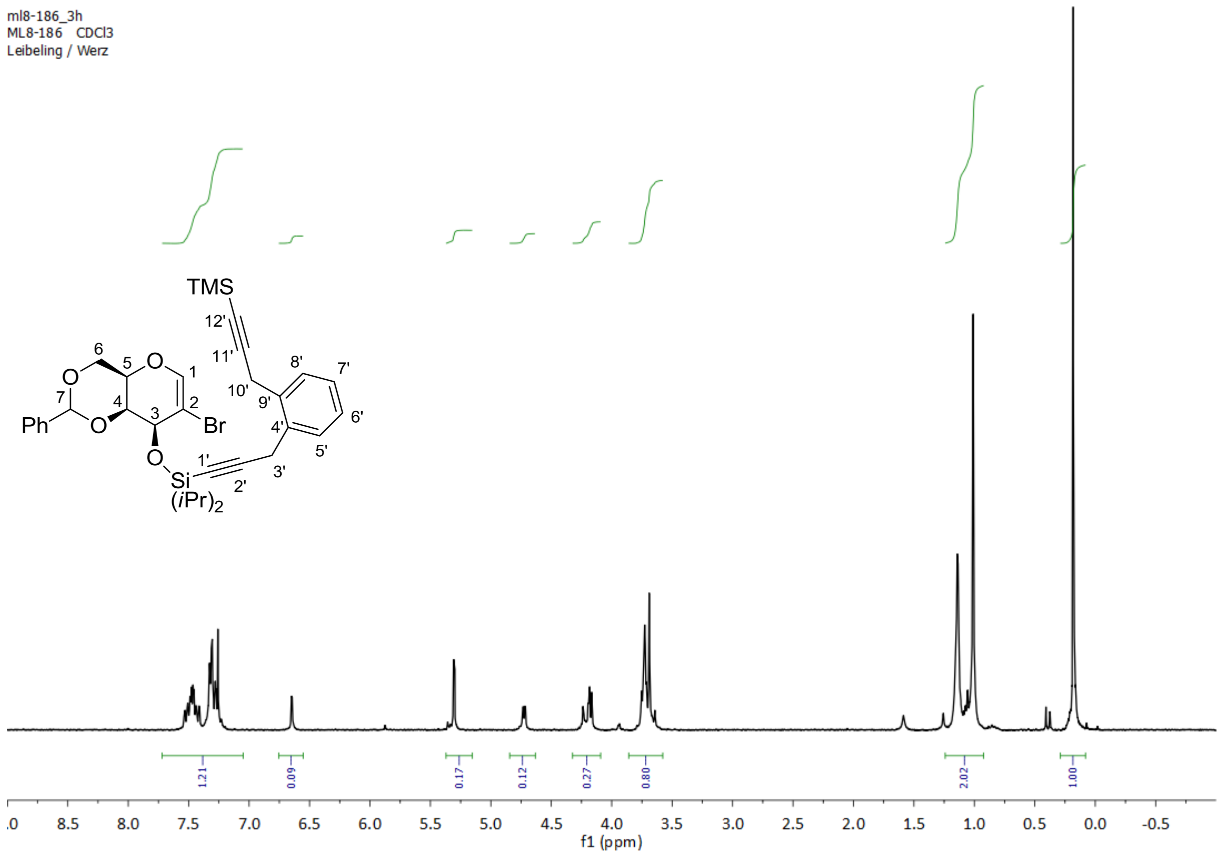

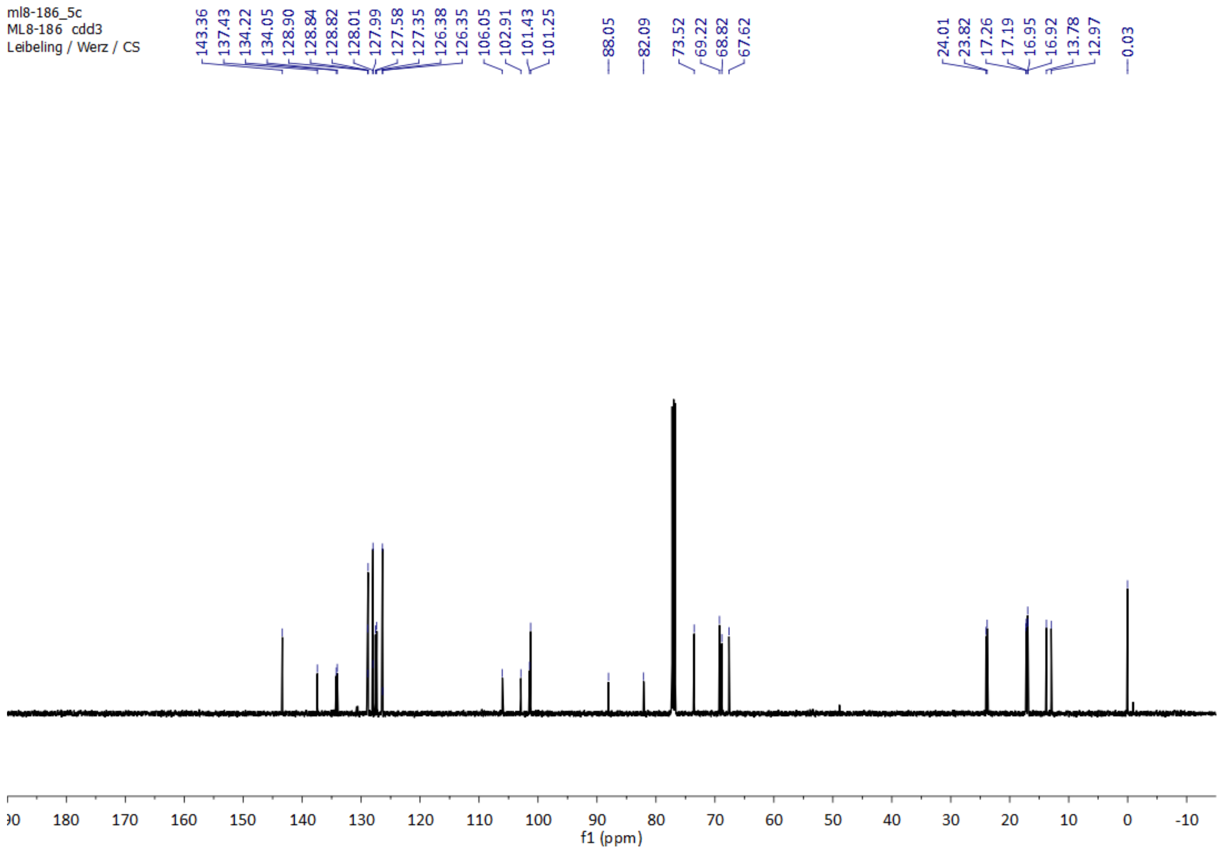

Compound 13f

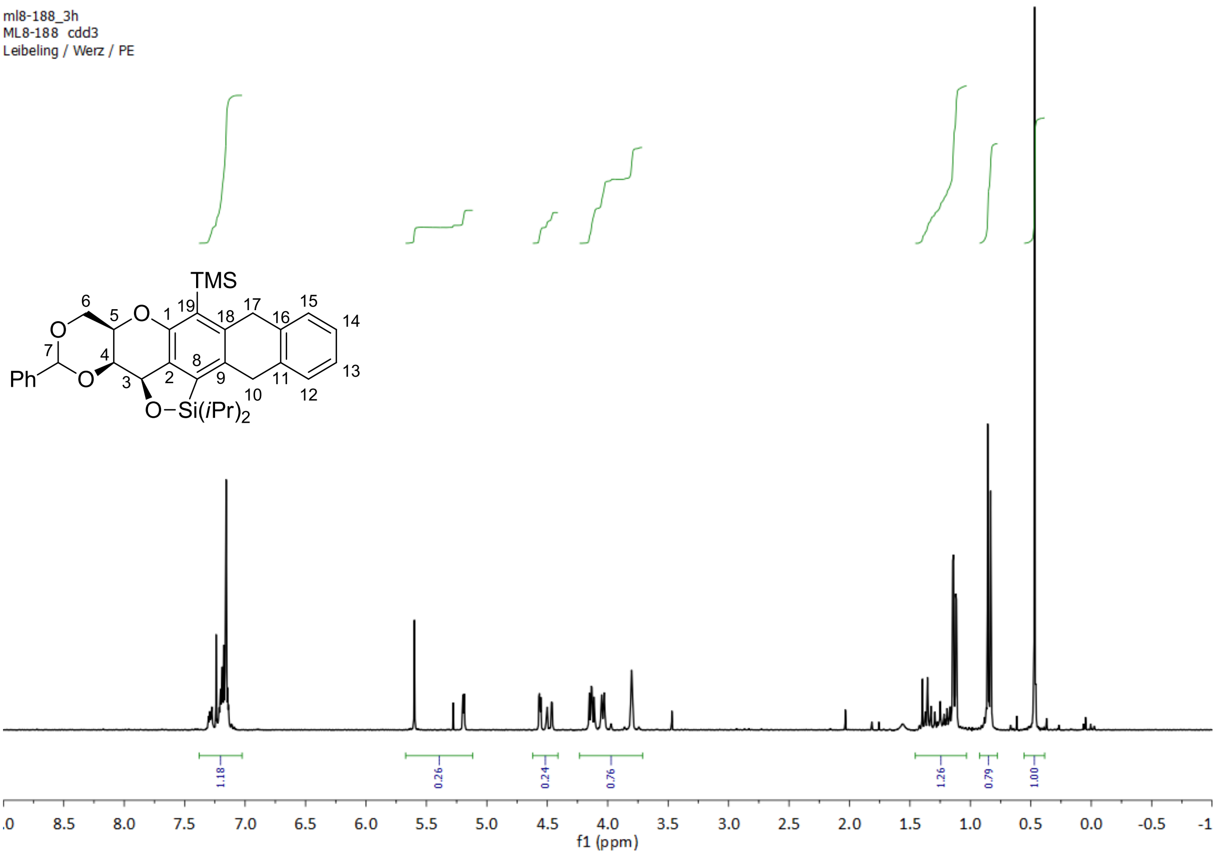

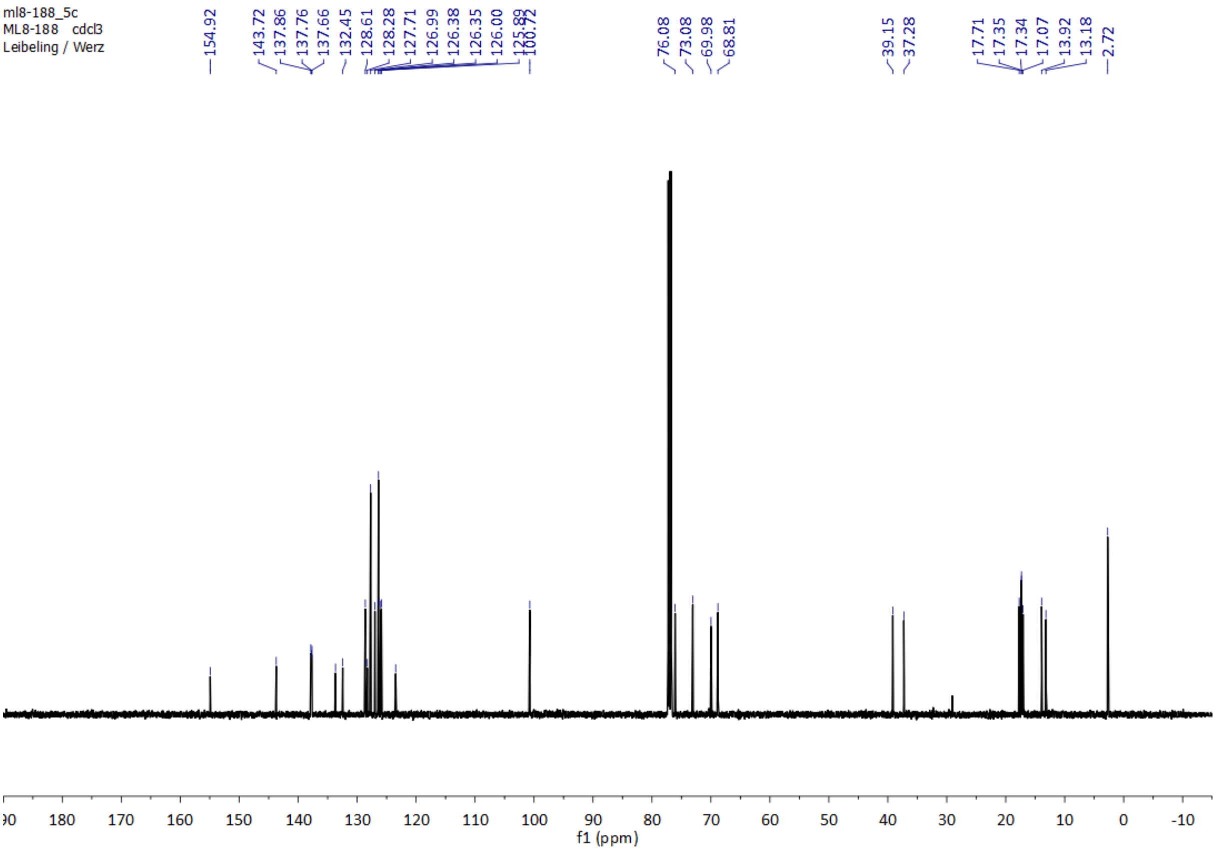

Compound 26b

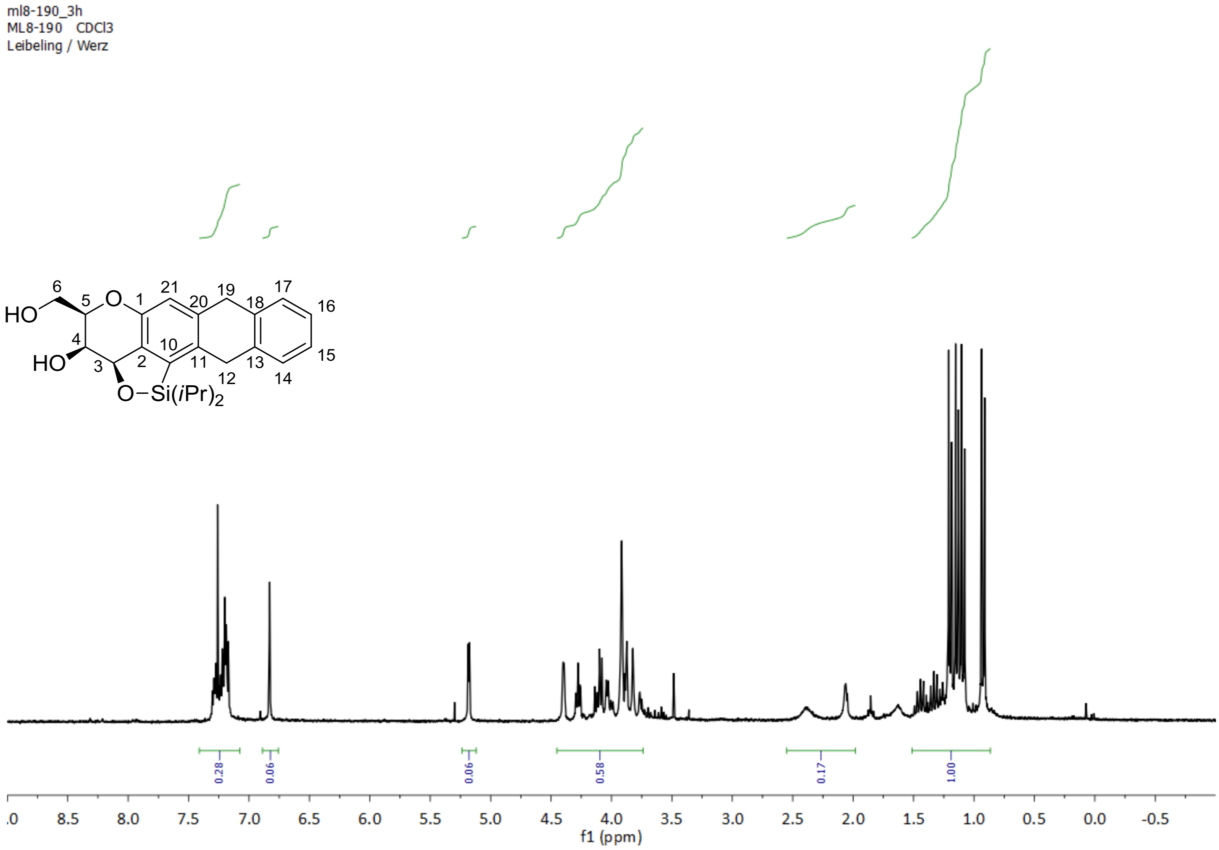

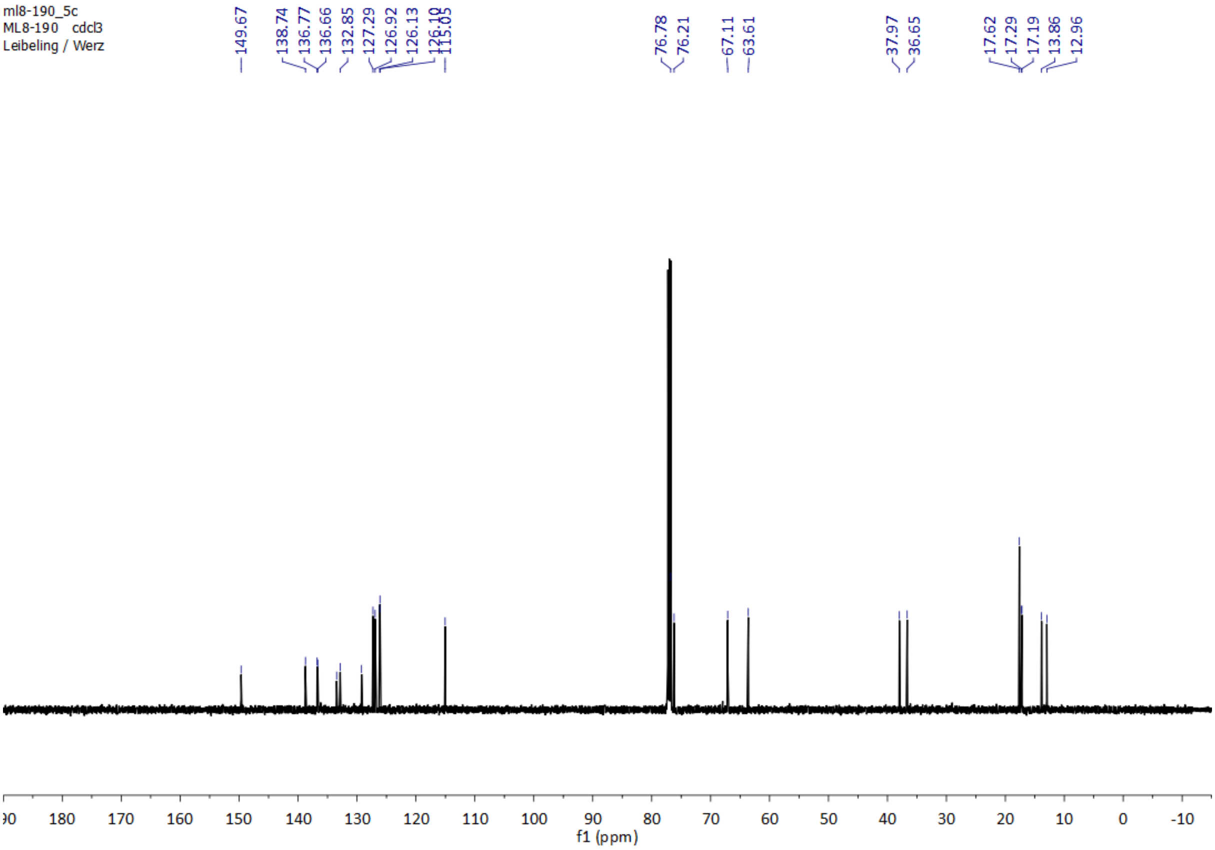

Compound 12b

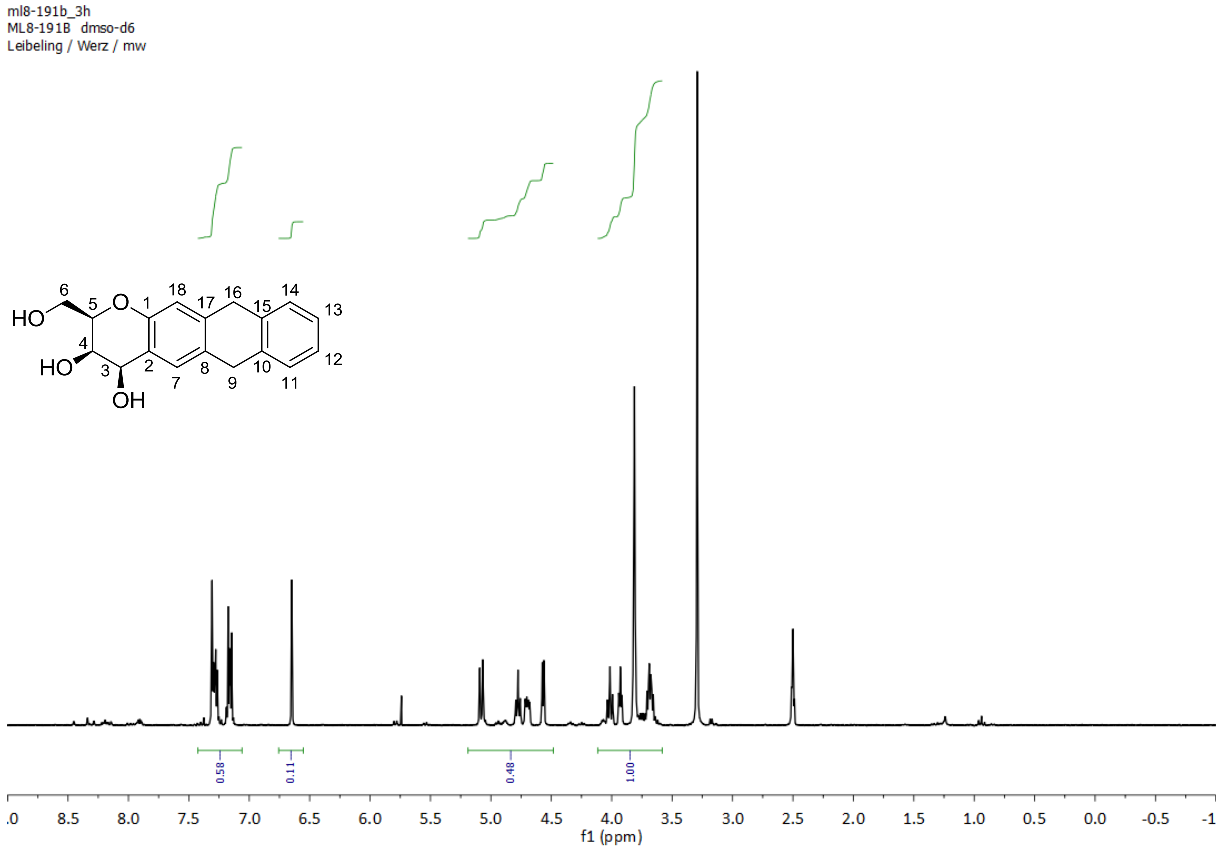

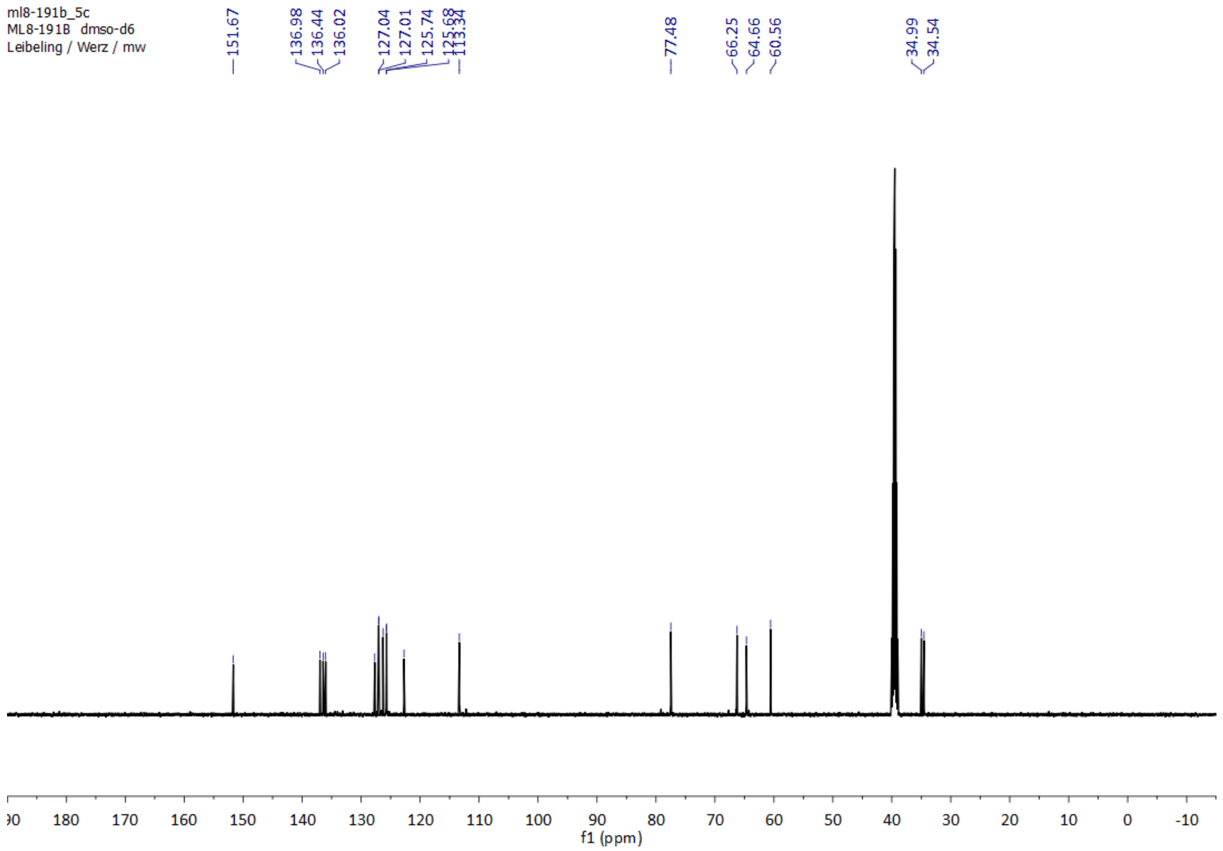

Compound 28b

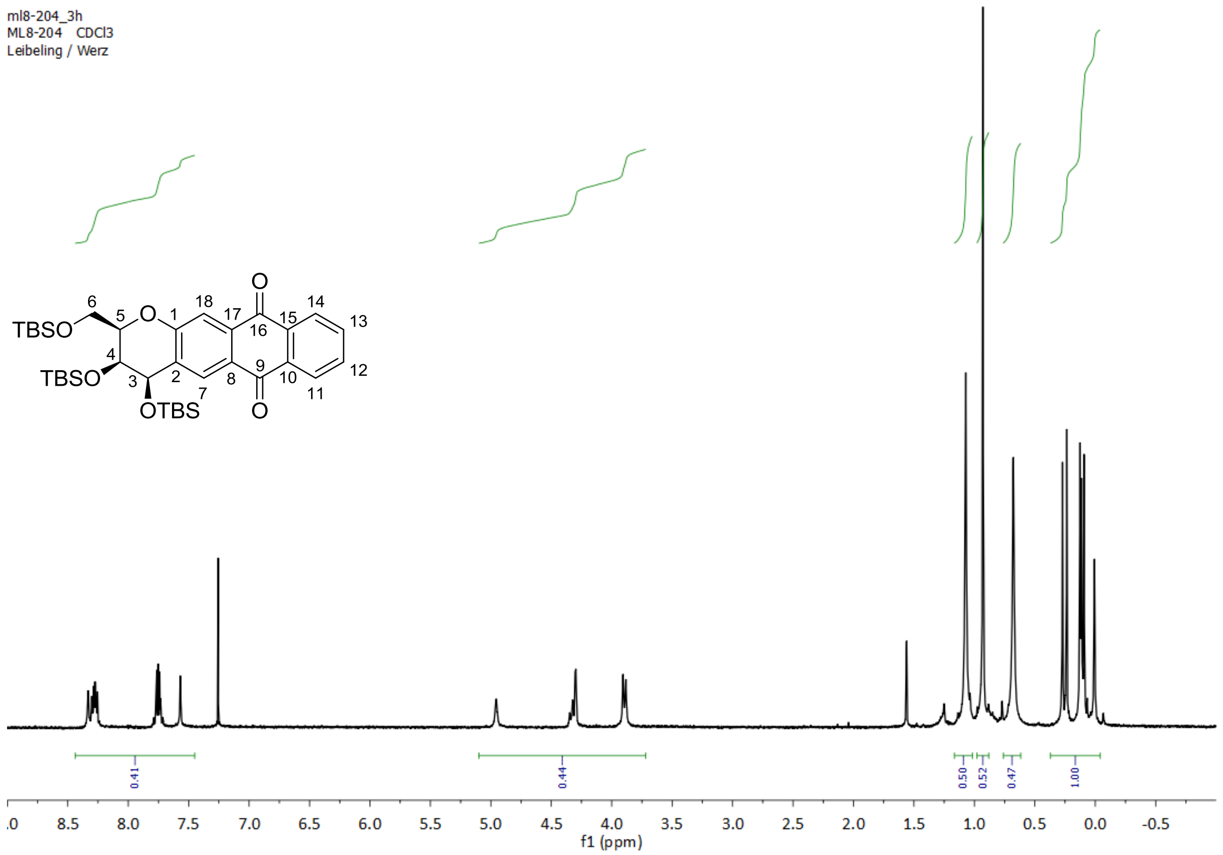

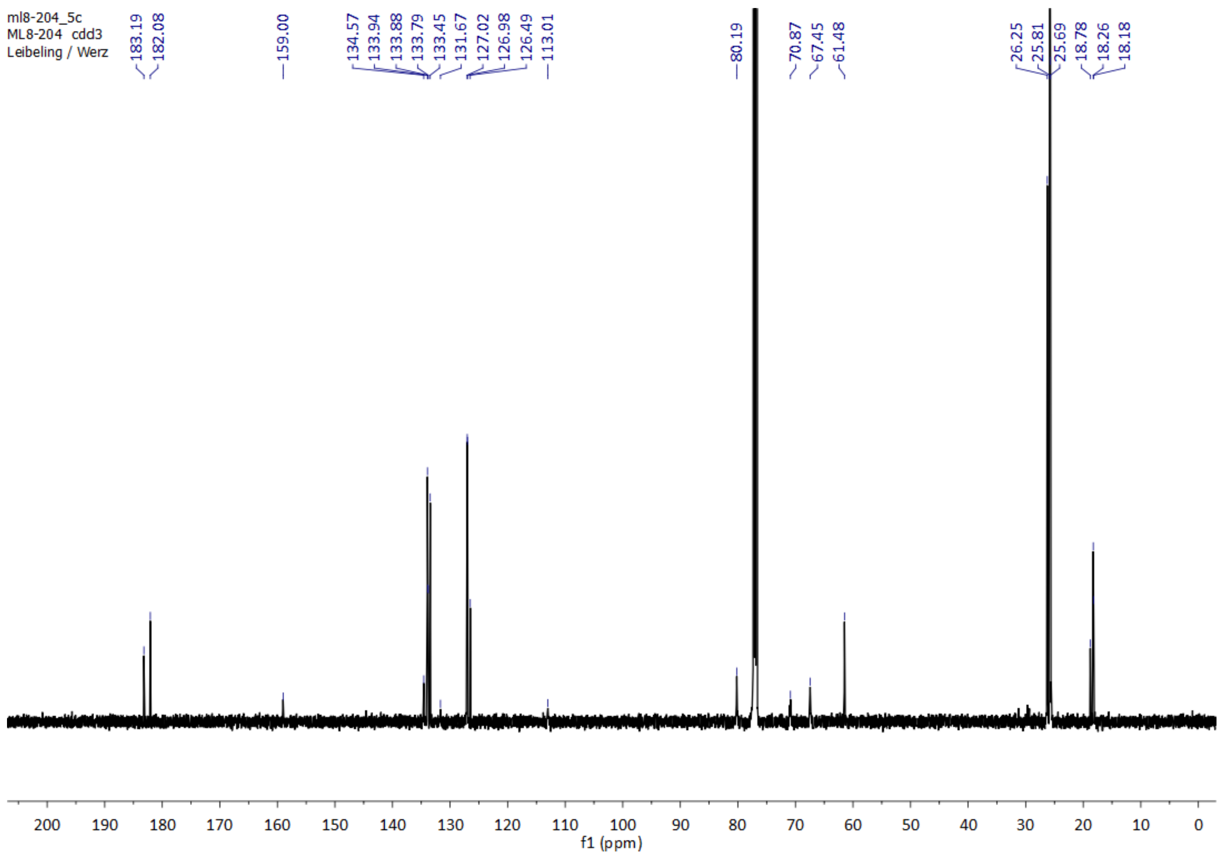

Compound 11b

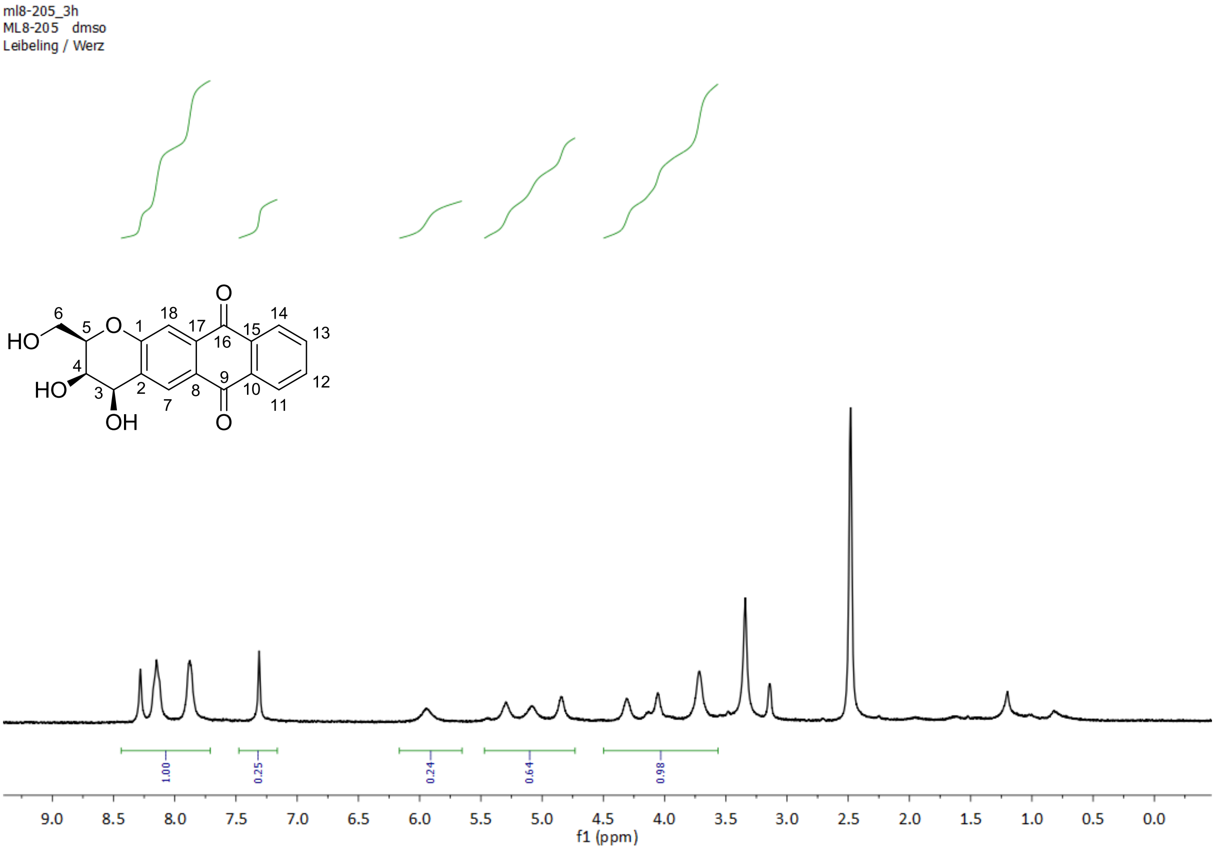

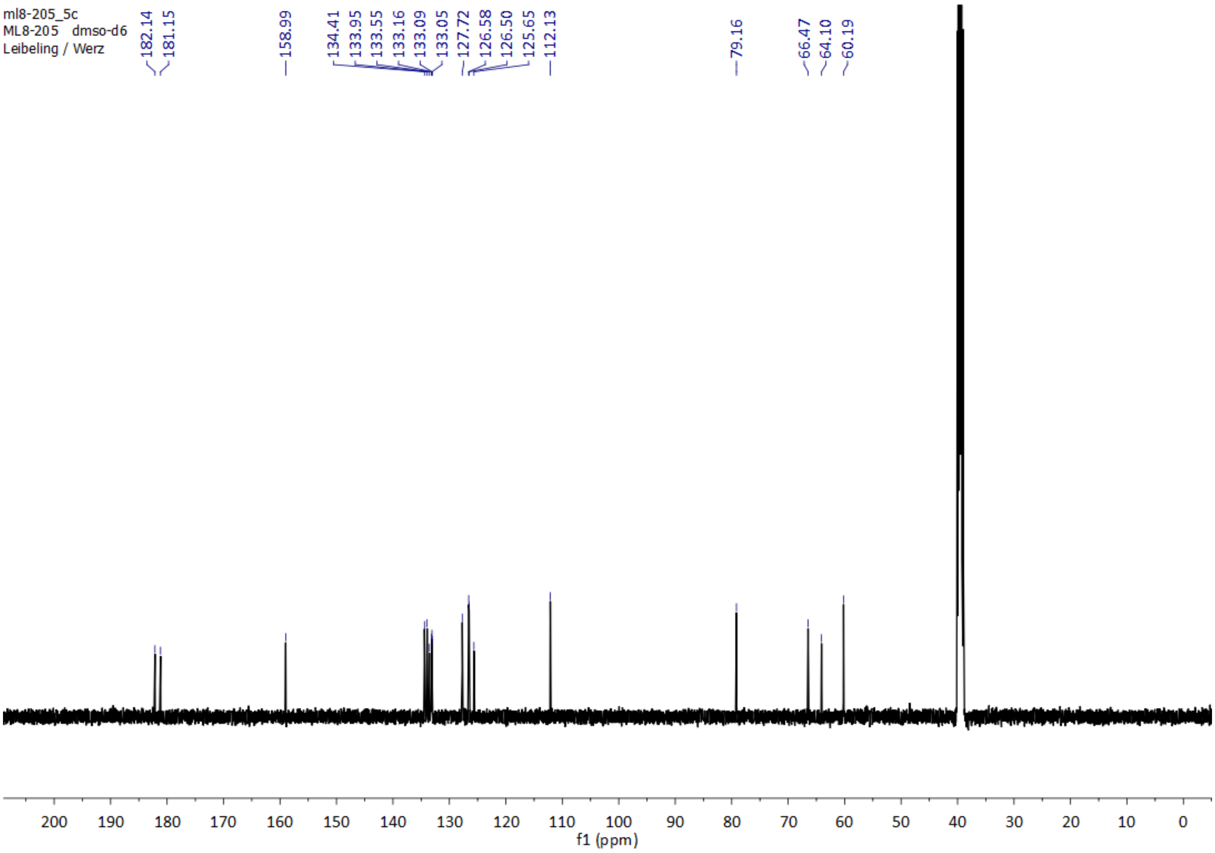

Supplement: File 1 — Experimental details and analytical data of all new compounds as well as their 1H and 13C NMR spectra. [file Beilstein_J_Org_Chem-09-2194-s001.pdf]
